# FusionPHI: A phage-host interaction prediction network model based on attention-driven multi-modal feature fusion

**DOI:** 10.1371/journal.pone.0356238

**Published:** 2026-08-21

**Authors:** Xiangjun Li, Ruitao Li, Zhui Tu, Qingting Wei

**Affiliations:** 1 School of Software, Nanchang University, Nanchang, China; 2 School of Mathematics and Computer Science, Nanchang University, Nanchang, China; 3 Jiangxi Provincial Key Laboratory of Data Security Technology, Nanchang, China; 4 Stated Key Laboratory of Food Science and Resource, Nanchang University, Nanchang, China; Irkutsk State University: Irkutskij gosudarstvennyj universitet, RUSSIAN FEDERATION

## Abstract

Phage therapy has become an important strategy against the crisis of antibiotic resistance for its potential to specifically target pathogenic bacteria. However, the narrow host range of phage makes the screening of precise matches of clinical strains inefficient, while existing computational tools are difficult to capture the dynamics of phage-host interactions due to their reliance on single modal features (genome or proteins). In this paper, we propose FusionPHI, a phage-host interaction prediction model that adopts a fully connected neural network architecture and takes multi-modal features as input, including the k-mer statistics of genome sequences, the physicochemical properties of proteins, and the embedded representations of evolutionarily conserved gene motifs for a comprehensive understanding of phage-host interactions. Moreover, FusionPHI contains a dual-stage attention-drive feature fusion module that integrates a self-attention mechanism to optimize the correlation among the features within a single modality followed by a cross-attention mechanism to dynamically fuse genetic distribution patterns with the protein function information in global or local regions of sequences. The experiments of phage-host interaction prediction show that FusionPHI achieves 91% ROC AUC in cross-validation, demonstrating competitive performance compared to the evaluated baseline methods on our dataset, and ablation experiments further validate the necessity of multi-modal features and attention mechanism. The case study of *E. coli* infected by the *M13K07* phage further validate the prediction ability of the proposed FusionPHI model.

## Introduction

Antimicrobial resistance (AMR) represents a pressing global health challenge, with escalating drug resistance compromising the efficacy of modern medical interventions and contributing to elevated morbidity, mortality, and healthcare expenditures [1,2]. As a promising candidate of targeted antimicrobial approaches, phage therapy has been increasingly recognized as a critical solution to circumvent AMR [3–6].

Phage-host interaction prediction aims to identify potential interactions between phages and their bacterial hosts, which is the key point for screening phages that target specific bacterial strains in phage therapy. Traditional phage-host interaction prediction methods generally rely on laboratory experiments, such as phage spot experiments, which have the advantage of being intuitive, simple, and reliable. However, when it comes to validating the therapeutic potential of phages, a large investment in manpower, time and money is usually required [7].

To address this issue, researchers are exploring the use of the computational biology approach that analyzes genomic information from phages and host bacteria to predict whether an interaction is likely to occur [8] to guide subsequent experimental validation *in vivo* [9,10]. Recent computational phage-host interaction prediction methods fall into two main categories: gene sequence-based methods and protein sequence-based methods. Both methods follow the biological principle that phages and their host bacteria undergo a process of co-evolution, during which they develop ecological co-adaptation at the molecular level and this co-adaptation leaves recognizable imprints or signals in their genome sequences.

Gene sequence-based prediction methods utilize specific patterns in genetic information to make predictions. For example, the VirHostMatcher (VHM [11]) model proposed by Ahlgren et al. evaluates virus-host interactions by analyzing information about different lengths of k-mers. HostPhinder [12] assumed that phages infecting the same bacterial host have the same genomic features, and that the host of the querying phage should be predicted by searching for the phage with the most similar genome in a phage reference database with annotated hosts. Galiez et al. developed the WIsH [13] model, which employs a Markov chain model to parse the gene sequences of phages, which improves the prediction performance of phage-host interaction pairs (PHI). PhiSpy [14] is an open-source software tool for predicting phage regions in the genomes of prokaryotic organisms by finding specific phage gene markers as well as genome signatures to identify potential phage integration sites. vHULK [15] employed annotated genomic features by transforming HMM alignment e-values against the pVOGs database into feature vectors, which were then processed through multilayer perceptron (MLP) models to predict 77 host genera and 118 host species. In recent years, research by [16] has demonstrated, through the integration of large-scale experimental data and machine learning, that genomic information alone can be used to accurately predict phage infectivity at the strain level, and that adsorption factors are the key determinants of interaction.

The second group of computational phage-host interaction prediction methods is based on protein sequence. Since proteins are directly involved in the realization of biological functions, their sequences can often reflect the interaction patterns more accurately. Li et al. proposed PredPHI [17], a deep neural network-based technique specifically designed to predict the type of host a phage may infect from protein sequence data. Another method developed by Li’s team, PHIAF [18], adopts a more comprehensive strategy of combining DNA sequence and protein sequence features via a convolutional neural network (CNN), with a view to further enhancing the overall performance of the PHI prediction model. Notably, a recent approach named RBP-HostPredict employs AlphaFold2 to predict the 3D structures of phage RBPs, extracting electrostatic potential and hydrophobic surface characteristics of their receptor-binding domains (RBDs), and combines molecular docking simulations with host surface receptors (such as LPS and capsular polysaccharides) to predict appropriate hosts [19]. It incorporates structural complementarity scoring into host range prediction. For *Staphylococcus aureus* phages, this approach achieves an 89% accuracy in predicting host strains, demonstrating the value of integrating structural biology into phage-host interaction analysis.

In addition, machine learning and deep learning techniques are widely used in such problems. For example, the PHP [20] system, which uses a Gaussian mixture model for host prediction based on the difference in k-mer frequency distributions between phage and host. VIDHOP [21], an integrated learning framework that combines multiple feature selection strategies with support vector machine algorithms aimed at improving the prediction accuracy of PHIs. iPHoP [22] framework combines outputs from alignment-based methods like BLAST [23] and CRISPR [24] matching, composition-based tools like WIsH and PHP, and protein feature classifiers like RaFAH through a machine learning meta-classifier to generate consensus predictions for metagenome-derived archaeal and bacterial viruses. Furthermore, PB-LKS method [25], which compares the most similar fragments of phages and bacterial genomes through a local k-mer strategy, and for the first time achieved host prediction at the strain mutant level. The ROC-AUC reached 0.86 at the genus level, and the accuracy of the strain experimental verification was 77.5%. In the same year, Wei et al. developed MI-RGC [26], using the mutual information of phages in metagenomic samples to construct a feature-enhanced network, combined with the regional graph convolution model (RGC) to learn multi-level neighborhood relationships, and achieved the highest AUPR of 0.707 on three benchmark datasets. By 2025, Liu et al. launched PHPGAT [27], integrating five types of features such as protein sequences, CRISPR, and RBP to construct a multi-modal heterogeneous knowledge graph.

Although existing methods have achieved notable progress in predicting phage-host interactions (PHIs), several limitations remain. Many approaches mainly rely on global sequence statistics or predefined biological descriptors, which may overlook localized functional patterns embedded in biological sequences. In particular, short conserved subsequences, commonly referred to as sequence motifs, often correspond to functional regions such as binding sites or conserved domains. These motifs reflect localized evolutionary constraints and may provide informative signals for identifying potential phage-host associations.

Recent advances in protein language models provide an alternative way to represent biological sequences. Pretrained models trained on large-scale protein datasets can learn contextual and evolutionary patterns directly from sequences. For example, the ESM-2(Evolutionary Scale Modeling) model [28] generates embedding representations that capture structural and functional properties without requiring explicit structural annotation. Compared with handcrafted descriptors, such representations may provide richer contextual information for downstream prediction tasks. Therefore, using pretrained models to encode conserved motif sequences may offer a more informative representation for PHI prediction.

In addition, attention-based architectures have been widely adopted in biological sequence modeling. The attention mechanism enables models to capture dependencies among sequence elements and dynamically focus on informative regions. Although attention mechanisms have been applied in several biological prediction tasks, their use for integrating heterogeneous biological features in PHI prediction remains limited.

Motivated by these observations, we propose FusionPHI, an attention-driven multi-modal feature fusion network for phage-host interaction prediction. The proposed framework integrates genomic and protein-derived information while incorporating motif-level representations generated by a pretrained language model. By combining complementary biological features, FusionPHI aims to improve the representation of potential phage-host interactions. The main contributions of this paper are as follows:

- **Integration of genome and protein information.** The k-mer statistics of genome sequences and the physicochemical properties of proteins are extracted to be complementary features from both the genomic composition and protein functional levels, providing the model with a more comprehensive biological perspective.
- **Motif-based representation using a pretrained protein language model.** Conserved gene motif sequences are encoded using the pretrained ESM-2 protein language model [28] to capture functional signals that are difficult to represent using traditional sequence descriptors. To the best of our knowledge, this is the first attempt to incorporate motif embeddings derived from a protein language model into phage-host interaction prediction.
- **Dual-stage attention-driven multi-modal feature fusion.** We develop a dual-stage attention-based feature fusion strategy that combines self-attention and cross-attention to integrate heterogeneous biological features. This achieves more effective multi-level feature interaction and fusion, thereby enhancing the model’s discriminative capability. To our knowledge, this framework represents the first application of a dual-attention fusion mechanism for multi-modal feature integration in PHI prediction.

## Materials and methods

### Datasets

**Fig 1:**
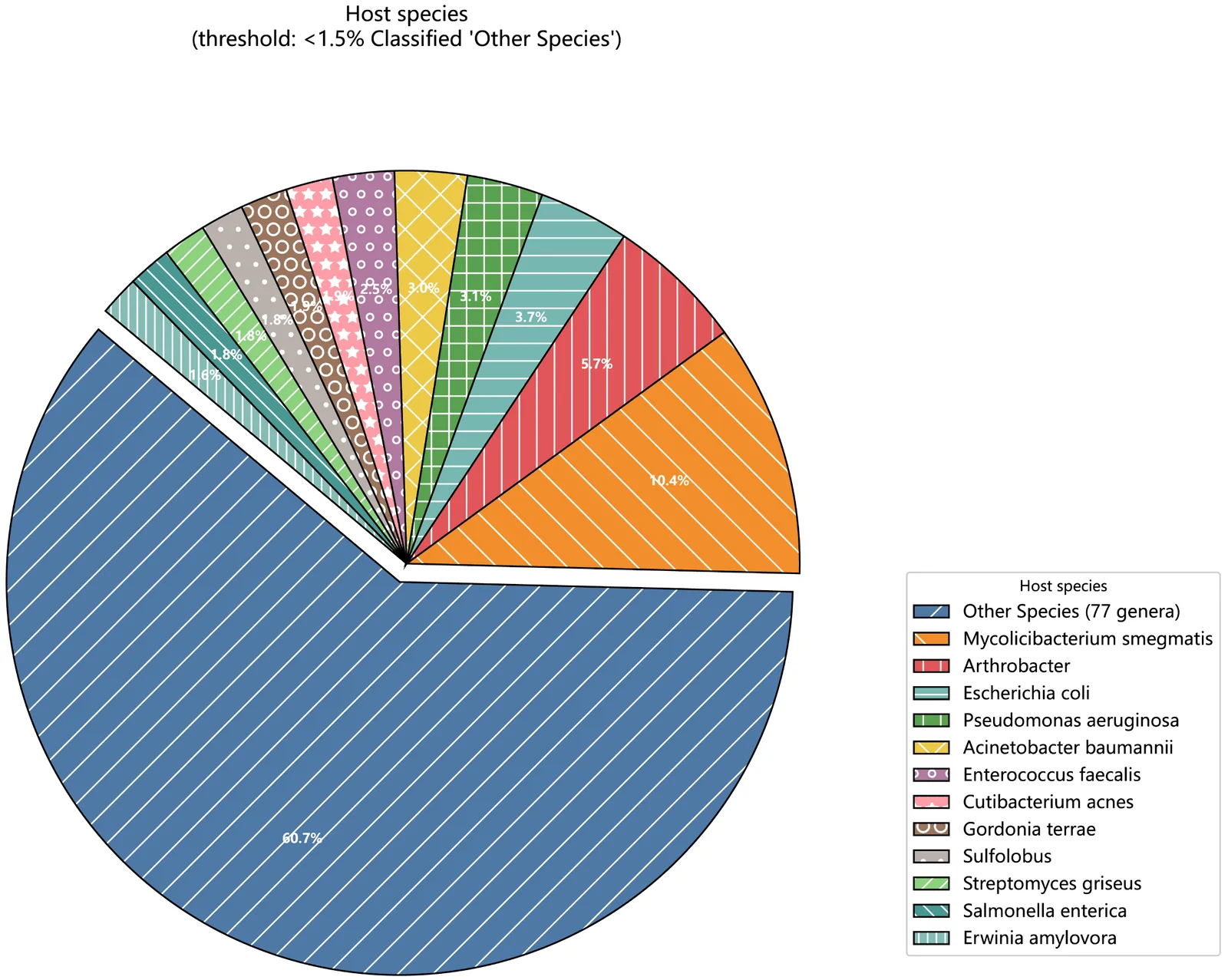
The proportion of host species in the PHP dataset.

**Fig 2:**
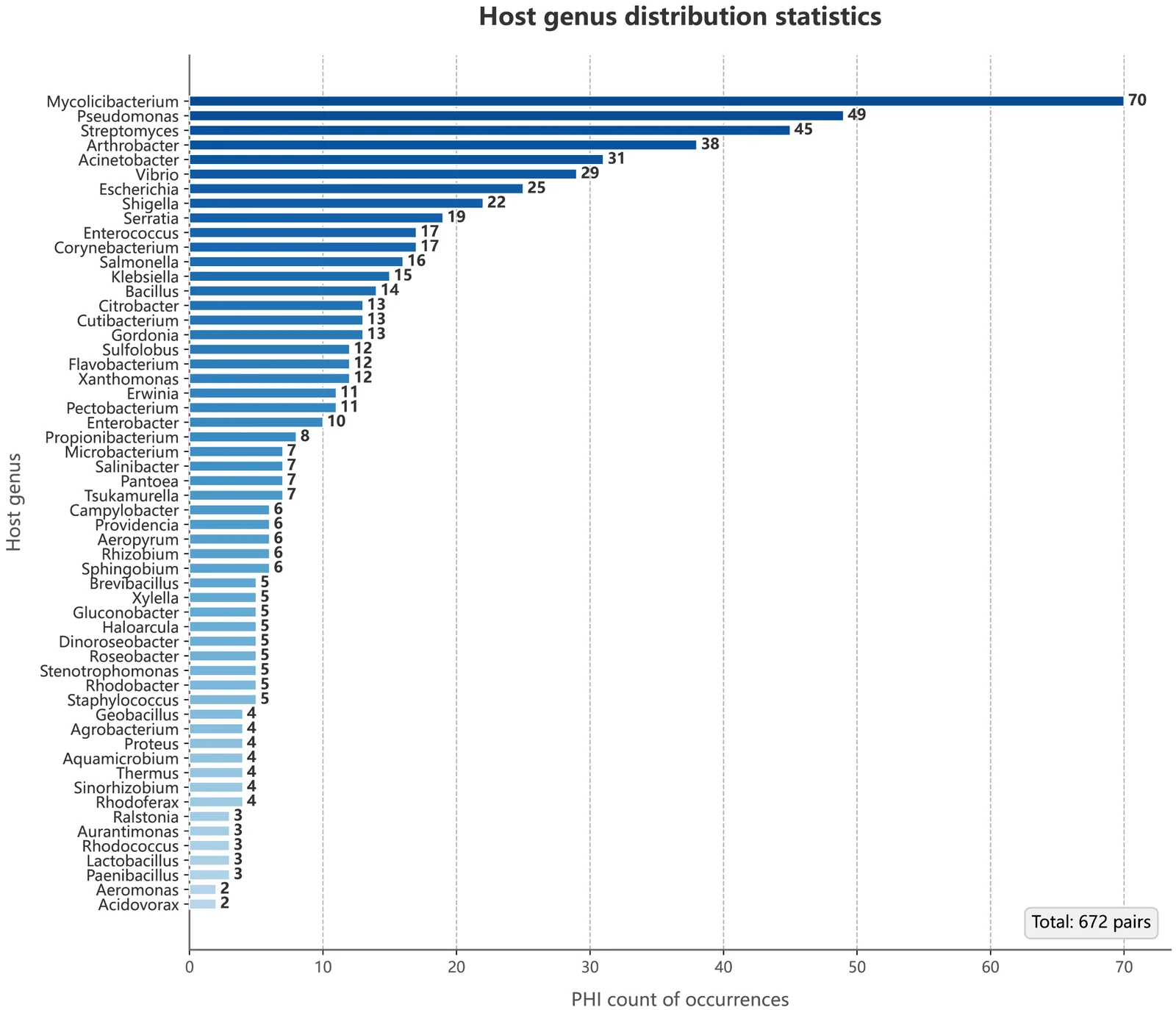
The distribution of the number of samples at the genus level in the PHP dataset host.

We used 670 PHI pairs provided by the PHP study [20]. The ratio of positive samples to negative samples was 1:1. Genomic features of phage and host were extracted for each PHI pair. For the host genome, the scientific nomenclature of bacterial species was extracted from 670 PHI pairs, and 82 target species were obtained after deduplication, and ORF sequences were obtained from NCBI; for the phage genome, 333 phage accession numbers that can be used for direct querying were extracted from 670 PHI pairs and ORF sequences were obtained through them directly. Finally, a dataset containing 82 bacteria genomes and 333 phage genomes was constructed for model training and validating. Meanwhile, a bar chart (Fig 1) was used to visualize the distribution of the number of distinct host genera and the pie chart shown in Fig 2 illustrates the percentage of different host species microorganisms in the dataset. Together, Figs 1 and 2 demonstrate the exact composition of the training dataset.

Fig 1 illustrates the relative proportions of host species at the biological classification level within the dataset. To enhance graph readability, a threshold of 1.5% was applied: species accounting for less than 1.5% of the total were aggregated into the category “Other Species.” The largest segment (blue) corresponds to “Other Species,” which constitutes 60.7% of the total and encompasses 77 distinct genera. This observation reflects a prominent long-tail distribution in the dataset—characterized by a large number of rare host species with limited sample sizes, indicating high diversity and heterogeneity of the data.

Among the independently displayed species, *Mycolicibacterium smegmatis* has the highest proportion (10.4%), followed by *Arthrobacter spp.* (5.7%). Other commonly studied bacteria include *Escherichia coli* (3.7%) and *Pseudomonas aeruginosa* (3.1%).

Secondly, as shown in Fig 2, this horizontal bar chart provides a detailed listing of the specific sample quantities of hosts at the “genus” level of biological classification in the dataset (the number of phage-host pairs). The quantities are sorted from highest to lowest. The genus *Mycolicibacterium* has the highest number of 70 pairs of samples; followed closely by *Pseudomonas* with 49 pairs; *Streptomyces* with 45 pairs; and *Arthrobacter* with 38 pairs. This bar chart visually presents the gradient decreasing trend of sample quantities for each genus. The *Shigella* and *Serratia* at the middle section of the chart have medium-level sample quantities; while at the bottom of the chart, a large number of genera with extremely low sample quantities are concentrated, including *Acidovorax* and *Aeromonas*, which only have 2 pairs of samples each, and genera such as *Lactobacillus* have only 3 pairs of samples.

Besides, we used the PHIAF dataset [18], which contains 235 bacteria genomes and 304 phage genomes as our test dataset. To assess the potential for data leakage between training and testing datasets, a systematic cross-dataset comparison was conducted at both the phage-genome and host -species levels.

#### Phage-genome overlap.

The PHP dataset contains 336 unique phage genomes, whereas the PHIAF dataset includes 304 unique phage genomes. Among them, 18 phage genomes appear in both datasets, corresponding to 5.36% of PHP and 5.92% of PHIAF (Table 1). Because host information is recorded at the species level in PHP and at the genome accession level in PHIAF, even when the same phage genome appears in both datasets, the associated host instances differ. Consequently, no identical phage–host interaction pairs exist at the training-pair level: the 624 PHIAF test pairs were not presented to the model during training.

**Table 1:** Cross-dataset overlap analysis between the PHP training dataset and the PHIAF test dataset.

Metric	Value
Unique phages in PHP	336
Unique phages in PHIAF	304
Overlapping phages (genome-level)	18
Overlap ratio in PHP	5.36%
Overlap ratio in PHIAF	5.92%
Unique host species in PHP	91
Unique host species in PHIAF	128
Overlapping host species	26
Overlap ratio in PHP	28.6%
Overlap ratio in PHIAF	20.3%
Identical (phage, host species) pairs	7
Pair overlap ratio in PHIAF	1.1%

#### Host-species overlap.

To further evaluate the biological overlap of host entities, the host accession identifiers in PHIAF were mapped to their corresponding species names using the host taxonomy table, and a species-level comparison was performed. Table 1 summarizes the results. At the species level, 26 host species are shared between the two datasets (out of 91 PHP species and 128 PHIAF species). Within these overlapping species, 7 completely identical (phage accession, host species) pairs were identified (Table 2), accounting for 7 out of 624 PHIAF test pairs (approximately 1.1%). These pairs arise because the same phage genome was tested against the same host species in both datasets. Taxonomic summaries at the genus and family levels are provided in Table 3 for completeness.

**Table 2:** Seven completely identical (phage accession, host species) pairs identified across the PHP training and PHIAF test datasets at the biological species level.

Phage Accession	Host Species
NC_042081	*Pseudomonas aeruginosa*
NC_042090	*Proteus mirabilis*
NC_042007	*Acinetobacter pittii*
NC_024206	*Lactobacillus gasseri*
NC_042078	*Shigella flexneri*
NC_041921	*Dinoroseobacter shibae*
NC_042139	*Corynebacterium vitaeruminis*
	**Total: 7 pairs**

**Table 3:** Taxonomic-level overlap summary between PHP and PHIAF datasets.

Taxonomic Level	PHP Unique	PHIAF Unique	Overlapping
Species	91	128	26
Genus	56	136	49
Family	18	83	18

Overall, the analysis reveals that 7 identical (phage accession, host species) pairs exist between the PHP training set and the PHIAF test set, which strictly speaking constitutes a minor form of data leakage. However, these pairs account for only 1.1% of the 624 PHIAF test pairs. More importantly, phage–host interaction prediction is inherently a task of learning relational compatibility signals between two genomes, and the discriminative information emerges from the pairing pattern rather than from either genome alone. The PHIAF dataset therefore remains a reasonably independent benchmark for assessing the generalization ability of the proposed model.

To construct a standardized and biologically accurate feature representation, the genomic and proteomic sequence data were strictly retrieved from the NCBI database. Specifically, while the taxonomic classifications and Accession IDs for the interacting phage-host pairs were sourced from the PHP dataset, the actual sequence data were comprehensively re-collected. Using the provided Accession IDs, the complete genome sequences and their corresponding Open Reading Frame (ORF) amino acid sequences were obtained directly from the NCBI RefSeq and GenBank databases. It is important to note that the ORF sequences utilized in this study were strictly based on existing official NCBI annotations. They were either downloaded directly as standard protein FASTA files (.faa) or explicitly extracted from the annotated Coding Sequences (CDS) within the GenBank files. No *de novo* ORF prediction tools were employed. No filtering based on functional annotation was applied. Consequently, the complete set of RefSeq CDS annotations was retained, including sequences annotated as hypothetical proteins. This data acquisition strategy guarantees that the extracted physiochemical features are derived from officially curated coding sequences, thereby avoiding potential computational biases introduced by *de novo* predictions and preserving the high biological validity of the proteomic feature encoding.

### Overview of FusionPHI

**Fig 3:**
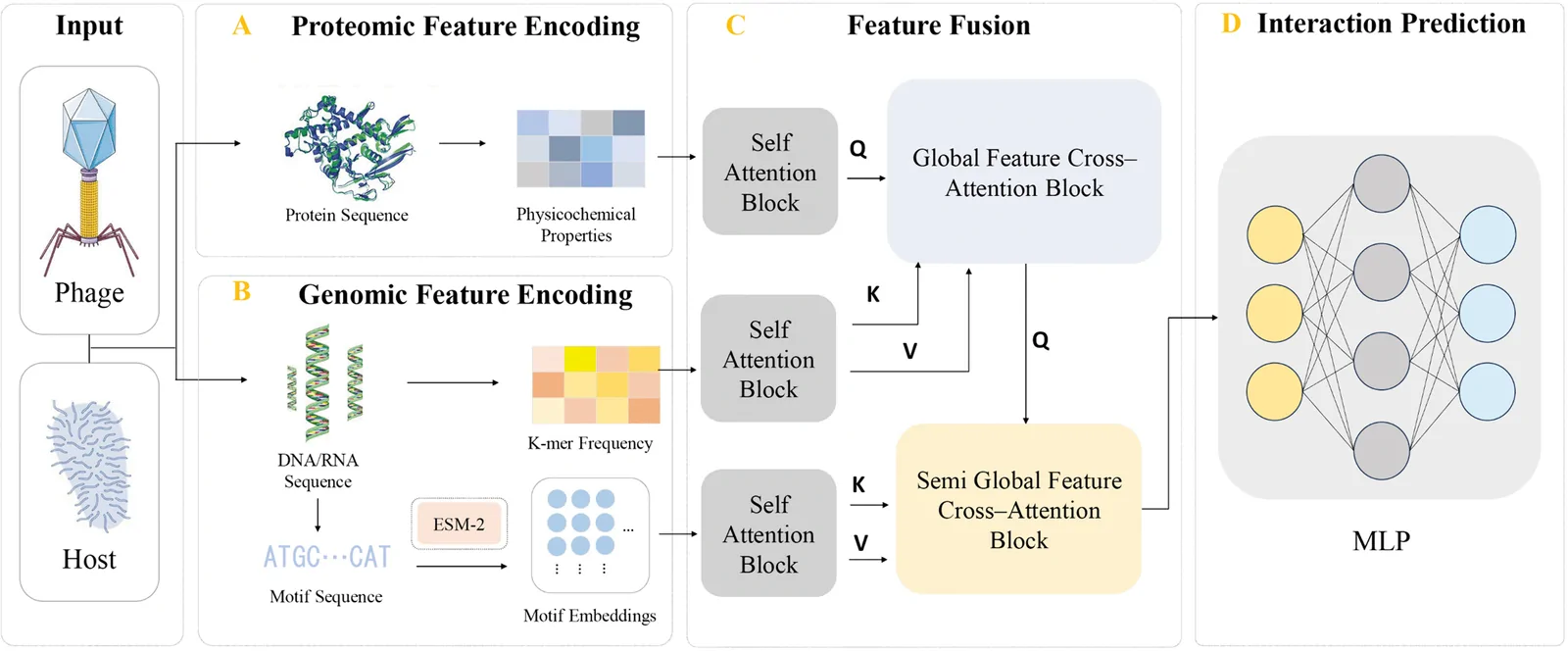
Diagram of the overall FusionPHI architecture. **(A)** The model extracts biological features from genome sequence as well as protein sequence respectively. **(B)** In the genomic feature encoding part, the model processes the genome sequences of phage and host, where the k-mer statistics by sliding-window are generated into the frequency matrix and the gene motif are extracted into high-dimensional embedded representations with the help of the ESM-2 model. **(C)** In the feature fusion section, different modal features are fused using a dual-stage attention mechanism to ensure effective interaction between features. **(D)** In the interaction prediction section, the fused high-dimensional features are fed into the fully connected neural network (MLP) to complete the prediction task of phage-host interaction.

In this paper, FusionPHI is a model that employs a fully connected neural network as an infrastructure and introduces multimodal features as input to comprehensively capture the interaction mechanisms between phage and host. The whole process of the model includes four key components (see Fig 3). Firstly, the physical and chemical properties with functional indicators are extracted from the amino acid sequences transcribed by phage/host open reading frame (ORF). Secondly, the frequency statistical characteristics of different k-mers in phage/host genome sequences were calculated by using sliding window, and the internal conserved motifs of genome sequences were embedded into high-dimensional vectors by ESM-2. Then, a dual-stage attention mechanism was used to realize intra-modality feature optimization and inter-modality dynamic semantic fusion. Finally, the fused high-dimensional features were input into the multi-layer perceptron to complete the phage-host interaction prediction task. The details of each part of the prediction process are discussed in the following sections.

### Protein sequence composition and physicochemical property based proteomic feature encoding

In this study, proteomic features (Fig 3A) are encoded from amino acid sequences. By analyzing physicochemical characteristics of proteins, informative signals related to phage-host interactions can be captured [17,18]. Seven physicochemical properties are considered, including amino acid sequence length, isoelectric point (pI), molecular weight, aromaticity, instability index, flexibility, and hydrophilicity (GRAVY value).

In addition, the amino acid composition (AAC) of protein sequences is calculated by measuring the frequencies of the 20 standard amino acids. Therefore, each Open Reading Frame (ORF) is represented by a total of 27 basic features, consisting of 7 physicochemical indices and 20 amino acid composition frequencies.

To characterize the distribution patterns of these features across ORFs, three statistical measures—mean, variance, and median—are calculated for each feature within a sample. Assuming that the value of the *k*-th feature across *n* ORFs is denoted as $f_{k,1},f_{k,2},...,f_{k,n}$ (*k* = 1,2,...,27), the statistics are defined as follows:

- Mean: Reflects the concentration trend of the feature in the global ORF formula is defined as Eq. 1:
  $$\begin{array}{c} \mu_{k}=\frac{1}{n}\sum_{i=1}^{n}f_{k,i} \end{array}$$ (1)
- Variance: Quantifies the degree of dispersion of the feature across ORFs with the formula Eq. 2:
  $$\begin{array}{c} \sigma_{k}^{2}=\frac{1}{n}\sum_{i=1}^{n}(f_{k,i}-\mu_{k}{)}^{2} \end{array}$$ (2)
- Median: The median of the ordered set $\left\{f_{k,1},f_{k,2},...,f_{k,n}\right\}$ is defined as Eq. 3:
  $$\begin{array}{c} \begin{array}{c} \eta_{k}=\left\{ \begin{array}{cc} & f_{k,\frac{n+1}{2}},\;n=2k+1,k\in \mathbb{Z}\\ & \frac{f_{k,\frac{n}{2}}+f_{k,\frac{n}{2}+1}}{2},\;n=2k,k\in \mathbb{Z} \end{array}\right. \end{array} \end{array}$$ (3)

For each phage sample, the above three statistics are computed for all 27 features, resulting in an 81-dimensional feature vector (27×3). The host protein representation $\begin{array}{c} H_{p}^{<host>} \end{array}$ is computed using the same procedure. Finally, the physicochemical feature vectors of phage and host proteins are concatenated to form the joint proteomic representation.

### K-mer frequency based genomic feature encoding

In the field of biology and bioinformatics, k-mers are consecutive subsequences of length k in a DNA or RNA sequence. In this study, k-mers features were extracted using the Sliding Window Method (SWM), which aims to capture local feature information of genomic sequences(Fig 3B). Using k-mers as an indicator of genetic similarity means that we compare the frequency of occurrence of sequence fragments of the same length in two genomes (in this case the phage genome as well as the host genome). If two genomes share many of the same k-mers, then they are considered to be more similar on a genetic level [12].

In this study, we chose k = 4 for the extraction of genomic k-mer frequency features. In the field of phage-host prediction, mature tools have adopted k-mers of specific lengths as core features. For example, the PHP model [20] uses 4-mers to compare the frequency vectors of phage and host reference genomes. HostPhinder [12] systematically evaluated the impact of different k values on prediction performance during the construction of its method. The results showed that shorter k-mers (such as k = 3) will lead to reduced prediction accuracy due to insufficient specificity, while longer k-mers (such as k = 5 and above) will lead to excessive matching requirements This makes it impossible for many query phages to obtain effective prediction results, so k = 4 is shown to be a reasonable compromise that takes into account both sensitivity and specificity. Furthermore, from a biological mechanistic perspective, tetranucleotide usage patterns have been shown to reflect genomic imprinting left by long-term coevolution between phages and their hosts. Phages from different host ranges show significant conservation in tetranucleotide compositional characteristics, providing a theoretical basis for capturing coevolutionary signals using k = 4 [29]. Based on the above methodological conventions, performance trade-off analysis and biological mechanism support, this study set the k-mer length to 4.

Let the phage and host features be denoted as $\begin{array}{c} F_{k}^{<phage>} \end{array}$ and $\begin{array}{c} F_{k}^{<host>} \end{array}$, respectively, then the corresponding k-mer union feature can be expressed as $\begin{array}{c} H_{k}=concat(F_{k}^{<phage>},F_{k}^{<host>}) \end{array}$

### Gene motif based genomic feature encoding

Motifs are short sequence patterns that frequently repeat in biological sequences, generally carrying specific biological significance. Conserved regions, characterized by highly similar or identical sequence fragments across species or genes, often manifest as motifs. Many of these motifs remain conserved throughout evolution due to their critical roles in regulatory or structural functions. In Phage-Host Interaction (PHI) prediction, conserved regions provide vital clues for revealing functional sequences closely related to interaction mechanisms during evolution.

Based on this premise, this study utilized the MEME suite to identify conserved motif fragments. For a given phage or host genomic sequence *S*, *K* motifs were first identified using the MEME algorithm at the DNA level. It is important to note that, unlike the global proteomic features derived from strict NCBI-curated ORFs, motif identification was purposefully conducted on nucleotide sequences. This design choice aims to capture comprehensive genomic signatures, encompassing both coding sequences and non-coding regulatory elements (e.g., binding sites and promoters) that are critical for mediating phage-host dynamics.

Subsequently, to capture the underlying biochemical syntax of these conserved regions and leverage advanced protein language models, these nucleotide motifs were mapped into the amino acid space utilizing a consistent fixed-frame translation strategy, truncating any incomplete codons to generate the corresponding protein sequence $p_{k}$ (and intermediate RNA sequence $r_{k}$). We acknowledge that this direct conversion without explicit ORF prediction does not necessarily reconstruct functionally active proteins; rather, it serves as a computational feature mapping technique. This design is fundamentally motivated by the fact that evolutionary constraints are primarily manifested at the protein level rather than the nucleotide level. Due to codon degeneracy, distinct DNA sequences can encode identical or functionally similar proteins, making it difficult for pure nucleotide-based representations to consistently reflect functional conservation.

By mapping motif sequences into an amino acid vocabulary, such redundancy is significantly reduced, and sequence variation is better aligned with functional and structural constraints shaped by evolution. The translated sequences are subsequently embedded using the protein language model ESM-2. Pre-trained on massive datasets, ESM-2 exhibits exceptional proficiency in capturing hidden co-evolutionary patterns and high-order contextual dependencies that are indicative of protein structure and function. Therefore, embedding these translated sequence signatures into the ESM-2 representational space provides a more biologically meaningful and discriminative representation compared with directly applying nucleotide-based models such as DNABERT. We can calculate the final motif features $F_{m}$ using the Eq. 4:


$$\begin{array}{c} F_{m}=\frac{1}{K}\sum_{k=1}^{K}v_{k} \end{array}$$ (4)

where *K* denotes the number of motifs detected in the specific sequence, and $v_{k}$ is the embedding vector of the *k*-th motif generated by the ESM-2 model. Finally, the mean-aggregated motif features for the phage and the host, denoted as $\begin{array}{c} F_{m}^{<phage>} \end{array}$ and $\begin{array}{c} F_{m}^{<host>} \end{array}$ respectively, were concatenated to form the union feature, which is defined as Eq. 5:


$$\begin{array}{c} H_{m}=concat(F_{m}^{<phage>},F_{m}^{<host>}) \end{array}$$ (5)

### Dual-stage attention-driven feature fusion

To fully exploit the complementarity and synergy of multi-modal features, this study proposes a Dual-stage Attention-driven Feature Fusion Framework (DAFF). Conceptually, the “attention mechanism” serves as a computational method that allows the model to dynamically assign varying degrees of importance to different parts of the input data. In practice, attention mechanisms operate using three main matrices: a Query (*Q*), a Key (*K*), and a Value (*V*). This can be likened to a database search process: the Query represents the specific target information the model is currently looking for, the Keys represent the descriptive labels or indexes of the available data, and the Values are the actual contents of that data. The model computes a similarity score between the Query and all Keys, which then dictates how much attention (or weight) should be paid to each corresponding Value.

In the DAFF framework, this mechanism operates in two complementary stages. The first stage employs self-attention (where *Q*, *K*, and *V* derive from the same modality) to evaluate internal sequence dependencies. For instance, within k-mer features, it highlights informative local nucleotide patterns while suppressing background noise. The second stage utilizes cross-attention (where *Q* originates from one modality, while *K* and *V* come from another) to construct dynamic semantic mappings across modalities. By using global genomic or proteomic features as *Q* and local conserved motifs as *K* and *V*, the model aligns global evolutionary patterns with relevant localized structures. Ultimately, self-attention refines independent intra-modal representations, and cross-attention bridges them to form a comprehensive biological interaction profile. The following will describe the operation processes of the two attention mechanisms.

#### Self-attention mechanism.

**Fig 4:**
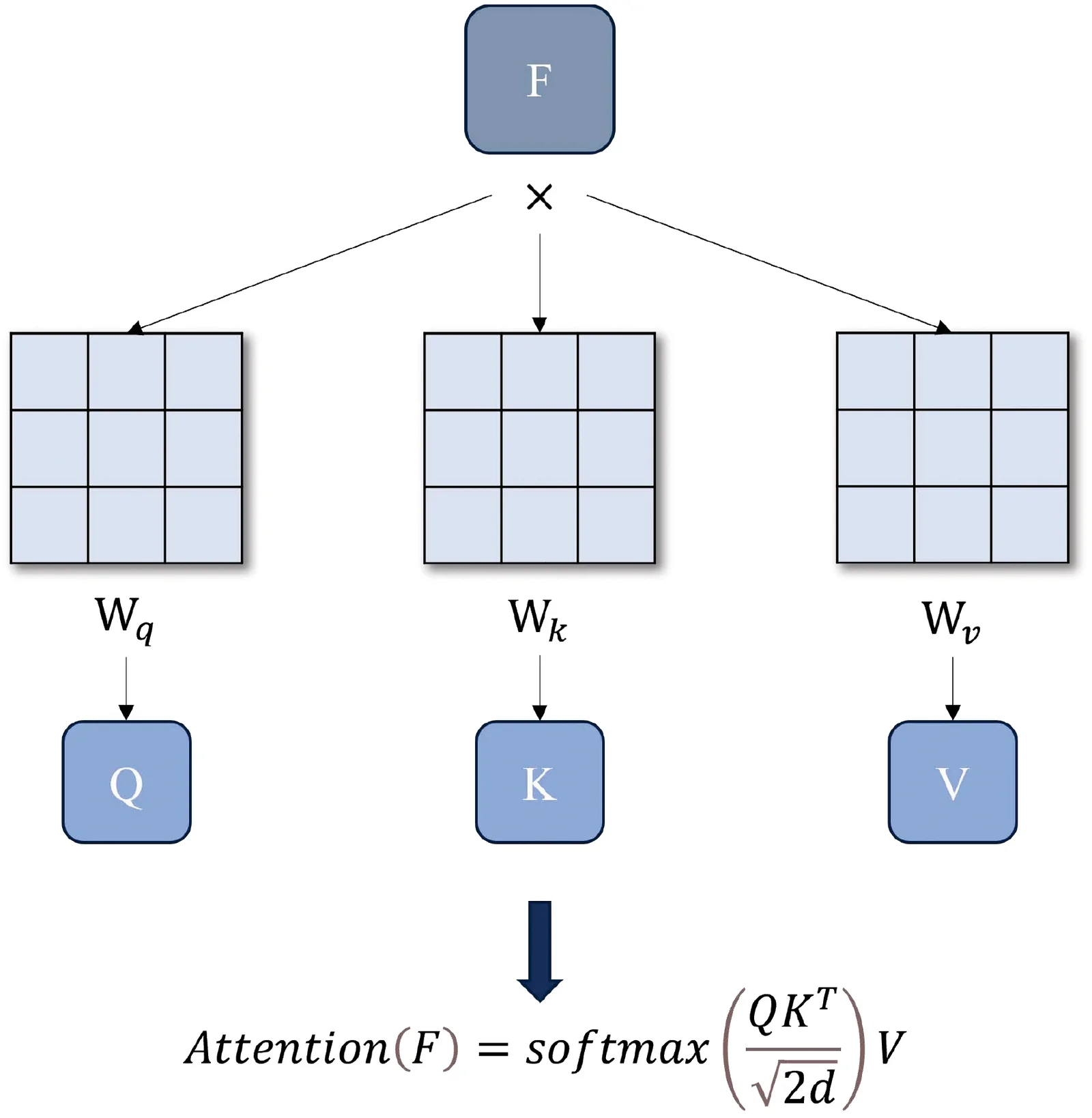
Calculation process of self-attention mechanism.

Before entering the final classification model, the feature representation is optimized using the self-attention mechanism for the joint features of each modality. As shown in Fig 4, if given joint features *F*, the Query (*Q*), Key (*K*) and Value (*V*), they will be obtained by linear transformation, caculated as Eq. 6:


$$\begin{array}{c} Q=FW_{Q},\;K=FW_{K},\;V=FW_{V} \end{array}$$ (6)

where $W_{Q},\;W_{K},\;W_{V}$ is the learnable parameter matrix. After self-attention mechanism [30] the optimized features are represented as Eq. 7:


$$\begin{array}{c} Attention(F)=softmax(\frac{QK^{T}}{\sqrt{2d}})V \end{array}$$ (7)

specifically obtained after applying the feature optimization to the three modes: $\hat{H_{k}}=Attention(H_{k}),\hat{H_{p}}=Attention(H_{p}),\hat{H_{m}}=Attention(H_{m})$.

#### Cross-attention mechanism.

**Fig 5:**
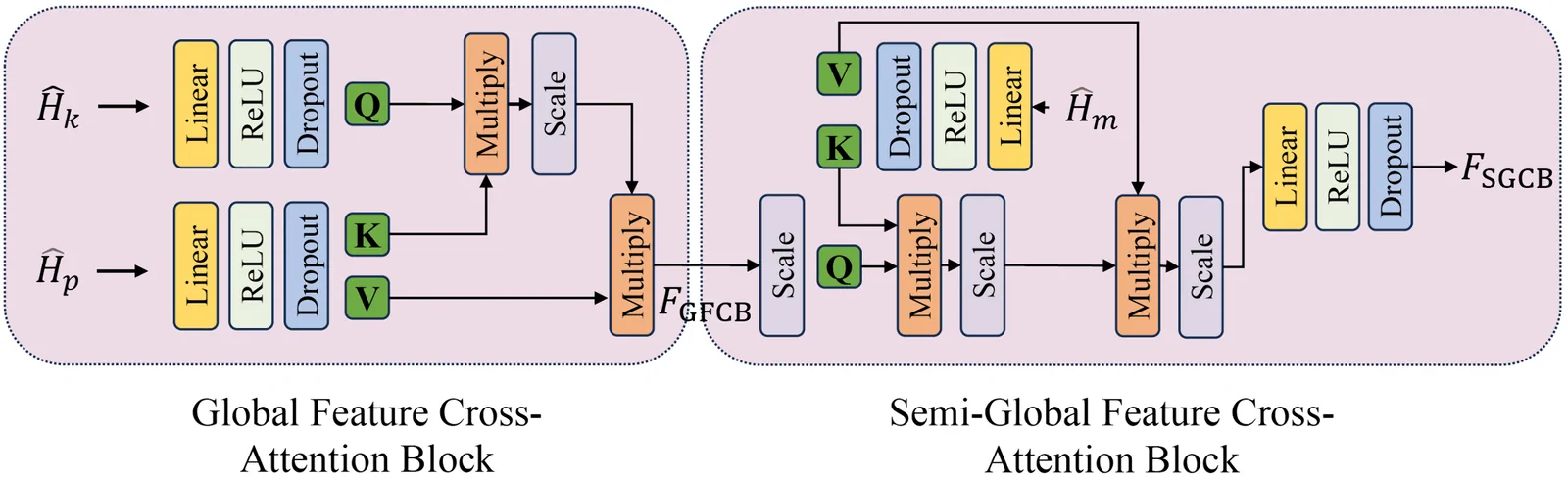
Calculation process of cross-attention mechanism.

After extracting the three modal features and obtaining $\hat{H_{k}},\hat{H_{p}},\hat{H_{m}}$, we used the cross-attention mechanism to fuse the features of different modalities. Cross-attention mechanism is used in two places (Fig 3C), which are Global Feature Cross-Attention Block (GFCB) and Semi-Global Feature Cross-Attention Block (SGCB). The former focuses on the interaction between the global features of the genome and the proteome. The latter focuses on the synergistic validation of “full sequence statistical patterns” and “evolutionarily conserved modules” through secondary attention interactions between motif-embedded representations and global crossover features: the cross-attention mechanism dynamically regulates the expression strength of global features on conserved functional regions, while the sequence specificity of motifs provides evolutionary biological explanations for global statistical patterns. As shown in Fig 5, we can get the result of the GFCB, which is named $F_{GFCB}$:


$$\begin{array}{c} F_{GFCB}=softmax(\frac{\hat{H_{k}}{\hat{H_{p}}}^{T}}{\sqrt{2d_{2}}})\hat{H_{p}} \end{array}$$ (8)

where *d*_2_ denotes the feature dimension of the protein feature. Therefore, this study uses the first cross-attention output $F_{GFCB}$ as the query (*Q*), and the gene motif feature $\hat{H_{m}}$ as the key (*K*) and value (*V*) (*d*_3_ denotes the feature dimension of motif features), the output of the DAFF, named $F_{SGCB}$, can be obtained by the same rule as Eq. 8.

### MLP-based interaction prediction

In the prediction phase of the model(Fig 3D), the fused features $F_{SGCB}$ are processed through a series of fully connected layers and nonlinear activation functions for the final binary classification task, which is defined as Eq. 9.


$$\begin{array}{c} H_{1}=ReLU(F_{SGCB}W_{1}+b_{1}) \end{array}$$ (9)

the *ReLU*() activation function introduces nonlinear capability to enhance the expressive power of the model. In order to reduce the risk of overfitting, the feature *H*_1_ is processed by Dropout, and some neurons are randomly discarded to obtain $\hat{H_{1}}$. Subsequently, the processed features are further downscaled and transformed by another fully connected layer to obtain a deeper feature representation *H*_2_. As shown in Eq. 10, these higher-order features after layer-by-layer projection and nonlinear transformation are input to the classification layer and transformed by linear transformation:


$$\begin{array}{c} \hat{y}=softmax(H_{2}W_{3}+b_{3}) \end{array}$$ (10)

Mapping to the binary classification output space, where *W*_3_ and *b*_3_ are the classifier parameters and the *softmax*( ) function generates the probability distribution of the categories $\hat{y}$.

## Results and discussion

### Experiment setting

We implement the FusionPHI modal based on the PyTorch framework, and adopt a five-fold cross-validation strategy to evaluate the model performance. The dataset was partitioned into five folds using scikit-learn’s KFold(with shuffle = True and random-state = 42 during model training). The learning rate is set to 0.001, the batch size is set to 32 and a Dropout layer (p = 0.5) is introduced to alleviate the overfitting problem. The output of the cross-attention layer is nonlinearly transformed through a fully connected hidden layer that contains 128 neurons, which uses the ReLU activation function to enhance the model representation. The rest of the model parameters (weight initialization, optimizer settings, etc.) follow the default configuration of the PyTorch framework.

### Assessment metrics

Model performance was systematically analyzed using assessment metrics widely used in dichotomous classification tasks, including Accuracy, Specificity, Sensitivity, F1-score, Area Under the Curve of the Subject’s Operating Characteristics (AUC-ROC), and the Matthews Correlation Coefficient (MCC).

### Comparison with existing benchmark methods

First, based on the experimental data of the main models provided in the Nie’s review [7], FusionPHI is compared with the classical methods VHM, PHP, and PHIAF in terms of multiple metrics. It is important to note that conventional baseline methods, such as VHM and PHP, inherently rely on a single genomic feature in their algorithmic design and are therefore incapable of directly accommodating multimodal inputs. Consequently, the comparisons presented in this section aim to evaluate the overall performance differences among various approaches as end-to-end prediction tools. A rigorous controlled-variable analysis to elucidate the specific contributions of individual multimodal features will be conducted in subsequent ablation experiments.

As shown in Table 4, for the metrics reported in the review (Accuracy, Specificity, Sensitivity, and F1-score), FusionPHI consistently demonstrates superior performance on our benchmark dataset. In terms of accuracy, FusionPHI reaches 0.84, which is 23.5%, 55.6%, 42.4%, 27.3% and 3.7% higher than VHM (0.68), PHP (0.54), PHIAF (0.59), PB-LKS (0.66) and PHPGAT (0.81), respectively. To enable a fairer comparison under the same experimental conditions, we additionally re-implemented vHULK (denoted as vHULK-run) and evaluated all six metrics. vHULK was selected for this re‑evaluation because it is the only baseline tool that shares a deep learning framework and Python implementation with FusionPHI, allowing for a controlled comparison under identical computational settings. Under this setup, FusionPHI achieves an AUC of 0.86 and an MCC of 0.66, substantially outperforming vHULK-run (AUC: 0.52, MCC: 0.03). The AUC and MCC values for the remaining methods are unavailable from the original review and are thus left blank in the table.

**Table 4:** Comparative experimental analysis.

Method	AUC	MCC	Accuracy	Specificity	Sensitivity	F1-score
VHM	–	–	0.68	0.97	0.39	0.54
PHP	–	–	0.54	**1.00**	0.08	0.14
PHIAF	–	–	0.59	0.96	0.73	0.68
vHULK	–	–	0.43	0.73	0.12	0.18
PB-LKS	–	–	0.66	0.69	0.62	0.64
PHPGAT	–	–	0.81	–	–	–
vHULK-run	0.52	0.03	0.52	0.59	0.44	0.48
FusionPHI (ours)	0.86	0.66	**0.84**	0.83	**0.85**	**0.83**

Note: Values for VHM, PHP, PHIAF, PB‑LKS, and PHPGAT are adapted from Nie et al [7]. and AUC and MCC were not reported for these methods. vHULK‑run indicates our independent re‑evaluation under identical conditions as FusionPHI. Best results are in bold.

Existing methods show clear limitations in balancing sensitivity and specificity for PHI prediction. In contrast, FusionPHI dynamically integrates multimodal signatures through a dual-stage attention mechanism, which enhances pattern-based functional module recognition while preserving global sequence patterns. It consistently outperforms recent methods (vHULK, PB-LKS, and PHPGAT) across comprehensive metrics. Notably, compared with the next best model PHIAF, FusionPHI raises the sensitivity from 0.73 to 0.85 and improves the F1-score from 0.68 to 0.83. The FusionPHI framework achieved a balanced breakthrough in sensitivity and specificity, providing a potential alternative to traditional approaches that primarily rely on a single statistical or functional model.

**Fig 6:**
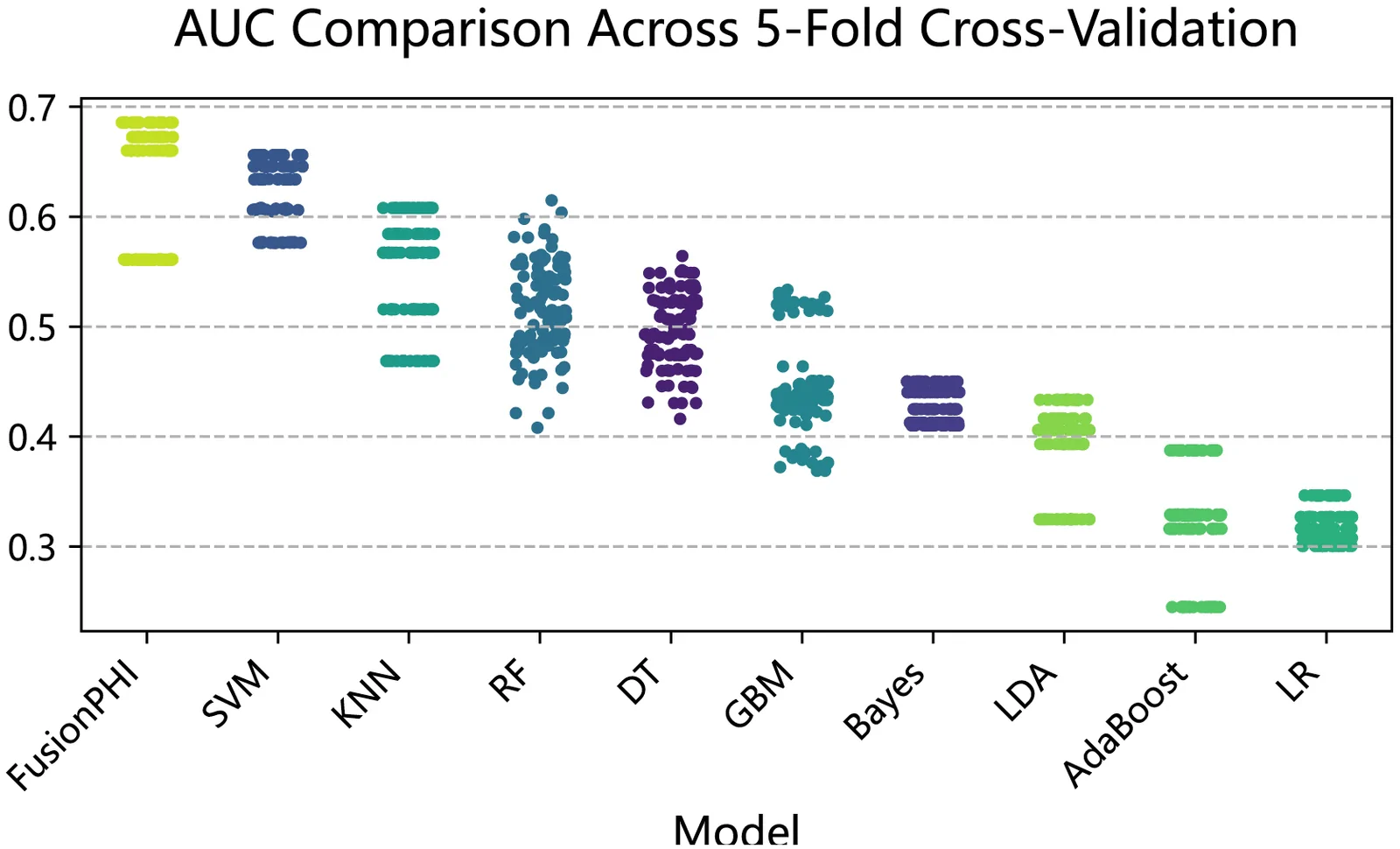
Performance comparison of FusionPHI and classical machine learning models on the PHIAF dataset. Individual data points represent the exact AUC scores obtained across the 5-fold cross-validation.

To verify the generalization ability and robustness of the FusionPHI model, this study compares its AUC performance against nine classical machine learning models on the PHIAF dataset (see Fig 6). The experiment adopts a five-fold cross-validation strategy. To ensure full data transparency and avoid the potential misrepresentations associated with small-sample box plots, we explicitly visualize the exact AUC scores of each fold as individual data points. This visualization provides a clearer and more objective assessment of the performance distribution and stability of each model. Traditional models often struggle to capture the complex nonlinear dynamics of PHI. While ensemble methods like RF and GBM offer some improvements through feature combinations, they are frequently constrained by the representational depth of their inputs. FusionPHI addresses these challenges through a systematic multi-modal feature engineering process, extracting comprehensive biological representations from genomic compositions, protein functional properties, and evolutionary motifs. By employing a dual-stage attention-driven fusion strategy, the model effectively characterizes the hierarchical relationships and inter-modal synergies essential for accurate interaction prediction. These structural innovations enable FusionPHI to achieve competitive and stable results, culminating in a 10.0% improvement in average AUC over the strongest baseline approaches, which suggests that FusionPHI could serve as a robust computational tool to assist in phage therapy research.

### Ablation study

In order to verify the contribution and irreplaceability of different feature modalities, an ablation analysis was conducted by constructing several incomplete feature subsets (see Table 5). Following the principle of controlled variables, the dual-stage attention architecture was retained while individual feature modalities were sequentially removed to evaluate their influence on phage-host interaction prediction performance. To improve the robustness and reliability of the evaluation, each ablation experiment was independently repeated 20 times under the same experimental settings, and the mean performance together with the standard deviation across runs was reported.

- Attn-KM (k-mer + motif only): removal of protein physicochemical properties decreased the specificity from 0.93 to 0.66 (Δ=−27%) and reduced the F1-score from 0.81 to 0.76 (Δ=−5%).
- Attn-KP (k-mer + physicochemical properties only): removal of the motif feature reduced the F1-score from 0.81 to 0.72 (Δ=−9%) and decreased the AUC from 0.87 to 0.84 (Δ=−3%).
- Attn-PM (physicochemical properties + motif only): after excluding the k-mer frequency feature, the AUC decreased from 0.87 to 0.82 (Δ=−5%), while the sensitivity increased from 0.72 to 0.81 (Δ=+9%).

**Table 5:** Ablation study results based on 5-fold cross-validation (mean ± standard deviation).

Model	Accuracy	Specificity	Sensitivity	F1-score	MCC	AUC
Attn-KP	0.73±0.021	0.79±0.037	0.67±0.028	0.72±0.019	0.49±0.026	0.84±0.018
Attn-KM	0.75±0.026	0.66±0.048	0.83±0.025	0.76±0.021	0.53±0.029	0.76±0.024
Attn-PM	0.79±0.019	0.77±0.031	0.81±0.027	0.79±0.018	0.61±0.021	0.82±0.017
Pred-S	0.79±0.018	0.78±0.029	0.79±0.026	0.78±0.017	0.62±0.020	0.83±0.016
Pred-C	0.77±0.024	0.71±0.041	0.82±0.029	0.78±0.022	0.57±0.027	0.81±0.019
Our Model	0.83±0.015	0.93±0.018	0.72±0.024	0.81±0.016	0.71±0.018	0.87±0.012

In order to further resolve the synergistic mechanism of the dual-stage attention mechanism, this study assesses the differential contribution of the two to feature representation learning by decoupling the self-attention and cross-attention layers.

- Pred-S (self-attention only): after disabling the cross-attention layer, MCC decreased from 0.71 to 0.62 (Δ=−9%) and specificity decreased from 0.93 to 0.78 (Δ=−15%).
- Pred-C (cross-attention only): removing the self-attention layer reduced the AUC from 0.87 to 0.81 (Δ=−6%) and decreased the MCC from 0.71 to 0.57 (Δ=−14%).

The ablation study further validates the necessity of both multi-modal feature integration and the dual-attention mechanism. Systematic removal of individual features, including k-mer frequency, physicochemical properties, and motif embeddings, resulted in noticeable performance degradation across multiple evaluation metrics. In particular, excluding motif embeddings led to a 9% reduction in the F1-score, highlighting the complementary contribution of different biological feature representations in characterizing phage-host interactions. Similarly, decoupling the self-attention and cross-attention modules revealed their synergistic roles in feature representation learning. Self-attention primarily captures intra-modal dependencies, whereas cross-attention facilitates inter-modal biological association modeling. The isolated use of either attention mechanism resulted in clear declines in MCC (9%–14%) and AUC (3%–6%), demonstrating that the integration of both mechanisms is important for maintaining robust predictive performance.

### Attention-based Feature interaction analysis

Understanding which biological features contribute most to phage-host interaction prediction is essential for interpreting model behavior and gaining biological insights into the molecular determinants of host range specificity.

**Fig 7:**
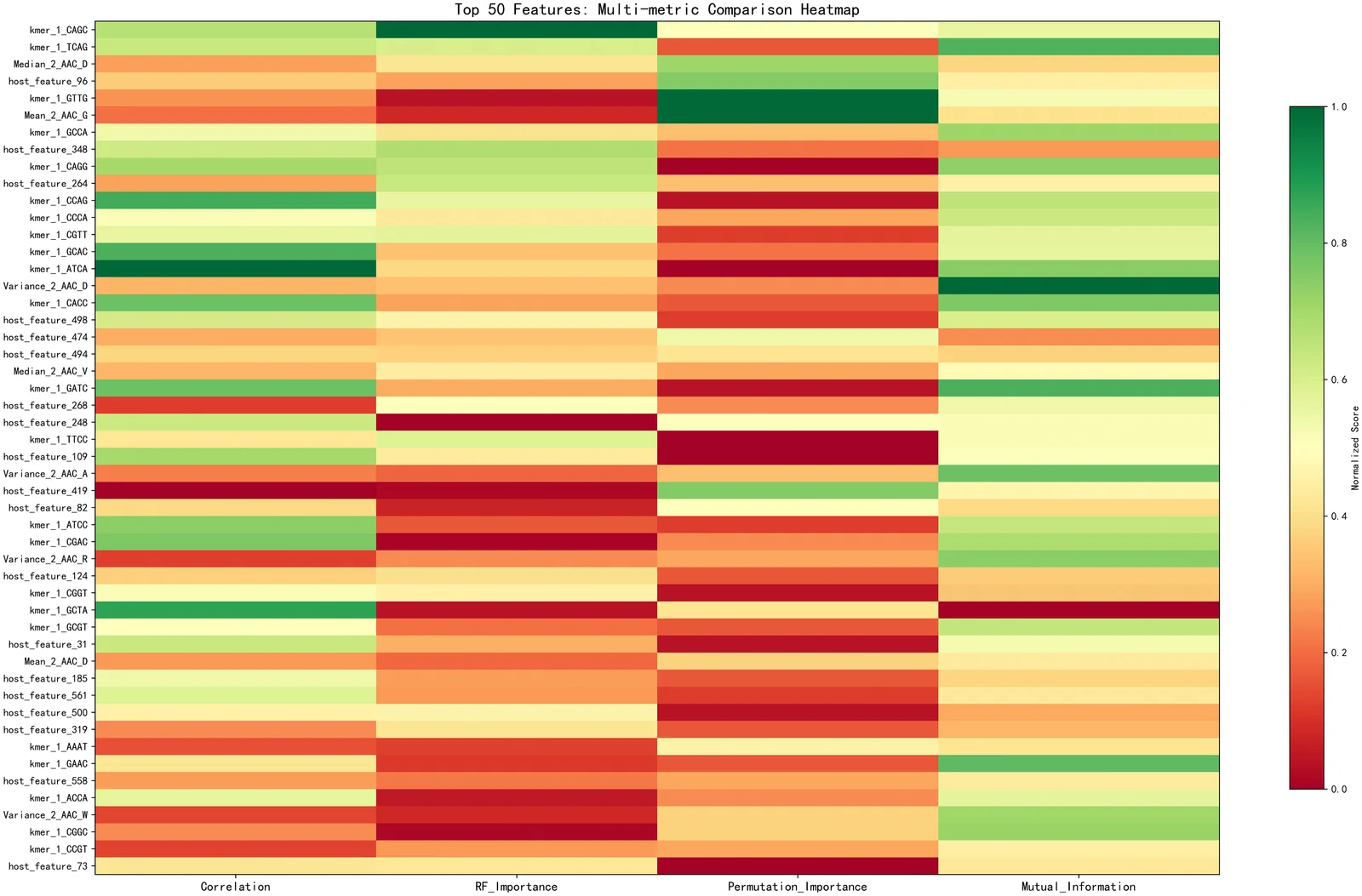
Multi-metric feature importance heatmap of top 50 ranked features. Rows represent the top 50 features ordered by combined importance score; columns represent four evaluation metrics (Correlation, RF Importance, Permutation Importance, Mutual Information). Color intensity indicates normalized importance scores from 0 (red) to 1 (green).

To quantify feature importance in a robust and interpretable manner, we employed a multi-metric evaluation framework that integrates four complementary approaches: (1) Pearson correlation coefficient measuring linear association between features and interaction labels; (2) Random Forest Gini importance derived from 100 decision trees via Mean Decrease in Impurity (MDI); (3) Permutation importance computed as prediction performance degradation after random feature shuffling; and (4) Mutual Information assessing non-linear statistical dependency between features and labels. All raw importance scores were normalized to the [0,1] interval using min-max scaling, enabling direct comparison across heterogeneous metrics. The normalized heatmap (see Fig 7) visualizes the top 50 ranked features, with rows representing individual features and columns representing the four evaluation metrics.

As shown in Fig 7, the heatmap is ordered by the descending mean of the four normalized evaluation metrics (Correlation, RF_Importance, Permutation_Importance, and Mutual_Information), i.e., the combined score. The colour scale ranges from green (high values) to yellow/red (low values). The features are classified into three types: k-mer sequence features (e.g., kmer_1_CAGC representing the frequency of the k-mer CAGC in the phage sequence), protein physicochemical features (e.g., Variance_2_AAC_D, Median_2_AAC_D, and Mean_2_AAC_D representing the variance, median and mean of the amino acid composition (AAC) frequencies for the amino acid *D* in all possible ORFs of the host sequence), and host-derived motif features (e.g., host_feature_96 representing the 96th element of the host motif embeddings). The integers 1 and 2 denote the features of phages and hosts respectively.

The heatmap reveals marked discrepancies among the four metrics in evaluating feature importance. For example, the top‑ranked kmer_1_CAGC (row 1) exhibits the highest Random Forest importance (normalized value 1.0), yet its permutation importance and mutual information are moderate. kmer_1_TCAG (row 2) shows a particularly high mutual information score (0.889) but low permutation importance. Median_2_AAC_D (row 3) and host_feature_96 (row 4) display modest correlation scores but relatively high permutation importance (0.708 and 0.75, respectively), suggesting that these features capture unique predictive signals beyond linear associations. Notably, kmer_1_GTTG (row 5) achieves a full permutation importance score (1.0) while having very low Random Forest importance (0.043), indicating that this feature may be influential under perturbation yet easily overlooked by tree‑based models.

Among physicochemical features, Variance_2_AAC_D (row 16) stands out with a mutual information score of 1.0 but lower scores on other metrics, highlighting its strong non‑linear predictive capacity. Conversely, kmer_1_ATCA (row 15) has the highest correlation (1.0) but zero permutation importance, implying potential collinearity with other features. Overall, k‑mer features show considerable variability across Random Forest and mutual information metrics, while motif features tend to display balanced, moderate scores (yellow‑green) across all measures, indicating a stable and distributed contribution to the prediction task.

CG-enriched k-mer sequence features (e.g., the frequencies of CAGC, TCAG, CAGG, CCAG) dominated the top-ranking positions and achieved consistently high scores across all four evaluation metrics, indicating that these local nucleotide patterns serve as robust discriminative features for phage-host interaction prediction. The agreement among different metrics supports the view that these features capture genuine predictive signals rather than artifacts of any single metric. Physicochemical features derived from amino acid compositions (e.g., the AAC frequencies for the amino acids *D* and *G*) showed a contrasting pattern: they exhibited only moderate Pearson correlations but notably high permutation importance. This discrepancy suggests a non-linear relationship between these features and the interaction labels, meaning that tree-based models and information-theoretic measures can capture predictive value that linear correlation fails to detect. In practical terms, this finding justifies our multi-metric evaluation approach, as relying solely on correlation-based selection would have overlooked features that contribute substantially through non-linear mechanisms. Protein domain features (e.g., host_feature_96, host_feature_348, host_feature_264) displayed yet another pattern, characterized by balanced, moderate importance across all metrics. Rather than being driven by a single dominant signal, their contribution appears to be distributed, implying that domain-level features provide complementary information at a different level of abstraction. Taken together, these results confirm that effective phage-host interaction prediction benefits from combining heterogeneous feature modalities.

### Case study

In order to validate the prediction ability of the theoretical model on phage-host interactions, we constructed a complete ‘prediction-validation’ closed loop by comparing the experimental observations with the results of the model in this study. The selection of *E. coli* and *M13K07* phage for this case study is based on their well-characterized interactions, where M13 phage has been extensively utilized as a signal amplification container for the sensitive detection of live *E. coli* cells [31], highlighting its specificity and reliability as a model system. Crucially, this setup provides a robust independent evaluation of the model’s generalization capabilities. While the broad species *E. coli* exists in our training dataset, it constitutes a minor fraction, representing only 3.7% of the species distribution with 25 interaction pairs. Furthermore, the specific engineered strain variants and the helper phage *M13K07* utilized in this case study were completely absent from the training phase. Consequently, successful predictions in this context demonstrate that the model relies on true generalization based on learned biological features, rather than mere data memorization.

Through specific infestation experiments, we attempted to answer the question of whether the host incompatibility predicted by the model can be confirmed in real biological systems. To achieve this, host strain selection covered four representative strains: *BL21(DE3)*, *DH5*α, *Rosetta*⋆*(DE3)pLys*, and *TG1*. These strains were deliberately chosen to establish a biologically rigorous setup with built-in positive and negative controls at the sub-species level. Infection by M13-derived phages strictly requires the presence of the F pilus on the host cell surface; among the selected cohort, *TG1* carries an F’ episome, making it a permissive host, whereas *BL21(DE3)*, *DH5*α, and *Rosetta*⋆*(DE3)pLys* lack the F factor and are naturally non-permissive. By utilizing these distinct genetic backgrounds, we can critically evaluate the model’s ability to discern nuanced, strain-level genomic differences.

The experiments were carried out using a technical route combining the gradient dilution method with empty spot counting. Four concentration gradients (10^8^, 10^9^, 10^10^, and 10^11^ -fold dilutions) were prepared by serial dilution of phage suspensions with an initial titre $7\times 10^{13}pfu/mL$. Host strain selection covered 4 representative strains of *BL21(DE3)*, DH5α, Rosetta⋆(DE3)plys and *TG1* to assess the infection characteristics of different host systems. Each strain was pre-cultured in LB medium with shaking until logarithmic growth phase ($OD_{600}\approx 0.6$), and then 100μL of the bacterial solution was mixed with 10μL of gradient-diluted phage suspensions under aseptic conditions, and the adsorption was allowed to stand for 20 min. The infection complex was transferred to $47^{\circ }C$ prewarmed upper agar containing 5mM *MgCl*_2_ which was rapidly decanted onto the surface of $37^{\circ }C$ pre-equilibrated LB solid medium. After solidifying the agar, the plates were inverted and incubated for 12–16 h at $37^{\circ }C$ in a constant temperature incubator. Infection efficiency was finally determined by visual counting of plaque-forming units (*pfu*), and the experiment was set up with three biological replicates to ensure the reliability of the data. The results showed that under the above experimental conditions, no empty spot formation was detected in any of the four host strains (*pfu* = 0), indicating that *BL21(DE3)*, DH5α and Rosetta⋆(DE3)plys were unable to undergo specific binding and cleavage reactions with phage *M13K07*.

**Fig 8:**
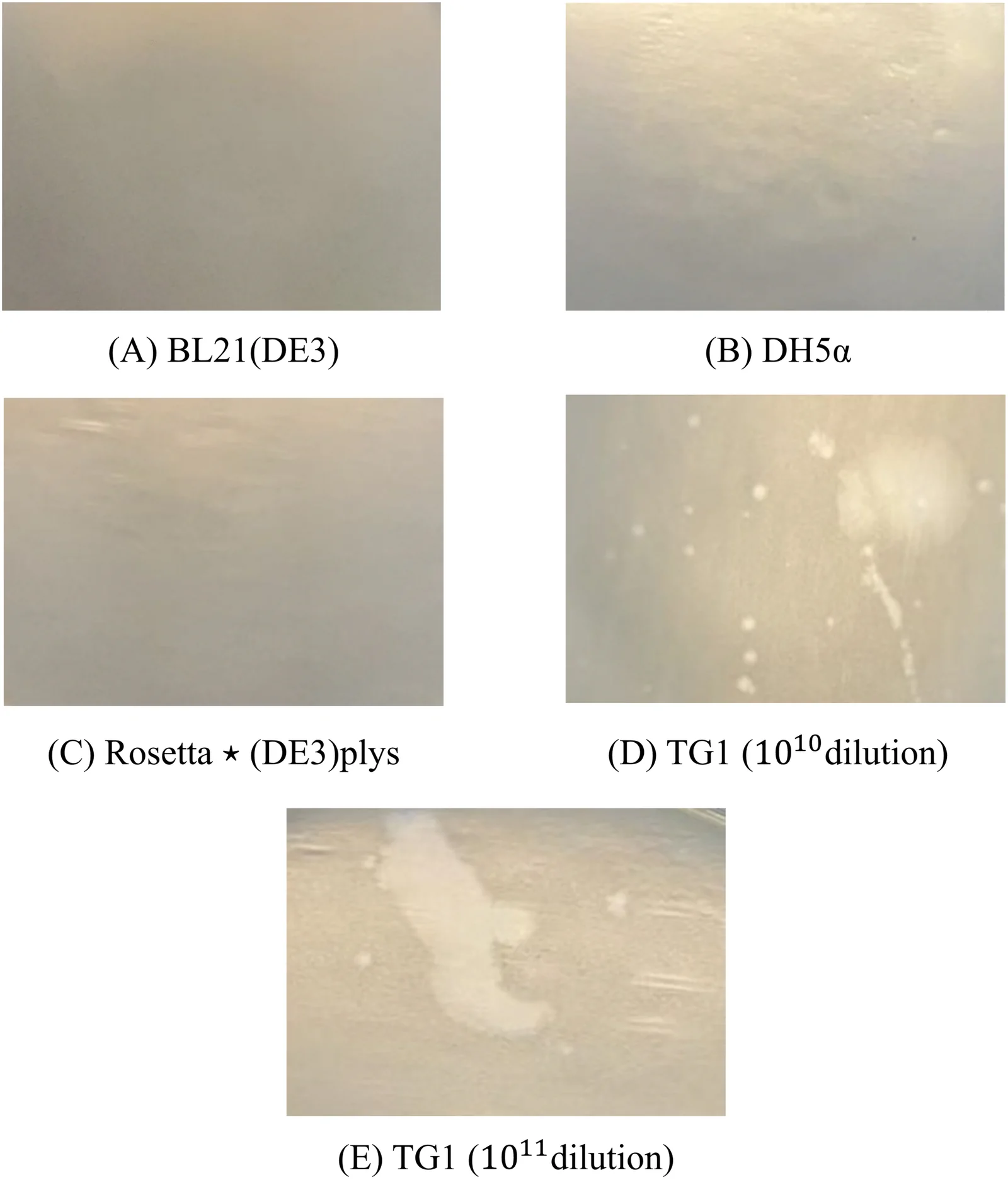
Images of petri dishes of (A)*BL21(DE3)*, (B)DH5α, (C)Rosetta⋆(DE3)plys (D)*TG1*(10^10^ dilution), (E)*TG1*(10^11^ dilution) mixed with phage *M13K07* cultured to logarithmic growth stage.

From Fig 8A-8C, it can be seen that no empty spots appeared in the petri dishes mixed with phage as well as host. In contrast, the *TG1* strain showed significant infectious activity: the number of phage spots was clearly recognizable and stable in the effective counting range (10^10^ versus 10^11^ multiplied dilution gradient). As shown in Fig 8D, the 10^10^ dilution group showed dense but discrete empty spots; Fig 8E showed that the 10^11^ dilution group still maintained a regular distribution of empty spots. Quantitative analysis showed that the measured titers of the two gradients(10^10^ dilution: $7.9\times 10^{13}pfu/mL$, 10^11^ dilution: $8\times 10^{13}pfu/mL$) were highly consistent with the initial phage titer($7\times 10^{13}pfu/mL$), revealing that the unique receptor of the *TG1* strain mediated efficient infection.

Experimental results demonstrating the interaction are presented in Fig 8 and summarized in Table 6. Given that the complete gene sequences of *E.coli* Rosetta⋆(DE3)plys were not retrieved from the NCBI database, they were manually sequenced in this study using the nanopore sequencer MinION. The sequences of the remaining strains were directly adopted from publicly available data provided by NCBI as a way to diversify data sources.

**Table 6:** Predictive performance comparison between FusionPHI and Attn-KP models for *M13K07* phage-host interactions across varied bacterial strains.

Host	FusionPHI				Attn-KP			Label
	*Prob*_0_	*Prob*_1_	Motif	*Result*_1_	*Prob*_0_	*Prob*_1_	*Result*_2_	
*BL21(DE3)*	0.999	0.001	Y	0	1	$2.07\times e^{-15}$	0	0
DH5α	0.336	0.664	Y	1	1	$9.48\times e^{-36}$	0	0
Rosetta⋆(DE3)plys	$1.18\times e^{-4}$	0.999		1	0.984	0.016	0	0
*TG1*	$2.18\times e^{-7}$	1.00	Y	1	0.002	0.998	1	1

In the Table 6, $Prob_{0}/Prob_{1}$ represents the probability of the model predicting the absence or existence of interaction; ’Y’ in the ’Motif’ column indicates the presence of Motif features; *Result*_1_ refers to the prediction result of FusionPHI, and *Result*_2_ refers to the prediction result of Attn-KP. The results show that the predictions of the Attn-KP model for all five groups of phage-host interactions(*Result*_2_ columns) are highly consistent with the experimentally validated labels for these specific strains. In contrast, the prediction of the FusionPHI model was partially biased. The prediction bias of the FusionPHI model in some cases can be attributed to inherent flaws in the raw data.

However, although the DH5α host uses a relatively intact gene, its sequence was generated by manual splicing of multiple segments, and the structural distortion introduced by this preprocessing process also triggers deviations in feature characterization. Splicing boundaries may disrupt the continuity of open natural reading frames, leading MEME to recognize false positive motifs in unnaturally connected regions. When such distorted features were fed into FusionPHI, the model misclassified the artificial splicing noise as a valid biosignal (DH5α, *Prob*_1_ = 0.664), whereas Attn-KP still achieved an accurate classification by virtue of the undisturbed k-mer and physicochemical property patterns.

### Limitations

Despite the promising predictive performance of FusionPHI, several limitations should be acknowledged. First, currently available phage-host interaction datasets remain relatively limited in scale, and the training data used in this study are primarily derived from the PHP dataset, which contains a restricted number of phage-host pairs. Although an independent dataset (PHIAF) was used for external evaluation, the limited data scale may still constrain the generalization capability of the model.

Second, we adopted a balanced 1:1 ratio of positive to negative pairs during training. While this is a common practice for stable model optimization, it does not reflect the biological reality in which true phage-host interactions represent only a tiny fraction of all possible virus-host pairs. As a result, the model may tend to produce overconfident positive predictions when deployed in real-world screening scenarios with severe class imbalance, and its practical utility in such settings remains to be systematically evaluated. In addition, the dataset exhibits an imbalanced taxonomic distribution, where several host genera account for a large proportion of samples while many genera are represented by only a few instances. Such long-tail characteristics may introduce potential taxonomic bias and increase the risk of overfitting toward overrepresented families.

The proposed framework also relies on high-quality and relatively complete genome and protein sequence data. Variations in annotation quality, sequence completeness, or preprocessing procedures may affect downstream feature extraction, particularly for motif identification and protein representation learning. Our case study and ablation experiments further revealed a nuanced dependence: when input genomes are of high quality with complete or near-complete assemblies, the conserved motifs identified by MEME tend to reflect genuine evolutionary signals, and the motif embeddings contribute positively by providing complementary phylogenetic information that enhances prediction. However, when genomes are fragmented, contain assembly errors, or have been subjected to manual splicing that disrupts the natural context of conserved elements, spurious motifs can be introduced at artificial boundaries or within misassembled regions. Such false motif signals may mislead the attention mechanism and introduce noise that degrades prediction accuracy, as observed in the incorrect predictions for the DH5α and Rosetta⋆(DE3)plys strains. This vulnerability, which affects the motif feature that represents a core novelty of our approach, highlights the need for caution when applying FusionPHI to engineered or partially assembled genomes. During dataset construction, a small number of partial genomes (e.g., a 16S rRNA-only record) were retained for rare taxa for which no complete genome could be located; while this was done to avoid taxonomic gaps, it may introduce additional biases and should be considered a limitation of the training data.

Additionally, the translation of conserved nucleotide motifs into amino acid sequences and the subsequent use of ESM-2 protein embeddings, although empirically beneficial in our framework, is not grounded in genuine biological translation. The resulting peptide sequences do not correspond to expressed proteins, and the fixed reading frame may introduce artificial boundaries that deviate from any natural coding sequence. We view this step purely as a pragmatic representation-learning strategy that re-encodes local nucleotide patterns into an amino-acid-level symbolic space, leveraging ESM-2’s capacity to capture general physicochemical properties and contextual dependencies. However, the extent to which these abstract embeddings retain meaningful biological signal beyond what could be obtained from nucleotide-level features alone remains to be rigorously validated. In the absence of direct experimental evidence or systematic controls such as random-frame or frame-shifted baselines, the contribution of these embeddings should be interpreted with caution.

Moreover, we acknowledge a limitation in our five-fold cross-validation setup. The splits were constructed at the sample level without enforcing grouping by phage or host identity, which may allow instances derived from the same biological entity to appear in both training and validation folds. We recognize that this introduces a potential risk of within-validation data leakage. It is important to clarify that cross-validation was employed solely for hyperparameter tuning and model selection; all final performance metrics reported in this study were evaluated on a completely independent test set that was never involved in the cross-validation procedure. Therefore, any leakage that may have occurred within the cross-validation folds cannot have inflated the final reported results, although it could have influenced hyperparameter choices in a suboptimal direction.

Furthermore, the comparative evaluation of baseline methods reported in this study was largely based on performance metrics sourced from a recent comprehensive review (Nie et al. [7]), rather than on a unified re-evaluation. To mitigate this, we independently re‑implemented vHULK, the only baseline compatible with our deep-learning framework, under identical conditions (vHULK‑run in Table 4). For the remaining baselines, re-evaluation was infeasible due to the heterogeneous programming environments and the unavailability of executable code. Their reported values therefore serve as system‑level references, and this limitation should be considered when interpreting the performance gains.

Finally, experimental validation in this study was limited to a single phage-host system (*M13K07*-*Escherichia coli*), which restricts comprehensive assessment of the model across diverse biological systems. Future work will therefore focus on expanding dataset diversity and scale, improving taxonomic balance, evaluating model performance under realistic imbalanced settings, establishing more standardized benchmark comparisons, and conducting broader wet-lab validation to further evaluate the robustness and applicability of the proposed framework.

## Conclusions

This study presents FusionPHI, a novel network model for precise prediction of phage-host interactions based on attention-driven multi-modal feature fusion. By integrating three complementary biological features—k-mer frequency, protein physicochemical properties, and evolutionary conserved motif embeddings—our model addresses certain limitations observed in existing single-modality approaches on our evaluated datasets. The dual-stage attention mechanism, including self-attention followed by cross-attention, enables synergistic optimization of intra-modal feature refinement and cross-modal biological correlation mining.

Computational evaluation demonstrates that FusionPHI achieves competitive benchmark performance with 91% ROC AUC in cross-validation, showing certain accuracy improvements over the specific baseline methods evaluated. The ablation study quantitatively confirms the indispensability of each component: 1) Motif embeddings provide critical discriminative power for class boundary delineation despite their high-dimensional sparsity; 2) Self-attention enhances context-aware representation within modalities; 3) Cross-attention enables dynamic biological correlation mapping across modalities.

Beyond computational metrics, wet lab validation through phage plaque assays confirms FusionPHI’s biological relevance. Case studies further demonstrate its potential to identify strain-specific infection patterns with biological relevance.

Future work will focus on extending the framework to broader phage systems, integrating additional structural and functional biological features, and developing user-accessible prediction platforms for practical applications.
