# Supplementary material for: FusionPHI: A phage-host interaction prediction network model based on attention-driven multi-modal feature fusion
Source: PLoS One. 2026 Aug 21;21(8):e0356238. doi: 10.1371/journal.pone.0356238 (PMC13497294; doi:10.1371/journal.pone.0356238)
Supplement: Supplementary file 1 [file pone.0356238.s002.docx]

**Response to Comments**

We thank the editor and reviewers for their constructive comments. We have carefully followed their suggestions and addressed the raised issues. Detailed responses to the comments are as follows. Major revisions are marked in **RED** in the revised manuscript.

A. **Responses to Reviewer 1:**

**General** **Comment:** This manuscript presents FusionPHI, a deep learning model for phage–host interaction (PHI) prediction based on multi-modal feature fusion: genomic k-mer frequencies, protein physicochemical properties, and motif-level embeddings derived from ESM-2, integrated via a dual-stage attention-driven feature fusion module. The authors benchmark FusionPHI against several existing PHI tools and include both ablation studies and a small experimental case study with M13K07 and several E. coli strains.

The topic is timely and clearly within the scope of PLOS ONE. The study appears to report original research and the approach is promising. However, there are important issues regarding methodological clarity, data availability, and explanation of the model to a biological audience that, in my view, require substantial revision.

First, the strengths of the manuscript:

Phage–host prediction remains a key bottleneck for interpreting metagenomic data and for phage therapy applications. A model that integrates multiple sequence-derived modalities is highly relevant to the community. The authors provide a good introduction that is consistent with the current state of knowledge, both regarding the challenges of phage therapy and the relevant bioinformatics problems.

The idea of combining: genomic k-mer features, aggregated protein physicochemical descriptors, and motif-level embeddings derived from a large protein language model (ESM-2) is conceptually appealing and could potentially capture complementary aspects of phage–host compatibility. New algorithms addressing the phage–host matching problem are very important.

The dual-stage self-/cross-attention feature fusion module is an interesting design choice for weighting and integrating heterogeneous feature types. The inclusion of ablation experiments to dissect the contribution of different feature sets and attention blocks is a clear strength.

The experimental infection assays with M13K07 and several E. coli strains, although limited in scope, are a welcome attempt to connect computational predictions with real biological behavior.

The authors provide a GitHub repository with the implementation of FusionPHI, which is an important step towards reproducibility.

**Response:** We thank the reviewer for the careful reading and for the positive overall assessment of our work. We are encouraged that the reviewer finds the topic timely and relevant, and that the design of FusionPHI—particularly the integration of multi-modal sequence-derived features and the attention-based fusion strategy—is considered meaningful. We also appreciate the recognition of our ablation analyses, preliminary experimental validation, and the public release of the code. We will carefully address the points raised in the following revisions.

**Comment 1:** The manuscript repeatedly states that motif embedding vectors constitute an “innovative feature”:

“In particular, we introduce an innovative feature, that is, the embedding vectors of the gene motif sequence.”

However, the novelty is not clearly defined or justified.

Protein language models (including ESM-2) have already been used to compute embeddings for protein sequences and sometimes for protein fragments/domains.

It is not clear in what precise sense motif-level embeddings are conceptually new compared with: embedding entire ORFs or full protein sequences, or using other fragment-based embeddings.

Please more clearly position FusionPHI relative to other recent multi-modal or attention-based PHI methods: which components are conceptually new (e.g. the dual-stage cross-attention design, use of motif-level ESM-2 embeddings), and which are extensions of earlier work?

**Response:** Thanks for your insightful comment. We completely agree that the submitted manuscript did not sufficiently articulate the precise novelty of our motif-level embeddings and the clear positioning of FusionPHI relative to existing methods. We have comprehensively addressed these points in the revised manuscript and detailed them below.

**Regarding the specific novelty of motif-level embeddings:** While ESM-2 has indeed been widely utilized to compute embeddings for full ORFs or entire protein sequences, these global representations often dilute the specific, localized signals that dictate phage-host interactions. On the other hand, embedding arbitrary sequence fragments lacks defined biological relevance. The conceptual novelty of our approach lies in specifically isolating functional motifs—which represent highly conserved, biologically significant interaction units—and encoding them as an independent modality. Unlike full-sequence embeddings that contain noise from non-interacting regions, or fragment embeddings that lack functional context, our motif-level embeddings force the language model to capture the concentrated semantic and structural features of the actual binding interfaces.

**Regarding the positioning of FusionPHI (Innovation vs. Extension):** To provide a transparent comparison with other recent multi-modal or attention-based PHI methods, we have explicitly delineated our novel contributions from the components that extend prior work:

Extensions of earlier work: The utilization of ESM-2 as a foundational feature extractor, the general concept of integrating multi-modal data for PHI prediction, and the baseline application of attention mechanisms represent extensions of established methodologies in the field.

**Conceptual and Methodological Innovations:** The primary innovations of FusionPHI are twofold. First is the conceptual introduction of motif-level ESM-2 embeddings as a distinct, specialized modality, which to our knowledge is the first application of its kind in PHI prediction. Second is the methodological design of the dual-stage self-/cross-attention fusion architecture. This specific mechanism is innovatively tailored to effectively align and integrate the highly localized, functionally dense motif signals with broader global sequence features, overcoming the limitations of standard attention modules that struggle to balance local functional units with global contexts.

We have incorporated these detailed clarifications into the Introduction section of the revised manuscript to ensure a transparent positioning of our work. We appreciate your rigorous feedback, which has significantly strengthened the theoretical foundation of our paper.

**Comment 2:** The text mentions that “27 original features” per sample are expanded to 81-dimensional vectors, but it is not initially clear what these 27 features are. From the Methods, one can infer that they correspond to:

7 physicochemical indices (length, pI, molecular weight, aromaticity, instability index, flexibility, GRAVY), plus

20 amino acid composition frequencies.

I suggest explicitly stating this in the text before introducing the expansion to 81 dimensions, so that the origin of the “27 features” is transparent.

**Response:** Thanks for pointing out this lack of clarity. In the **“Physicochemical properties based proteomic feature encoding”** subsection of the revised manuscript, we have explicitly defined the “27 original features” at their first occurrence, clearly stating that they consist of 7 physicochemical indices (sequence length, isoelectric point, molecular weight, aromaticity, instability index, flexibility, and GRAVY) together with 20 amino acid composition frequencies. We have also clarified the subsequent feature transformation process, explaining how these 27 features are expanded into an 81-dimensional representation. These revisions are intended to make the origin and construction of the feature vector fully transparent to the reader.

**Comment 3:** The model uses k = 4 for genomic k-mer frequencies, but the choice of this value is not justified.

Were other k values (e.g. 3, 5, or combinations) tested?

Is k = 4 chosen based on previous work on the same dataset, or on a systematic performance comparison?

Given that k-mer choice can substantially affect performance and feature dimensionality, please either:

justify k = 4 with references and/or include a brief sensitivity analysis showing that k = 4 is a reasonable choice.

**Response:** We thank the reviewer for this important comment. In the **“K-mer frequency based genomic feature encoding”** subsection of the revised manuscript, we have clarified the rationale for selecting k=4 by incorporating relevant references from prior studies, where this choice has been empirically validated as a balanced setting for capturing informative sequence patterns while controlling feature dimensionality in the existing PHI work like PHP and HostPhinder. We have revised the text to explicitly state that our selection is consistent with established practice in related genomic and PHI prediction studies. These additions are intended to provide a clearer justification for the use of k=4 in our model.

**Comment 4:** The manuscript states:“Subsequently, in order to more accurately capture the protein functional properties of these conserved regions, we performed transcription and translation operations on each motif mk to generate its corresponding RNA sequence rk and protein sequence pk, respectively.”

If motifs are translated without strict alignment to the original ORF frame, ESM-2 may be embedding artificial peptide sequences, which could compromise the biological meaning of the features.

Please clarify the exact procedure: how motifs are mapped to ORFs, how the reading frame is determined, and how non-frame or non-multiples-of-three motifs are handled (filtered, trimmed, or still translated).

**Response:** Thanks for this important and technically precise question. We agree that the submitted manuscript did not sufficiently describe how motif sequences are handled during the transcription and translation steps.

First, regarding the mapping between motifs and ORFs, we clarify that in our current framework motifs are not explicitly mapped back to annotated ORFs, nor are they constrained to lie within a known coding frame. Instead, motifs identified by the MEME suite are treated as conserved nucleotide patterns at the genome level, independent of gene annotation. Our goal is not to reconstruct native coding sequences, but to extract evolutionarily conserved local signals and project them into amino acid space for downstream representation learning.

Second, concerning the reading frame, we do not attempt to infer or enforce a biologically “correct” reading frame for each motif. Rather, a fixed and consistent translation rule is applied to all motifs. This design reflects the fact that motif boundaries are not guaranteed to align with codon structure, and enforcing a frame could introduce additional uncertainty or bias. We have revised the manuscript to explicitly state that the translation step is used as a feature transformation rather than a reconstruction of true protein sequences.

Third, for motifs whose lengths are not multiples of three, we clarify that no filtering or trimming is applied. Instead, motifs are directly translated under the same rule, allowing partial codons at the boundaries to be handled consistently (i.e., truncated during translation if necessary). This ensures that all identified motifs contribute to the representation, while maintaining a uniform processing pipeline.

We have added a detailed description of these points in the revised **“Gene motif based genomic feature encoding”** subsection and clarified the wording to avoid any implication of strict ORF-aware translation. We also explicitly state that the resulting amino acid sequences should be interpreted as abstracted representations of conserved sequence patterns, rather than biologically expressed peptides.

**Comment 5:** The dual-stage attention-driven fusion module (self-attention followed by two cross-attention blocks) is central to FusionPHI. The current description is mathematically correct but quite difficult to follow for readers without a deep learning background.

I recommend adding a brief, intuitive explanation, for example:

what self-attention is expected to capture within each modality (e.g. which k-mers or motif features are most informative), what cross-attention is expected to capture between modalities (e.g. aligning specific motif patterns with particular genomic patterns or protein properties), how the two stages complement each other.

A small schematic or a simple toy example would substantially improve accessibility for biologists and bioinformaticians not specializing in ML.

**Response:** Thanks for this helpful suggestion. We agree that the original description of the dual-stage attention module was relatively difficult to follow for readers without a deep learning background, despite being mathematically correct.

In the **“Dual-stage attention-driven feature fusion”** subsection of the revised manuscript, we have added an intuitive explanation of the attention mechanism using a Query–Key–Value analogy (similar to a database retrieval process). This is intended to provide a conceptual understanding of how the model dynamically assigns importance to different features, making the underlying idea more accessible to non-specialists.

We now explicitly clarify the role of self-attention in our framework. Specifically, self-attention operates within each modality to capture intra-modality dependencies, such as identifying which k-mer patterns, physicochemical properties, or motif-derived features are most informative, while down-weighting less relevant or noisy signals.

We further expand the explanation of cross-attention by emphasizing its role in modeling inter-modality relationships. In particular, we describe how the model aligns global genomic and proteomic features with conserved motif patterns, enabling the integration of complementary biological signals across modalities.

Finally, we clarify how the two stages complement each other: self-attention refines modality-specific representations, while cross-attention bridges these representations to form a unified and biologically meaningful feature space. To further improve accessibility, we have incorporated schematic figures and a simplified explanatory example in the revised manuscript, which together aim to make the framework more interpretable for a broader biological and bioinformatics audience.

**Comment 6:** The manuscript states that ORF sequences or gene sequences are used, but it is not sufficiently clear how these were obtained:

Were ORFs taken from existing annotations in NCBI/RefSeq or from the PHP dataset?

Were they predicted de novo (e.g. using NCBI ORFfinder, Prodigal, or another tool)? If so, with which parameters?

**Response:** Thanks for your insightful comments regarding data provenance and ORF acquisition, and we agree that the submitted manuscript did not describe this aspect with sufficient clarity.

In our study, we did not directly use the sequence data provided within the original PHP dataset. Instead, we used the taxonomic information and accession IDs from that dataset to retrieve complete and standardized genomic sequences from the NCBI RefSeq database. This ensures consistency and data quality across all samples.

Regarding ORFs, we did not perform de novo prediction using tools such as ORFfinder or Prodigal. Rather, all ORF sequences and their corresponding protein translations were directly obtained from the official annotated records in RefSeq (e.g., associated .faa files). This design choice ensures that the protein sequences used for downstream feature extraction are based on curated and biologically validated annotations, rather than potentially noisy computational predictions.

To address the reviewer’s concern and improve transparency, we have added a detailed description of the data sources and retrieval procedures in the “**Datasets”** subsection of the revised manuscript, so that readers can clearly understand how genomic and ORF-level data were obtained and processed.

**Comment 7:** In the case study discussion, the authors note that for the BL21⋆(DE3)pLysS host, they used an “UNVERIFIED, partial pfkB gene sequence” from NCBI, and they attribute a misprediction to this low-quality, truncated sequence. This raises two concerns:

It contradicts the earlier impression that complete genomes or curated ORF sets are consistently used to represent hosts.

It is unclear why such a partial, unverified gene sequence was used at all, rather than a complete genome assembly or curated annotation.

Please: clarify, for all hosts, whether full genomes or partial genes were used, and using what pipeline; justify the inclusion of partial, unverified sequences or, preferably, exclude them and treat such issues as dataset limitations rather than simply attributing errors to the model.

**Response:** Thanks for highlighting this inconsistency and providing such constructive guidance. We fully agree that including an unverified, partial gene sequence compromises the rigor of our evaluation and contradicts our primary methodology. Following your preferred recommendation, we have completely excluded the BL21⋆(DE3)pLysS host data from Table 4 and removed the associated narrative from the case study discussion.

To address your concern regarding the overall data quality, we wish to clarify that for all other hosts evaluated in this study, we strictly utilized complete genome assemblies and curated ORF sets obtained through the standard NCBI RefSeq annotation pipeline. Furthermore, to address the broader concern of data quality versus model attribution, we have added a new subsection titled **“Limitations”** in the revised manuscript. By framing these issues as dataset limitations rather than intrinsic model failures, we provide a more objective evaluation of the current technological boundaries in phage-host interaction prediction.

**Comment 8:** The dataset description is quite brief and leaves several important questions open:

Negative pair construction:

How were non-interacting phage–host pairs generated from the PHP resource? Random pairing? Any biological or taxonomic constraints?

Taxonomic distribution:

What is the distribution of host species and phage species/families? For example, how many hosts belong to Enterobacteriaceae or other dominant families?

Train/validation/test splitting:

Are folds split at the pair, phage, or host level? Do the same phage or host genomes appear in both training and test sets? Without careful splitting (e.g. host- or phage-level segregation), there is a risk of information leakage and overestimation of performance.

Whether any precautions were taken to prevent information leakage (e.g. the same genome contributing to both training and test)?

These details are critical for interpreting the reported AUC and for judging generalizability.

It is not fully clear on which dataset(s) and at which taxonomic level these numbers are obtained, and whether all tools are evaluated under the same conditions (same input data, same host taxonomy level, same negative sampling strategy). Is this tool suggested to solve PHI problem on genus/species/strain level? The 1 case study is not enough in the opinion of this reviewer.

Please provide:

a more detailed description of dataset construction, including how many phages and hosts per family, a clear explanation of how negative pairs are defined, and an explicit statement of the splitting strategy and any stratification by taxonomy.

**Response:** Thank you for raising this important concern regarding potential information leakage between the training and testing datasets.

In our study, the PHP dataset was used for training and cross-validation, while the PHIAF dataset served as an independent test set. To assess possible overlap, we performed a systematic comparison between the two datasets.

The PHP dataset contains 336 unique phage genomes, whereas the PHIAF dataset includes 304 unique phage genomes. Among them, 18 phage genomes appear in both datasets (approximately 5.36% of PHP and 5.92% of PHIAF). Importantly, we verified that no identical phage–host interaction pairs exist across the two datasets.

Additionally, the host representations differ between the datasets. The PHP dataset records hosts at the species level, while the PHIAF dataset uses genome accession identifiers. Consequently, even when the same phage genome appears in both datasets, the associated host entries are different, preventing identical interaction pairs from occurring.

These results indicate that direct pair-level information leakage does not occur between the training and testing sets. We have added a detailed description of this dataset comparison in the revised manuscript and included the list of overlapping phage genomes in Table 1.

**Comment 9:** The Data Availability statement and the GitHub repository suggest that code and data are available, but on inspecting the repository one finds only example training samples, not the full datasets used for the analyses.

**Response:** We sincerely apologize for this oversight and express our gratitude to the reviewer for their meticulous inspection of our repository. We have now fully rectified this issue by uploading the complete datasets used for all analyses in this study to the GitHub repository.

To clarify, during the initial preparation of the repository, we uploaded a lightweight subset of example data specifically to allow users to quickly test and verify the execution of our code pipeline. However, due to a genuine oversight on our part, we neglected to upload the complete datasets prior to the final manuscript submission. We greatly appreciate your diligence in catching this error. The repository has now been fully updated with all the required data, ensuring that researchers can seamlessly access the complete datasets and independently reproduce our entire analytical framework.

**Comment 10:** The M13K07 / E. coli case study is a valuable addition, but several points remain unclear:

Strain selection:

Why were BL21⋆(DE3)pLysS, BL21(DE3), DH5α, Rosetta⋆(DE3)pLysS, and TG1 chosen specifically? Are these strains known or suspected to be non-permissive for M13/M13K07, or is this being tested for the first time? If relevant literature exists, please cite it.

Relationship to the training set:

How strongly are Enterobacteriaceae, and especially E. coli, represented in the training dataset? Is there a risk that the model is biased or overfitted toward certain families or strains?

How other tools used in the benchmark predict the outcome of the experiment?

Stratification between train and case study:

Were closely related E. coli strains (or the same strains) present in the training data? If so, please discuss how this affects the interpretation of the case study as an “independent” validation.

Sequence quality issue (BL21 partial pfkB):

As mentioned above, the use of a partial, unverified gene sequence to represent the host is problematic. At minimum, this should be clearly acknowledged as a limitation of the dataset and not just a post hoc explanation for a misprediction; ideally, low-quality entries should be filtered out and the analysis updated.

Clarifying these aspects would make the case study more convincing and biologically interpretable.

**Response:** We sincerely thank the reviewer for finding our case study valuable and for raising these insightful questions. We fully agree that clarifying the biological rationale, the dataset stratification, and the specific scope of this experiment significantly enhances the interpretability and rigor of this study. We have carefully addressed your concerns and revised the manuscript accordingly.

Regarding the rationale for strain selection, while these specific E. coli strains are widely available laboratory models, they were deliberately chosen for this case study because they represent distinct genetic backgrounds with varying known or expected permissivity to M13-derived phages (such as M13K07). It is well established in the literature that M13 infection strictly requires the presence of the F pilus on the host cell surface. Among our selected strains, TG1 carries an F' episome, making it a classic permissive host for M13 propagation and phage display. Conversely, strains like DH5α, BL21(DE3), and their derivatives typically lack the F factor, rendering them naturally non-permissive to M13 infection. Selecting this specific mix of known permissive (TG1) and non-permissive hosts provides a biologically rigorous setup—serving as built-in positive and negative controls. This design allows us to evaluate whether the model can discern subtle, sub-species (strain-level) genomic differences that dictate actual interaction outcomes. We have explicitly added this biological justification and relevant citations to the case study methodology in the revised manuscript.

We also appreciate the reviewer’s vigilance regarding potential data leakage and overfitting. To address this, we systematically analyzed the distribution of E. coli in our training data (derived from the PHP dataset). Statistically, among the 82 host bacterial species included, E. coli accounts for only 3.7% of the species distribution, and the genus Escherichia is represented in merely 25 phage-host interaction (PHI) pairs in the training set. This relatively low representation provides strong evidence that our model is not biased or overfitted toward the Enterobacteriaceae family or E. coli strains.

Regarding stratification, while the species E. coli exists in the training data, the specific engineered strain variants used in our case study (e.g., specific BL21 variants with expression plasmids, TG1), as well as the engineered helper phage M13K07, were completely absent from the training set. The model had not "seen" these specific strain-level genomic profiles or this specific phage prior to the test. Therefore, predicting their specific interactions requires true generalization based on learned biological feature rather than mere memorization of the training data, preserving the integrity of this independent validation.

In light of your question regarding how other benchmarked tools might predict the outcome of this specific experiment, we would like to clarify the distinct positioning and objective of this case study within our paper. The comprehensive performance comparison between our method and other state-of-the-art tools has been rigorously established in the main benchmarking section using standardized, large-scale computational datasets. In contrast, the primary objective of the M13K07 / E. coli case study was to construct a logical closed loop from "computational theory to biological reality." By conducting this wet-lab validation, our goal was strictly to demonstrate that our model's in silico predictions can be reliably translated into accurate outcomes in actual biological experiments and real-world samples. Because the core intent of this specific section is to validate empirical applicability rather than to conduct an additional comparative algorithmic benchmark, extending this specific wet-lab validation to other tools falls outside the intended scope of this case study. We have refined the introduction of the case study section to better articulate this specific objective.

Finally, regarding the sequence quality issue (BL21 partial pfkB), as extensively discussed in our response to Comment 7, we fully agree with the reviewer that utilizing unverified, partial gene sequences is inappropriate and problematic. To strictly maintain the rigor of our analysis, we have completely excluded the problematic BL21⋆(DE3)pLysS data from the case study, removed its associated narrative, and updated the analysis accordingly. Furthermore, we have introduced a dedicated "Limitations" subsection to clearly acknowledge the impact of sequence completeness and quality on prediction accuracy, ensuring this is treated as an inherent data limitation rather than a post hoc explanation.

**Comment 11:** Many of the limitations of the study are implicit (dataset size, quality of annotations, single experimental system, possible taxonomic bias), but there is no dedicated paragraph or section that clearly summarizes them.

I strongly recommend adding a short “Limitations” subsection in the Discussion, explicitly covering:

limited dataset size, potential taxonomic biases and possibility of overfitting to overrepresented families, dependence on high-quality and complete host and phage sequences, the narrow scope of experimental validation (one phage–host system). This will strengthen the balance and transparency of the manuscript.

**Response:** We highly appreciate this constructive and insightful recommendation. We completely agree that centralizing these points significantly strengthens the balance, transparency, and overall scientific rigor of our manuscript. In accordance with your suggestion (and aligning with our previous response to Comment 7), we have added a dedicated “Limitations” subsection at the end of the Discussion. Specifically, this new section explicitly acknowledges that our model's generalization and performance may be constrained by the limited scale and imbalanced taxonomic distribution of current training datasets, alongside an inherent reliance on high-quality, complete sequence annotations. Furthermore, we transparently address the narrow scope of our current wet-lab validation, which is currently limited to a single phage-host system, thereby highlighting the necessity for broader experimental evaluations in our future work.

**Comment 12:** Figure(s) showing plaque assays are of relatively low resolution in the PDF, and plaques are difficult to see clearly.

**Response:** We sincerely apologize for the suboptimal visual quality of the plaque assay figures in the manuscript, and we completely understand the reviewer's concern.

To clarify this visual limitation, we would like to explain the inherent biological characteristics of our assay. The phage utilized in our experiments is a **non-lytic phage**. Unlike strictly virulent phages that completely lyse host cells to form highly transparent and distinct clearings, this phage does not cause host lysis upon infection. Instead, it establishes an infection cycle that merely slows down the growth rate of the infected host cells. This specific biological mechanism naturally results in the formation of turbid and visually subtle plaques, which is the primary reason they appear less pronounced and are inherently difficult to capture with high optical clarity.

Furthermore, the original photographic records of these specific assay plates were captured using a mobile device camera under standard laboratory conditions at that time, which inherently restricted the maximum optical resolution. Furthermore, as biological plaque assays are highly sensitive to transient environmental conditions, batch-specific biological sample states, and specific temporal dynamics, attempting to replicate this exact assay solely for photographic purposes would inevitably introduce biological variations. Consequently, we are unable to faithfully recreate the exact experimental snapshot presented in our original results.

To mitigate this issue and provide the clearest possible view, we have carefully adjusted the image contrast of the original photographs to make these biologically subtle plaques more distinguishable. We hope that this biological context, alongside the digital contrast enhancements, provides a satisfactory resolution to your concern.

**Comment 13:** Phrases such as “overcomes fundamental limitations in existing single-modality approaches” and very strong “state-of-the-art” claims may be slightly overstated given the dataset size and limited experimental validation. I suggest softening such statements and clearly separating benchmark performance from broader biological generalization.

**Response:** We sincerely thank the reviewer for this insightful and highly constructive comment. We entirely agree that our original phrasing, including terms like "state-of-the-art" and statements claiming to "overcome fundamental limitations," was overstated given the current dataset size and the limited scope of our wet-lab experimental validation. In response to your valuable feedback, we have thoroughly reviewed the entire manuscript and carefully softened these claims to accurately reflect the scope of our findings. Specifically, we have replaced absolute terminology with more objective descriptions, characterizing our model as achieving "competitive benchmark performance" and stating that it "addresses certain limitations observed in existing single-modality approaches on our evaluated datasets." Furthermore, we have comprehensively revised the Abstract, Results, and Conclusion sections to ensure a rigorous and clear distinction between our model's computational performance on the benchmark datasets and its broader biological generalization capabilities. We believe these revisions provide a much more objective, scientifically rigorous, and precise representation of our current work, and we appreciate your guidance in helping us improve the quality of this manuscript.

B. **Responses to Reviewer 2:**

**General Comment:** The manuscript presents an interesting approach to motif-based sequence analysis and proposes a novel model. While the topic is relevant and potentially suitable for PLOS ONE, the current version of the manuscript requires substantial revision to meet the journal’s standards of methodological clarity, reproducibility, and rigor.

Response: We sincerely thank the Editor and the Reviewer for dedicating their time to evaluating our manuscript and for providing such comprehensive and constructive feedback. We are greatly encouraged by your positive recognition of our motif-based sequence analysis approach, the novelty of our proposed model, and its potential relevance to the readership of PLOS ONE.

We fully acknowledge and respect your concerns regarding the methodological clarity, reproducibility, and scientific rigor of the initial submission. We have taken your evaluations very seriously and have undertaken a substantial and meticulous revision of the manuscript to strictly align with the high standards of the journal. To directly address your core concerns: First, to guarantee reproducibility, we have fully updated our GitHub repository to include the complete, full-scale datasets used in our analyses, rectifying the previous omission. Second, to enhance scientific rigor, we have rigorously cleaned our evaluation data by entirely excluding unverified, partial sequences, and we have introduced a dedicated “Limitations” section to transparently discuss data constraints and taxonomic boundaries. Finally, to improve methodological clarity, we have extensively expanded the rationale and biological context of our case study design, explicitly detailing strain selection criteria and dataset stratification.

We firmly believe these comprehensive revisions have fundamentally strengthened the transparency, rigor, and overall quality of our study. Please find our detailed, point-by-point responses to your specific comments below.

**Comment 1:**  The authors claim that their model outperforms existing approaches; however, this conclusion is not sufficiently supported by the presented results. In Fig. 3, the SVM baseline appears to perform comparably, and potentially more consistently, than the proposed method.

Additionally, the use of a boxplot with only five data points is potentially misleading. A swarmplot or an alternative visualization would provide a clearer representation of the data distribution. Regardless of visualization, the reported differences between the proposed model and SVM do not appear to be statistically significant, which weakens the central claim of superiority.

**Response:** Thanks for your insightful comments. We have revised both the visualization and the interpretation accordingly.

First, we agree that a boxplot with only five observations per model is not ideal. We have replaced it with a stripplot (with jitter) to ensure that all fold-level results are fully visible, providing a clearer representation of performance distribution and variability.

Second, we acknowledge that SVM shows competitive and stable performance. We have therefore moderated our claims and now describe our method as achieving comparable or slightly improved performance rather than clear superiority. We also clarified in the manuscript that the observed differences are not statistically significant.

Finally, we emphasize that the main contribution of this work lies in the integration of sequence and structural features, as well as improved interpretability, rather than marginal performance gains alone.

These revisions have been reflected in the updated manuscript.

**Comment 2:** The manuscript does not clearly describe the feature sets used to train the baseline models. This makes it difficult to assess whether the comparison is fair.

Furthermore, the authors state that their model relies less on hand-crafted features. However, this claim is questionable, as the model input consists of preprocessed representations rather than raw DNA or protein sequences. The preprocessing pipeline itself effectively constitutes feature engineering and should be described and discussed accordingly.

**Response:** We sincerely thank the reviewer for raising these critical points regarding the baseline comparisons and our claims about feature engineering. We fully agree with both assessments and have made the corresponding revisions to ensure rigorous and accurate reporting.

Regarding the feature sets of the baseline models, we agree that this clarification is crucial for a rigorous evaluation. The performance metrics for the baseline methods were directly adopted from the standardized evaluations in Nie's review[Nie, W., Qiu, T., Wei, Y., Ding, H., Guo, Z., & Qiu, J. (2024). Advances in phage–host interaction prediction: in silico method enhances the development of phage therapies. *Briefings in Bioinformatics*, *25*(3), bbae117.], rather than through our own re-implementation. The primary reason for this is that classical models (such as VHM and PHP) are algorithmically designed for specific single-modal features (e.g., alignment-free k-mer frequencies) and cannot intrinsically process multi-modal inputs like protein physicochemical properties or ESM-2 embeddings. Modifying them to accept our unified feature set would fundamentally alter their core mechanisms. Therefore, the comparison evaluates FusionPHI as a complete, end-to-end prediction system against existing tools, rather than comparing architectures under identical feature constraints. Furthermore, to address the concern of whether the performance gain stems merely from using more features, our Ablation Study demonstrates that our superior performance relies heavily on the synergistic capability of the dual-stage attention architecture to effectively fuse these distinct modalities, not just on the raw presence of more data. To make this strictly clear to readers, we have added a clarifying paragraph in the revised manuscript within the "Comparison with existing benchmark methods" subsection to explicitly discuss the rationale and rigor behind this end-to-end comparison strategy.

Furthermore, we deeply appreciate the reviewer for pointing out the logical inconsistency regarding our feature engineering claims. Our previous statement claiming that the model **"transcends the constraints of manual feature engineering"** was indeed inaccurate. We acknowledge that the comprehensive preprocessing pipeline we developed, which includes the sliding-window k-mer frequency calculations, the extraction of protein physicochemical properties, and the generation of ESM-2 motif embeddings, does effectively constitute a systematic and rigorous feature engineering process. We have corrected this narrative in **“Comparison with existing benchmark methods”**subsection of the revised manuscript. Specifically, we removed the misleading phrasing regarding the limitations of manual feature engineering. Instead, we have revised the corresponding paragraph to explicitly acknowledge that our model's success is built upon this carefully designed, multi-modal feature engineering pipeline, which extracts dense and highly informative biological representations prior to feeding them into the attention-driven fusion network.

**Comment 3:**   The preprocessing approach raises several important questions. Specifically, it is unclear why MEME was executed with DNA-specific parameters to identify motifs that appear to be protein-related.

This configuration is typically more appropriate for identifying transcription factor binding motifs. Moreover, the subsequent conversion of identified motifs into RNA and protein sequences is not sufficiently justified and does not appear to add clear biological meaning.

The authors should provide a detailed and biologically grounded rationale for this workflow.

**Response:** We highly appreciate the reviewer’s rigorous assessment of our preprocessing pipeline. We agree that from a strict molecular biology perspective, directly converting DNA motifs into amino acid sequences using a fixed-frame translation is an unconventional approach, as genomic DNA motifs may encompass non-coding regulatory regions (such as promoters or binding sites). To clarify our rationale and resolve any ambiguity, we have extensively revised both the "Datasets" and "Gene motif based genomic feature encoding" sections in the manuscript.

Firstly, we must clarify that our model utilizes two distinct pipelines for feature representation. For the extraction of global physicochemical properties, we strictly utilized officially curated Open Reading Frame (ORF) amino acid sequences downloaded directly from NCBI (avoiding any de novo computational prediction), ensuring high biological validity. This has now been explicitly detailed in the newly added "Datasets" subsection.

Secondly, regarding the MEME motif workflow, our objective was entirely feature-driven rather than aiming to reconstruct functionally complete proteins. We executed MEME at the DNA level specifically to capture universal genomic signatures across the entire viral and host genomes, which inherently include critical regulatory elements governing phage-host interactions. The subsequent fixed-frame translation of these DNA motifs into the amino acid space acts as a computational heuristic—a tokenization strategy—to bridge these raw genomic sequence patterns with the ESM-2 protein language model. While this process does not yield biologically functional proteins, mapping the nucleotide patterns into a high-dimensional amino acid vocabulary allows us to leverage ESM-2’s exceptional capacity to capture long-range sequence dependencies and co-evolutionary context. Empirically, embedding these sequence signatures into the ESM-2 space provided a more robust and discriminative representation for our predictive task than utilizing raw nucleotide k-mers or DNA-specific models. We have thoroughly integrated this computational rationale into the revised methodology section.

**Comment 4:**  There are concerns regarding the evaluation procedure. In Fig. 3, some methods exhibit ROC-AUC values significantly below 0.5. This suggests that the models may be learning an inverse relationship (i.e., systematically predicting the opposite class), which requires explanation.

The manuscript would benefit from:

1)A clearer description of dataset construction

2)An explicit discussion of train/test splits

3) An analysis of sequence similarity between training and test sets

Without this information, it is difficult to rule out potential data leakage or an unfair advantage for models that can memorize training data.

**Response:** We sincerely thank the reviewer for this rigorous and insightful evaluation of our experimental design. We fully agree that a transparent description of the datasets and a robust analysis of potential data leakage are critical for ensuring a fair evaluation. We have extensively revised the manuscript to address all your concerns.

Regarding the specific concern about ROC-AUC values falling below 0.5 for certain benchmark methods in Fig. 3(Original, Now is Fig. 6), we have investigated this phenomenon and added a clarification in the Results section. In machine learning, an AUC significantly below 0.5 on an independent test set typically indicates a severe distribution shift coupled with overfitting. These specific baseline methods heavily overfitted to the distinct taxonomic distributions and genomic signatures of their original training environments. Consequently, when evaluated on our independent cross-dataset benchmark—which exhibits a highly heterogeneous and long-tail taxonomic distribution—their learned decision boundaries were completely misaligned with the novel genomic contexts. Instead of learning universal rules of phage-host interactions, they memorized specific training data features, leading to systematic misclassifications on unseen taxa rather than mere random guessing.

To comprehensively address your recommendations regarding dataset transparency and data leakage, we have introduced a dedicated **“Datasets”** subsection within the Materials and Methods section. In this new subsection, we explicitly detail the construction process and the train/test splits. Our training data utilizes the PHP dataset (comprising 672 pairs, 82 host species, and 333 phages), while our independent test set utilizes the PHIAF dataset (comprising 235 host species and 304 phages). To enhance clarity, we have added new visualizations (Fig. 1 and Fig. 2) that explicitly illustrate the long-tail distribution and taxonomic proportions of the training data.

Most importantly, to explicitly rule out data leakage and sequence similarity bias, we conducted a rigorous cross-dataset overlap analysis, the results of which are now presented in Table 1. While 18 phage genomes are shared between the datasets, we confirmed that there are zero identical phage-host interaction pairs across the training and testing sets. Furthermore, because host instances differ, the biological contexts of the interactions remain entirely distinct. This rigorous stratification guarantees that no pair-level information leakage occurred. Therefore, the models cannot gain an unfair advantage through simple memorization, ensuring that our evaluation effectively and fairly measures true generalization capabilities.

**Comment 5:** The ablation study is not sufficiently informative in its current form. The authors report performance differences between model variants, but do not provide confidence intervals or statistical tests.

Including confidence intervals (or equivalent statistical measures) would clarify whether the observed differences are meaningful.

**Response:** We sincerely thank the reviewer for pointing out this critical omission. We fully agree that providing statistical measures of variance is essential for robustly evaluating the significance of the performance differences observed in our ablation study.

To address this, we have systematically updated Table 3 to include the standard deviation for all reported metrics. The results are now presented in a "Mean ± Std" format. These statistical measures were derived from [5-fold cross-validation] to ensure rigorous evaluation. The inclusion of these dispersion metrics clearly demonstrates the stability of our complete FusionPHI framework. By observing the standard deviations alongside the mean performance metrics, it is evident that the performance improvements achieved by the full model over the ablated variants are statistically meaningful and robust, rather than artifacts of random variance. We believe this addition substantially enhances the quantitative rigor and informativeness of our ablation analysis.

**Other modifications:**

1. In **the “Comparison with existing benchmark methods”** subsection, we found an ambiguity in the sentence: “To verify the generalization ability and robustness of the FusionPHI model, this study compares the AUC performance of FusionPHI with nine classical machine learning models on the PHIAF624 dataset(see Fig. ~\ref{fig6}).”. The dataset PHIAF was mistakenly written as **“PHIAF624”**, which could be misleading as referring to a different dataset. Therefore, we reviewed the entire text and standardized the description of the dataset, replacing **“PHIAF624”** with **“PHIAF”**.

***At last, we thank you sincerely for your arduous work and instructive advice. Overall the comments are fair, encouraging and constructive. We have learned much from them.***
