# Supplementary material for: FusionPHI: A phage-host interaction prediction network model based on attention-driven multi-modal feature fusion
Source: PLoS One. 2026 Aug 21;21(8):e0356238. doi: 10.1371/journal.pone.0356238 (PMC13497294; doi:10.1371/journal.pone.0356238)
Supplement: Supplementary file 2 [file pone.0356238.s003.docx]

**Response to Reviewers**

**PONE-D-25-60434R1**

FusionPHI: a phage-host interaction prediction network model based on attention-driven multi-modal feature fusion

*PLOS One*

Dear Editor and Reviewers,

We sincerely thank the editor and reviewers for their continued rigorous and constructive feedback during this second round of review. We have carefully considered all the additional comments and have thoroughly revised the manuscript accordingly. Detailed point-by-point responses are provided below. As before, all major revisions in the revised manuscript are highlighted in **RED** for ease of identification.

A. **Responses to Reviewer #3:**

**Comment 1:** Lacking the explainable AI part to understand the reason behind prediction which is relevant in biology.

**Response:** We sincerely thank the reviewer for this insightful and constructive comment. We fully agree that, for a biological problem such as phage–host interaction prediction, simply reporting predictive performance is insufficient and understanding the reasoning behind the predictions is essential for both model credibility and biological discovery. We are grateful that the reviewer raised this point, as it prompted us to substantially strengthen the interpretability of our work.

In the revised manuscript, we have added a dedicated section entitled “**Attention-based Feature Interaction Analysis**” to directly address this concern. In this section, we designed a multi-metric feature importance evaluation framework that combines four complementary measures: Pearson correlation, Random Forest Gini importance, permutation importance, and mutual information. All importance scores were normalized to enable fair comparison, and the results are visualized as a heatmap in **Fig 7**.

We also conducted a comprehensive analysis on the patterns of feature importance across different evaluation metrics and yielded several insights:

1) CG-enriched k-mer sequence features (e.g., the frequencies of CAGC, TCAG, CAGG, CCAG) show consistently high importance across all four metrics, indicating that they are robust and biologically meaningful signals for prediction.

2) Physicochemical features derived from amino acid compositions (e.g., the AAC frequencies for the amino acids D and G) display only moderate linear correlation with the labels, yet they achieve notably high permutation importance, suggesting that their predictive value is expressed through non-linear relationships that would have been missed by correlation-based selection alone.

3) Motif features (e.g., host_feature_96, host_feature_348, host_feature_264) exhibit balanced and moderate importance across all metrics, implying that they provide complementary information at the functional architecture level rather than relying on any single dominant signal.

Taken together, these findings demonstrate that our model integrates diverse feature modalities in a non-redundant manner, and, more importantly, that its predictions can be traced back to recognizable biological factors such as sequence motifs, physicochemical properties, and functional domains. We hope this addition satisfies the reviewer's request for explainable AI in this biological context.

We appreciate the reviewer's constructive feedback, which has significantly improved the manuscript's interpretability and biological relevance.

**Comment 2:** from the RefSeq .faa files whether all the gene annotations including hypothetical proteins are also used or not. It is not clearly mentioned in the manuscript

**Response:** We sincerely thank the reviewer for this careful and constructive comment. We apologize that this point was not stated clearly enough in the original manuscript.

In this study, host protein-coding sequences were obtained from the latest RefSeq assemblies. For each host species, we retrieved the assembly-provided CDS files and translated all coding sequences into amino-acid sequences. No filtering based on functional annotation was applied at either the nucleotide or the protein level. Consequently, the complete set of RefSeq CDS annotations was retained, including hypothetical proteins.

These sequences were then used to compute physicochemical features, after which the multi-ORF features were aggregated into a host-level representation. We revise the manuscript to describe this procedure explicitly in the last paragraph of the **“Datasets”** subsection so that the use of all annotated gene products, including hypothetical proteins, is unambiguously documented.

**Comment 3:** The dataset provided in the GitHub repository appears to be incomplete. The authors should consider depositing the complete dataset in a repository such as Zenodo. Additionally, one bacterial genome in the training dataset appears to be a partial genome (16S rRNA only). Please clarify whether partial genomes were included in the study and, if so, how they were handled.

**Response:** We sincerely thank the reviewer for this careful observation and the constructive suggestion. We apologize for the incomplete dataset initially shared on GitHub and this has now been fully rectified. The complete dataset, including all sequence files, metadata, and processing records, has been deposited in a permanent Zenodo repository **(DOI: 10.5281/21255334)**, and the GitHub repository has been updated to clearly direct users to this stable archive.

Regarding the inclusion of partial genomes, we appreciate the opportunity to clarify. During dataset construction, we prioritized retrieving complete genomes from NCBI. However, for several rare taxa of particular phylogenetic interest, no complete genome was available. To avoid introducing taxonomic gaps, we carefully retained high-quality partial genomes only when a complete alternative could not be found. The 16S rRNA-only sequence noted by the reviewer originated from a targeted locus project and was the sole representative for that species. Prompted by the reviewer’s comment, we carefully re-evaluated the quality and completeness of all partial sequences used in the study. For the 16S rRNA-only sequence, no higher-quality complete genome could be located, and removing it would have introduced a taxonomic gap. Therefore, we retained it but now explicitly acknowledge in the third paragraph of the **“Limitations”** subsection of the revised manuscript that partial genomes were included when complete genomes were unavailable, which may introduce potential biases.

We are deeply grateful to the reviewer for pointing out this issue, as addressing it has made our data selection criteria more transparent and rigorous.

**Comment 4:** The response to comment 4 from first reviewer explains the computational procedure but does not address the fundamental concern of the reviewer, whether arbitrarily translated motif sequences retain sufficient biological meaning to justify the use of ESM-2 protein embeddings.

**Response:** We sincerely thank you for this follow‑up comment, which urges us to address the deeper concern rather than merely the procedural details. We fully understand the worry that arbitrarily translated motif sequences, produced without respecting the native reading frame, may carry little biological meaning and thus call into question the use of ESM‑2 protein embeddings. We appreciate the opportunity to clarify our perspective on this point.

We completely agree that the resulting amino acid sequences are not genuine proteins, and we would never claim that they represent biologically expressed peptides. However, our aim has never been to model protein function or structure. Instead, we use the translation step as a consistent, information‑preserving transformation that maps conserved nucleotide patterns into an amino acid alphabet, solely for the purpose of generating dense feature representations via a pretrained model. ESM‑2 embeddings are known to capture general physicochemical properties (such as hydrophobicity, charge, and size) as well as contextual dependencies of amino acids, even for sequences that do not correspond to natural proteins. Because the motifs identified by MEME are themselves evolutionarily conserved nucleotide segments, forcing them through a fixed translation frame still retains the local sequence composition and order in a different symbolic space, which ESM‑2 can then encode into a rich feature vector. We do not treat these embeddings as functional annotations. Actually they serve as abstract representations of conserved genomic signals, much like k‑mer or one‑hot encodings, but with the advantage of leveraging the pretrained language model’s ability to capture higher‑order dependencies.

We have taken the reviewer’s fundamental concern very seriously. In the fourth paragraph of the **“Limitations”** subsection of the revised manuscript, we have substantially expanded the discussion to explicitly acknowledge the limitations: we now clearly state that the translated sequences are not biologically valid proteins, that the enforced reading frame may introduce artificial boundaries, and that the embeddings should be interpreted only as transformed features of conserved nucleotide motifs. We also note that, in the absence of direct experimental validation of whether these embeddings retain meaningful biological signal for the downstream task, this approach is best viewed as a pragmatic representation‑learning strategy that showed empirical benefit in our predictive task. We fully agree that further studies, possibly involving systematic ablation or comparison with random‑frame controls, would be valuable to rigorously quantify the biological relevance, and we have included this as a future direction.

We are deeply grateful to the reviewer for insisting on this fundamental issue, as it has led us to greatly improve the clarity and honesty of our presentation, and we hope our revised framing adequately addresses the concern.

**Comment 5:** The response to comment 8 from the first review only addresses pair level information leakage but does not sufficiently address the broader concerns regarding dataset construction, negative sampling, taxonomic distribution, data splitting strategy, and model generalizability.

**Response:** We sincerely thank the reviewer for this comment and for requiring a more comprehensive clarification of these interrelated aspects of our study. We fully acknowledge that our earlier response focused too narrowly on the information leakage issue, and we apologize for not addressing the other concerns with sufficient breadth and detail. We are grateful for the opportunity to provide a unified account here, which we have also reflected in the revised manuscript.

**Dataset construction.** The training dataset is built upon the publicly available PHP dataset, which provides 335 experimentally verified phage–host interaction pairs. We acknowledge that a processing oversight led to one positive pair (phage NC_042345 and host Xylella fastidiosa) being inadvertently duplicated, making the positive set 336 in the original manuscript. This error has been corrected, and the dataset now correctly comprises 335 positive pairs and 335 negative pairs (1:1 ratio). We have thoroughly re‑verified all sequence files, accession numbers, and pair counts, and updated the manuscript accordingly to ensure full consistency. More Detailed information can be seen in the **Datasets** subsection of the revised manuscript.

**Negative sampling.** In our study, negative pairs were generated by randomly pairing phages and hosts that are not known to interact, maintaining a 1:1 ratio with the positive pairs. We fully recognize that this balanced sampling strategy, while commonly adopted to ensure stable model training and unbiased evaluation, does not reflect the biological reality where true phage–host interactions represent only a very small fraction of all possible virus–host pairs. This discrepancy may lead the model to produce overconfident positive predictions when applied to severely imbalanced real-world screening scenarios. We have explicitly discussed this limitation in the revised **“Limitations”** subsection, where we state: *“we adopted a balanced 1:1 ratio of positive to negative pairs during training. While this is a common practice for stable model optimization, it does not reflect the biological reality... the model may tend to produce overconfident positive predictions when deployed in real-world screening scenarios with severe class imbalance, and its practical utility in such settings remains to be systematically evaluated.”* We believe this transparent acknowledgment appropriately conveys the current scope of our model.

**Taxonomic distribution.** We recognize that the dataset exhibits a long‑tailed taxonomic distribution: certain host genera (e.g., Escherichia, Pseudomonas, Staphylococcus) are heavily overrepresented, while many genera are represented by only one or a few instances. This imbalance may introduce taxonomic bias and lead to overestimation of performance on dominant taxa. We have addressed this concern in the revised manuscript by explicitly reporting the taxonomic composition of both the PHP and PHIAF datasets (summarized in **Fig 1** and **Fig 2**), discussing the potential impact on model generalization, and identifying the improvement of taxonomic balance as a key objective for future data expansion.

**Data splitting strategy.** For internal evaluation, we employed five‑fold cross‑validation using scikit‑learn’s KFold with shuffle=True and a fixed random seed (random_state=42). The splits were performed at the sample (pair) level, without enforcing grouping by phage or host identity. We fully acknowledge that this design may allow instances sharing the same phage or host to appear across training and validation folds, introducing a potential risk of within‑validation data leakage. However, we wish to emphasize that, in our study, five‑fold cross‑validation was used exclusively for hyperparameter tuning and model selection. All final performance results reported in the paper (including those in Table 4) were evaluated on a completely held‑out independent test set that was never involved in the cross‑validation process. Therefore, any leakage that occurred within the cross‑validation folds cannot have inflated the final reported metrics. At most, it could have led to suboptimal hyperparameter choices, but it does not compromise the validity of our performance evaluation or the comparative conclusions drawn from the test set. To further assess generalizability under a strict data separation paradigm, we employed the PHIAF dataset as an external benchmark. Furthermore, we performed a multi‑level overlap analysis between PHP and PHIAF, confirming that only 7 identical pairs exist (1.1% of the test set), and the vast majority of phage–host combinations are novel. We have acknowledged the absence of biological grouping in cross‑validation as a limitation in the revised **“Limitations”** subsection (**page 18**) , and we are grateful to the reviewer for prompting this clarification.

**Model generalizability.** We have approached generalizability from multiple angles. First, the external validation on PHIAF demonstrates that FusionPHI maintains competitive performance on a dataset with different taxonomic composition and host label conventions. Second, ablation studies across all feature branches (k‑mer, physicochemical, motif) reveal that each component contributes to overall robustness, and that the performance gains are not driven by a single brittle feature. Third, a case study on laboratory‑engineered strains shows that while the model can correctly predict most interactions, it can also fail when faced with heavily manipulated genomes, which we have honestly reported and discussed. Finally, the revised **“Limitations”** subsection now comprehensively addresses factors that constrain generalizability, including limited dataset scale, taxonomic skew, balanced class ratios, and sensitivity to genome quality, and it outlines clear directions for future improvement. We are confident that these combined discussions provide a transparent and realistic assessment of our model’s current generalization capability.

We hope that the above clarifications adequately address all the concerns raised by the reviewer. We are deeply grateful for the rigorous scrutiny, which has compelled us to significantly strengthen both our experimental reporting and the critical discussion of our method’s scope and limitations.

**Comment 6:** Italicizing organism name throughout the manuscript

**Response:** We sincerely thank the reviewer for this careful reminder. We have carefully gone through the entire manuscript and italicized all organism names (e.g., E. coli, Mycobacterium, etc.) throughout the text, tables, and figures.

**Comment 7:** Please check the AI content of the manuscript

**Response:** We sincerely thank the reviewer for this reminder. We confirm that AI‑based tools were used solely for language polishing and grammar checking during manuscript preparation. All scientific content, including the research ideas, model architecture, experimental design, data analysis, and conclusions, is entirely our original work. We have carefully reviewed the entire manuscript to ensure that the use of such tools did not introduce any factual inaccuracies or compromise scientific integrity.

B. **Responses to Reviewer #4:**

**General Comment:** FusionPHI: a phage-host interaction prediction network model based on attention-driven multi-modal feature fusion.

This manuscript presents FusionPHI, a multi-modal attention-driven framework for phage–host interaction (PHI) prediction. The proposed method integrates k-mer frequency features, protein physicochemical properties, and conserved motif embeddings via a dual-stage attention mechanism. The topic is timely and relevant, and the overall architecture is technically interesting. However, methodological, presentation, and consistency issues need to be addressed before the manuscript can be considered for publication.

**Response:** We sincerely thank you for your time and for this thoughtful and balanced summary of our manuscript. We are truly grateful for your positive assessment of the timeliness and relevance of our topic, and we are especially encouraged that you found the overall architecture of FusionPHI technically interesting. These generous comments mean a great deal to us.

We fully acknowledge that methodological, presentation, and consistency issues remain in the manuscript, and we take this criticism very seriously. In the point‑by‑point responses that follow, we have made every effort to address each specific concern as thoroughly and clearly as possible, and we have revised the manuscript accordingly. We deeply appreciate your guidance, which has substantially helped us improve the rigor and clarity of our work.

**Comment 1:**  Dataset inconsistencies and incomplete data usage.

After consulting the original PHP study, the test set appears to contain 671 virus-host interaction pairs, not 672 as stated by the authors. This discrepancy should be clarified. More importantly, it is unclear why the authors used only the PHP test set rather than the full merged dataset combining the PHP study and VirHostMatcher interactions. Furthermore, the 335 additional interaction pairs reported by the PHP authors on their GitHub repository do not appear to have been incorporated. The authors should explicitly justify these data choices and clarify the exact composition of their training dataset.

**Response:** We sincerely thank the reviewer for this meticulous examination and for identifying the numerical discrepancy. Upon re‑examining our dataset construction pipeline, we indeed found a processing oversight that led to the incorrect count of 672 pairs. The original positive interaction pairs from the PHP study total 335. During training set construction, one positive pair—between phage NC_042345 and host Xylella fastidiosa—was inadvertently duplicated. This caused the positive set to inflate to 336, and because we maintained a 1:1 ratio of negative to positive samples, the negative set was correspondingly enlarged, resulting in a total of 672 pairs rather than the intended 670. We sincerely apologize for this error and any confusion it may have caused. We have now corrected the number of PHI pairs in the training set to 670 (335 positive + 335 negative) in the first paragraph of the **“Datasets”** subsection, and thoroughly re‑checked all data‑related numbers and splits throughout the revised manuscript to ensure full consistency.

Regarding the choice of the data source, we confirm that the 335 positive interaction pairs we used are exactly the publicly available dataset provided by the PHP authors on their GitHub repository. Therefore, these additional pairs have in fact been fully incorporated into our training set and we regret that our original description may have given the impression that they were omitted. We have now clarified the exact provenance and composition in the first paragraph of the **“Datasets”** subsection of the revised manuscript.

As for not merging the PHP test set with VirHostMatcher (VHM) interactions to create a larger combined dataset, this decision was deliberately made to avoid the risk of data leakage. Because the two datasets were originally compiled under different study designs, screening criteria, and laboratory protocols, directly merging them could unintentionally introduce overlapping or highly similar sequence pairs that span the training and testing partitions. Such leakage would inflate the perceived model performance and undermine the rigor of the evaluation. Therefore, we elected to use the PHP dataset alone, which provides a clean, self‑contained benchmark with well‑documented provenance.

We are deeply grateful to the reviewer for prompting these clarifications, which have greatly improved the accuracy and transparency of our data reporting.

**Comment 2:** Fairness of baseline comparisons (Table 2).

The comparative evaluation presents results for VHM, PHP, PHIAF, vHULK, PB-LKS, and PHPGAT sourced from Nie's review rather than obtained through independent re-evaluation. It is unclear whether all methods were assessed under identical experimental conditions and on the same dataset. If the reported performance values originate from different datasets or evaluation protocols, the observed performance gains may not be attributed to the proposed model alone. The authors must either rerun all baseline methods on the same dataset under the same conditions, or explicitly acknowledge this as a critical limitation of the comparison. Furthermore, Table 2 is missing AUC and MCC values for most baseline methods.

**Response:** We sincerely thank the reviewer for this rigorous comment and for the clear guidance on baseline comparison fairness. We fully agree that cross-paper comparisons risk introducing biases due to differing evaluation protocols, and we also acknowledge that our original Table 2 omitted critical metrics for most baselines.

To address these concerns comprehensively, we have made the following revisions to our manuscript:

1) **Explicit acknowledgment of the limitation**. We fully concur with the reviewer that relying on third-party results for non-re-runnable baselines constitutes a critical limitation. Following the reviewer’s suggestion, we have added a dedicated statement in the penultimate paragraph of the “**Limitations**” subsection in the revised manuscript, clarifying that the baseline results drawn from Nie et al.’s survey serve as system-level references, and that differences in evaluation protocols may affect the direct attribution of observed performance gains.
2) **Independent re-evaluation of vHULK under identical conditions**. To provide a fair and controlled comparison, we re-implemented vHULK, the only baseline tool that shares a deep learning framework and Python implementation with FusionPHI, allowing for a controlled comparison under strictly identical computational settings. This allowed us to compute the complete set of six metrics, including the previously missing AUC and MCC, under identical experimental conditions. As presented in the revised **Table 4** (formerly Table 2), vHULK-run achieves an AUC of 0.52 and an MCC of 0.03 on our benchmark dataset, while FusionPHI reaches an AUC of 0.86 and an MCC of 0.66, demonstrating a substantial and fair improvement. The originally reported vHULK figures from Nie et al. are retained alongside our re-run results for direct side-by-side reference.

3) **Clarifying the absence of AUC and MCC values for other baselines.** We agree with the reviewer that these metrics are important for a comprehensive evaluation. However, the AUC and MCC values were not reported in the source review for the remaining baseline methods (VHM, PHP, PHIAF, PB-LKS, and PHPGAT). We carefully assessed the feasibility of re-running all baselines, whereas vHULK is the only baseline tool that shares a deep learning framework and Python implementation with FusionPHI, allowing for a controlled comparison under identical computational setting. Re-evaluating the remaining baselines would require substantial re-engineering across different environments, potentially introducing additional confounding factors and undermining the fairness of the comparison. Therefore, in the revised **Table 4**, we have left the corresponding cells blank for these methods and added a footnote clearly explaining the reason, ensuring full transparency.

4) **Updated discussion of baseline comparisons.** We have revised the **Comparison with existing benchmark methods** subsection to place greater emphasis on the strictly controlled comparison against vHULK-run and to appropriately contextualize the remaining literature-based comparisons as supplementary references. The updated text highlights that FusionPHI consistently surpasses all baselines in the metrics reported by Nie et al., and substantially outperforms vHULK under the fair, re-run setup.

We are deeply grateful for this comment, which has driven us to present our comparisons with greater accuracy, transparency, and intellectual honesty. All related changes have been carefully incorporated into the revised manuscript, including **Table 4**, the corresponding in-text descriptions, and the “**Limitations**” subsection.

**Comment 3:**  Cross-validation construction and internal data leakage.

The authors do not describe how the five-fold cross-validation splits were constructed. Specifically, it is not stated whether phage or host identities were held out across folds to prevent within validation leakage.

**Response:** We sincerely thank the reviewer for this valuable comment. We agree that the description of our five-fold cross-validation procedure in the original manuscript was insufficiently detailed, and we appreciate the opportunity to clarify.

In our experiments, the dataset was partitioned into five folds using scikit-learn’s KFold with shuffle=True and random_state=42. Splitting was performed at the sample level, and the fixed random seed ensures full reproducibility of the fold assignments. We have added the description of the implementation detail of the five-fold cross-validation splits in the **“Experiment setting”** subsection of the revised manuscript.

More importantly, we fully acknowledge the reviewer’s concern about whether phage or host identities were held out across folds to prevent within-validation leakage. In our current implementation, the folds were constructed without enforcing any grouping by biological entity, which may indeed allow instances with the same phage or host to appear in both training and validation folds. We recognize that this introduces a potential risk of data leakage. However, we wish to respectfully clarify that, in our study, five-fold cross-validation was employed solely for hyperparameter tuning and model selection. All final performance results reported in the paper (including those in Table 4) were evaluated on a completely held-out independent test set that was never involved in the cross-validation procedure. Therefore, any leakage that occurred within the cross-validation folds cannot have inflated the final reported metrics, as these metrics were derived strictly from the independent test set. The cross-validation leakage may at most have led to suboptimal hyperparameter choices, but it does not compromise the validity of our performance evaluation or the comparative conclusions drawn from the test set.

Nevertheless, we agree that the absence of biological grouping in the cross-validation setup is a limitation of our current experimental protocol, and we have added a note in the revised **“Limitations”** subsection (**page 18**) to acknowledge this point. We are sincerely grateful for this insightful comment, which has prompted us to strengthen both the transparency and the rigor of our experimental reporting.

**Comment 4:**  Data leakage argument between PHP and PHIAF datasets is not fully convincing.

The authors argue that no pair-level data leakage exists between the PHP training dataset and the PHIAF test dataset, based on the observation that host information is recorded differently across the two datasets, species names in PHP versus genome accession identifiers in PHIAF. However, this argument is not fully convincing, as different representations do not necessarily imply different biological entities. For example, a species name such as Escherichia coli in PHP and a genome accession in PHIAF could refer to the same organism. To properly rule out leakage, the authors should map the PHIAF host accession identifiers to their corresponding species names and explicitly verify that no overlapping phage-host pairs exist at the biological level, not merely at the label level. Additionally, the authors state that no "identical" phage-host pairs exist across datasets, but it is unclear whether "identical" refers strictly to exact label matches or also accounts for genome sequence similarity, which could also introduce leakage.

**Response:** We sincerely thank the reviewer for this rigorous and thoughtful comment. We fully acknowledge that our original reasoning, relying on different host label formats, was insufficient, and we appreciate the opportunity to provide a more thorough, biologically grounded analysis.

To address this concern properly, we first mapped each host accession identifier in PHIAF to its corresponding species name using our curated taxonomy table, allowing a direct comparison at the biological level. We then examined the overlap between PHP and PHIAF at the species, genus, and family levels. The results are presented in **Table 1~3** of the revised manuscript. In summary, PHP and PHIAF share 26 host species, which correspond to 49 overlapping genera and 18 overlapping families. At the strict pair level, we identified 7 interaction pairs that appear identically in both datasets, i.e., the same phage genome paired with the same host species, accounting for only 1.1% of the 624 PHIAF test pairs.

We recognize that the presence of these 7 identical pairs does represent a form of data leakage at the pair level, and we should not dismiss it. We apologize for the previously imprecise wording and have corrected it in the revised manuscript. However, we would like to clarify why this minimal leakage is unlikely to meaningfully distort our evaluation of generalization, given the fundamental nature of phage–host interaction prediction.

Phage-host interaction prediction is inherently a problem of learning compatibility signals between two genomes, rather than of recognizing individual entities. Our model relies on features such as k‑mer frequency similarity, protein physicochemical profiles, and conserved motif patterns, all of which capture various dimensions of molecular “fit” between a phage and a host. In this context, the discriminative information is not locked within a single genome, but emerges from the relational pattern between the two partners. When a training phage and a test phage share the same genome but are paired with different hosts, the learned representation of that phage remains useful, yet the pairwise compatibility signature shifts due to the different host context. Consequently, even if a small number of identical pairs exist, the model cannot simply memorize them to achieve high performance on the independent test set and it must still generalize the compatibility rules that apply across distinct phage-host pairings. The fact that 98.9% of the PHIAF test pairs are completely novel pairings further supports that our reported performance reflects true generalization rather than data memorization.

Thus, while we acknowledge the existence of 7 identical pairs as a limitation, we believe their impact on the overall assessment is marginal given the relational nature of the prediction task. We have explicitly listed these 7 pairs in a new **Table 2** within the **“Datasets”** subsection and added a transparent discussion of the overlap. We sincerely thank the reviewer for pressing us on this point, which has led to a more honest and rigorous reporting of our data composition.

**Comment 5:** Case study results contradict the paper's main claims.

The case study in its current form undermines rather than supports the paper's central contribution. As shown in Table 4, the ablated model Attn-KP, which excludes motif embeddings, correctly predicts all five phage–host interaction pairs, while FusionPHI produces incorrect predictions for DH5α and Rosetta⋆(DE3)plys. The authors attribute FusionPHI's errors to data quality issues arising from manual sequence splicing introducing false motif signals. While this explanation is plausible, it simultaneously highlights a critical fragility of the motif feature, the paper's primary novelty. The authors should provide a more thorough analysis of when motif features contribute positively versus when they introduce noise, and this vulnerability should be explicitly acknowledged in the Limitations section.

**Response:** We sincerely thank the reviewer for this incisive observation and for pressing us to more honestly examine the conditions under which motif features are beneficial. We fully agree that the case study inadvertently exposes a fragility of the motif-based representation, which is indeed the core novelty of our work, and that this deserves transparent acknowledgment and deeper analysis rather than a passing mention.

Prompted by the reviewer’s comment, we have substantially expanded the **“Limitations”** section in the revised manuscript to include a dedicated discussion of this vulnerability (**page 17**). Specifically, we now clarify that the motif feature contributes positively when the input genomes are of high quality, with complete or near-complete assemblies, and when the conserved regions identified by MEME reflect genuine evolutionary signals that are stably present across related taxa. Under these conditions, the motif embeddings provide complementary phylogenetic information that helps the model capture subtle sequence signatures beyond k‑mer composition and physicochemical properties, as evidenced by the overall performance gains in our ablation experiments. Conversely, the motif feature tends to introduce noise when the genome sequences are fragmented, contain assembly errors, or have undergone manual splicing that disrupts the natural context of conserved elements. In such cases, MEME may identify spurious motifs on artificially concatenated boundaries or within misassembled segments, and these false motifs can mislead the attention mechanism into attending to irrelevant signals, ultimately degrading prediction accuracy. This exactly explains why FusionPHI failed on the DH5α and Rosetta⋆(DE3)plys strains in the case study, whereas Attn‑KP, which does not use motif information, remained unaffected. We explicitly state that the reliability of motif‑based features is therefore contingent on input sequence integrity, and that this dependency represents a current limitation of our approach when applied to engineered or partially assembled genomes.

We believe this expanded discussion directly addresses the reviewer’s concern by clearly delineating the boundary conditions for the motif feature’s utility. We also note that developing automated quality-control filters for motif reliability and exploring robust feature fusion strategies that can dynamically down-weight potentially noisy motifs are important directions for future improvement. We are deeply grateful to the reviewer for highlighting this issue, as it has prompted us to present a more balanced and rigorous assessment of our proposed method.

**Comment 6:** Positive-to-negative sample ratio.

The authors use a 1:1 ratio of positive to negative PHI samples. While this is a common choice for balanced classification, it does not reflect the biological reality, where true phage-host interactions represent a small fraction of all possible pairs. The authors should discuss whether and how this imbalance affects the generalisability of the model to real-world screening scenarios.

**Response:** We sincerely thank the reviewer for this insightful and practically important comment. We fully agree that the 1:1 positive-to-negative ratio adopted in our study, although commonly employed to ensure stable training and unbiased evaluation of classification models, does not reflect the true biological landscape, where genuine phage–host interactions constitute only a very small fraction of all possible virus–host pairs.

Our decision to use a balanced training set was primarily driven by the goal of fairly validating the multimodal fusion architecture itself without the confounding effect of extreme class imbalance, which could skew the model towards the majority class and obscure the actual discriminative capability of the learned features. We acknowledge, however, that this design choice limits the direct assessment of how FusionPHI would perform in a real-world screening scenario, where the prior probability of a true interaction is extremely low. Under such highly imbalanced conditions, a model trained on balanced data may tend to produce overly confident positive predictions, leading to an elevated false positive rate unless appropriate decision threshold tuning or post-hoc calibration is applied.

Prompted by the reviewer’s concern, we have accordingly updated the **“Limitations”** subsection (**page 17)** to explicitly discuss this issue in the revised manuscript. We note that the current performance figures should be interpreted as the model’s discriminative capacity under a controlled balanced setting, and that deploying FusionPHI in a large-scale discovery context would require additional strategies such as adjusting the decision threshold based on the estimated prevalence, incorporating cost-sensitive learning, or using the model as a scoring function combined with a high-specificity cutoff. We also point out that evaluating the model on progressively more imbalanced test sets and exploring dedicated techniques for learning from imbalanced genomic interaction data represent important directions for future work. We are deeply grateful to the reviewer for raising this point, which has helped us present a more complete and honest picture of our model’s current scope and its path toward practical applicability.

**Minor Comment:**

Line 16: The phrase "predict whether an interaction exists is likely to occur" is unclear.

Line 17: "in vivo" should be italicised, as is standard for Latin terms in scientific writing. The same applies to all instances of de novo (line 186) and any other Latin terms throughout the manuscript, including those appearing in figures.

Line 32: The inclusion of PhiSpy appears out of place. PhiSpy is designed to identify prophage regions within bacterial genomes, which is a distinct task from PHI prediction. The authors should either clarify how PhiSpy relates to the PHI prediction pipeline or relocate it to a more appropriate context.

Line 38: Reference (16) is cited without author attribution ("research by (16) has demonstrated..."). At minimum, the first author's name should be included, e.g. "Smith et al. (16) demonstrated...".

Line 130: The term "de-emphasis" is unclear.

Lines 134 and 165: Line 134 states the final dataset contains "333 phage genomes", while line 165 states "the PHP dataset contains 336 unique phage genomes". This inconsistency should be resolved and explained.

Lines 156–161: The repeated genus names (e.g. "Mycolicibacterium (Mycobacterium)", "Shigella (Shigella genus)") are redundant.

Line 164: "information leakage" should be replaced with "data leakage".

Line 245: A period seems to be missing.

Line 263: The extended acronym for PHI is reintroduced unnecessarily, having already been defined earlier.

Figure 3 caption: The sentence "High-dimensional embedding representations were extracted." appears to be a redundant leftover fragment.

Figure 5 caption: typo "corss-attention".

Figures 7A–7E: The image quality is insufficient. The authors should provide higher resolution images and consider adding annotations to guide the reader.

Line 401: During the comparison against the PHIAF dataset, it should be clarified whether all models were evaluated on the same test set, with only the classification model varying. This needs to be made explicit to ensure the comparison is interpretable.

Conclusions, line 569: The stated AUC of 91% is inconsistent with Table 3, which reports an AUC of 0.87 ± 0.012 for the full model. Please verify and correct.

We sincerely thank the reviewer for these careful and constructive minor comments. We have carefully considered all of them and have made corresponding revisions throughout the manuscript to improve clarity, consistency, and presentation quality. We are grateful for this meticulous attention to detail, which has substantially improved the accuracy and readability of our work.

**Other modifications:**

1. In the **Case Study** subsection, we found an ambiguity in the sentence: “The experiments were carried out using a technical route combining the gradient dilution method with empty spot counting. Four concentration gradients (10^8, 10^9, 10^10, and 10^11 -fold dilutions) were prepared by serial dilution of phage suspensions with an initial titre 7×10^13 pfu/mL. The **“×”** sign is not displayed correctly in the compiled PDF document. In response to this, we revised the LaTeX document and replaced the **“×”** sign with **“\times”**. Similar situations in the revised manuscript have also been reviewed and corrected.

Once again, we would like to express our sincere gratitude for your continued rigorous and thoughtful evaluation. The comments in this second round of review remain fair, constructive, and immensely instructive. We have learned a great deal from them and deeply appreciate the time and expertise you have so generously devoted to helping us strengthen this work.

Yours sincerely,

Qingting Wei

School of Software, Nanchang University

235 East Nanjing Road, Nanchang 330047, Jiangxi, China

Email: qtwei@ncu.edu.cn
