# Supplementary material for: FusionPHI: A phage-host interaction prediction network model based on attention-driven multi-modal feature fusion
Source: PLoS One. 2026 Aug 21;21(8):e0356238. doi: 10.1371/journal.pone.0356238 (PMC13497294; doi:10.1371/journal.pone.0356238)
Supplement: Peer review history

# Peer review: Decision Letter 0

**DOI:** 10.1371/journal.pone.0356238.r001

**Authors:** Ivan Petrushin

7 Apr 2026

Dear Dr. Wei,

Thank you for submitting your manuscript to PLOS ONE. After careful consideration, we feel that it has merit but does not fully meet PLOS ONE’s publication criteria as it currently stands. Therefore, we invite you to submit a revised version of the manuscript that addresses the points raised during the review process.

Please submit your revised manuscript by May 22 2026 11:59PM. If you will need more time than this to complete your revisions, please reply to this message or contact the journal office at plosone@plos.org. When you're ready to submit your revision, log on to https://www.editorialmanager.com/pone/ and select the 'Submissions Needing Revision' folder to locate your manuscript file.

- A letter that responds to each point raised by the academic editor and reviewer(s). You should upload this letter as a separate file labeled 'Response to Reviewers'.
- A marked-up copy of your manuscript that highlights changes made to the original version. You should upload this as a separate file labeled 'Revised Manuscript with Track Changes'.
- An unmarked version of your revised paper without tracked changes. You should upload this as a separate file labeled 'Manuscript'.

If you would like to make changes to your financial disclosure, please include your updated statement in your cover letter. Guidelines for resubmitting your figure files are available below the reviewer comments at the end of this letter.

If applicable, we recommend that you deposit your laboratory protocols in protocols.io to enhance the reproducibility of your results. Protocols.io assigns your protocol its own identifier (DOI) so that it can be cited independently in the future. For instructions see: https://journals.plos.org/plosone/s/submission-guidelines#loc-laboratory-protocols. Additionally, PLOS ONE offers an option for publishing peer-reviewed Lab Protocol articles, which describe protocols hosted on protocols.io. Read more information on sharing protocols at https://plos.org/protocols?utm_medium=editorial-email&utm_source=authorletters&utm_campaign=protocols.

As the corresponding author, your ORCID iD is verified in the submission system and will appear in the published article. PLOS supports the use of ORCID, and we encourage all coauthors to register for an ORCID iD and use it as well. Please encourage your coauthors to verify their ORCID iD within the submission system before final acceptance, as unverified ORCID iDs will not appear in the published article. *Only*  the individual author can complete the verification step; PLOS staff *cannot*  verify ORCID iDs on behalf of authors.

We look forward to receiving your revised manuscript.

Kind regards,

Ivan S Petrushin, Ph.D

Academic Editor

PLOS One

**Journal Requirements:**

When submitting your revision, we need you to address these additional requirements.

1. Please ensure that your manuscript meets PLOS ONE's style requirements, including those for file naming. The PLOS ONE style templates can be found at

https://journals.plos.org/plosone/s/file?id=wjVg/PLOSOne_formatting_sample_main_body.pdf and

https://journals.plos.org/plosone/s/file?id=ba62/PLOSOne_formatting_sample_title_authors_affiliations.pdf

2. We note that the grant information you provided in the ‘Funding Information’ and ‘Financial Disclosure’ sections do not match.

When you resubmit, please ensure that you provide the correct grant numbers for the awards you received for your study in the ‘Funding Information’ section.

3. Thank you for stating the following financial disclosure:

“This work was supported in part by the National Natural Science Foundation of China (Grant Nos. 62362050, 62562047, 32260248), the Jiangxi Provincial Natural Science Foundation(Grant No.20252BAC240669), the Jiangxi Provincial Key Laboratory of Data Security Technology (Grant No.20242BCC32026), the Key Research and Development Program of Jiangxi Province (Grant No.20243BBG71035), the Finance Science and Technology Special ”Contract System” Project of Jiangxi Province (Grant Nos. ZBG20230418001, ZBG20230418014), the Market Supervision Administration Science and Technology Project of Jiangxi Province (Grant No.GSJK202305), the Nanchang University College Students’ Innovation Training Program Project (Grant No.2024CX147).”

Please state what role the funders took in the study.  If the funders had no role, please state: "The funders had no role in study design, data collection and analysis, decision to publish, or preparation of the manuscript."

If this statement is not correct you must amend it as needed.

Please include this amended Role of Funder statement in your cover letter; we will change the online submission form on your behalf.

4. Please note that funding information should not appear in any section or other areas of your manuscript. We will only publish funding information present in the Funding Statement section of the online submission form. Please remove any funding-related text from the manuscript.

5. In the online submission form you indicate that your data is not available for proprietary reasons and have provided a contact point for accessing this data. Please note that your current contact point is a co-author on this manuscript. According to our Data Policy, the contact point must not be an author on the manuscript and must be an institutional contact, ideally not an individual. Please revise your data statement to a non-author institutional point of contact, such as a data access or ethics committee, and send this to us via return email. Please also include contact information for the third party organization, and please include the full citation of where the data can be found.

6. Please note that your Data Availability Statement is currently missing the repository. If your manuscript is accepted for publication, you will be asked to provide these details on a very short timeline. We therefore suggest that you provide this information now, though we will not hold up the peer review process if you are unable.

7. Please include captions for your Supporting Information files at the end of your manuscript, and update any in-text citations to match accordingly. Please see our Supporting Information guidelines for more information: http://journals.plos.org/plosone/s/supporting-information.

8. If the reviewer comments include a recommendation to cite specific previously published works, please review and evaluate these publications to determine whether they are relevant and should be cited. There is no requirement to cite these works unless the editor has indicated otherwise.

[Note: HTML markup is below. Please do not edit.]

Reviewers' comments:

Reviewer's Responses to Questions

**Comments to the Author**

1. Is the manuscript technically sound, and do the data support the conclusions?

Reviewer #1: Partly

Reviewer #2: Yes

**********

2. Has the statistical analysis been performed appropriately and rigorously?

Reviewer #1: I Don't Know

Reviewer #2: No

**********

3. Have the authors made all data underlying the findings in their manuscript fully available?

The PLOS Data policy

Reviewer #1: No

Reviewer #2: Yes

**********

4. Is the manuscript presented in an intelligible fashion and written in standard English?

Reviewer #1: Yes

Reviewer #2: Yes

**********

Reviewer #1: This manuscript presents FusionPHI, a deep learning model for phage–host interaction (PHI) prediction based on multi-modal feature fusion: genomic k-mer frequencies, protein physicochemical properties, and motif-level embeddings derived from ESM-2, integrated via a dual-stage attention-driven feature fusion module. The authors benchmark FusionPHI against several existing PHI tools and include both ablation studies and a small experimental case study with M13K07 and several E. coli strains.

The topic is timely and clearly within the scope of PLOS ONE. The study appears to report original research and the approach is promising. However, there are important issues regarding methodological clarity, data availability, and explanation of the model to a biological audience that, in my view, require substantial revision.

First, the strengths of the manuscript:

Phage–host prediction remains a key bottleneck for interpreting metagenomic data and for phage therapy applications. A model that integrates multiple sequence-derived modalities is highly relevant to the community. The authors provide a good introduction that is consistent with the current state of knowledge, both regarding the challenges of phage therapy and the relevant bioinformatics problems.

The idea of combining: genomic k-mer features, aggregated protein physicochemical descriptors, and motif-level embeddings derived from a large protein language model (ESM-2) is conceptually appealing and could potentially capture complementary aspects of phage–host compatibility. New algorithms addressing the phage–host matching problem are very important.

The dual-stage self-/cross-attention feature fusion module is an interesting design choice for weighting and integrating heterogeneous feature types. The inclusion of ablation experiments to dissect the contribution of different feature sets and attention blocks is a clear strength.

The experimental infection assays with M13K07 and several E. coli strains, although limited in scope, are a welcome attempt to connect computational predictions with real biological behavior.

The authors provide a GitHub repository with the implementation of FusionPHI, which is an important step towards reproducibility.

Major comments:

The manuscript repeatedly states that motif embedding vectors constitute an “innovative feature”:

“In particular, we introduce an innovative feature, that is, the embedding vectors of the gene motif sequence.”

However, the novelty is not clearly defined or justified.

Protein language models (including ESM-2) have already been used to compute embeddings for protein sequences and sometimes for protein fragments/domains.

It is not clear in what precise sense motif-level embeddings are conceptually new compared with: embedding entire ORFs or full protein sequences, or using other fragment-based embeddings.

Please more clearly position FusionPHI relative to other recent multi-modal or attention-based PHI methods: which components are conceptually new (e.g. the dual-stage cross-attention design, use of motif-level ESM-2 embeddings), and which are extensions of earlier work?

The text mentions that “27 original features” per sample are expanded to 81-dimensional vectors, but it is not initially clear what these 27 features are. From the Methods, one can infer that they correspond to:

7 physicochemical indices (length, pI, molecular weight, aromaticity, instability index, flexibility, GRAVY), plus

20 amino acid composition frequencies.

I suggest explicitly stating this in the text before introducing the expansion to 81 dimensions, so that the origin of the “27 features” is transparent.

The model uses k = 4 for genomic k-mer frequencies, but the choice of this value is not justified.

Were other k values (e.g. 3, 5, or combinations) tested?

Is k = 4 chosen based on previous work on the same dataset, or on a systematic performance comparison?

Given that k-mer choice can substantially affect performance and feature dimensionality, please either:

justify k = 4 with references and/or include a brief sensitivity analysis showing that k = 4 is a reasonable choice.

The manuscript states:

“Subsequently, in order to more accurately capture the protein functional properties of these conserved regions, we performed transcription and translation operations on each motif mk to generate its corresponding RNA sequence rk and protein sequence pk, respectively.”

If motifs are translated without strict alignment to the original ORF frame, ESM-2 may be embedding artificial peptide sequences, which could compromise the biological meaning of the features.

Please clarify the exact procedure: how motifs are mapped to ORFs, how the reading frame is determined, and how non-frame or non-multiples-of-three motifs are handled (filtered, trimmed, or still translated).

The dual-stage attention-driven fusion module (self-attention followed by two cross-attention blocks) is central to FusionPHI. The current description is mathematically correct but quite difficult to follow for readers without a deep learning background.

I recommend adding a brief, intuitive explanation, for example:

what self-attention is expected to capture within each modality (e.g. which k-mers or motif features are most informative), what cross-attention is expected to capture between modalities (e.g. aligning specific motif patterns with particular genomic patterns or protein properties), how the two stages complement each other.

A small schematic or a simple toy example would substantially improve accessibility for biologists and bioinformaticians not specializing in ML.

The manuscript states that ORF sequences or gene sequences are used, but it is not sufficiently clear how these were obtained:

Were ORFs taken from existing annotations in NCBI/RefSeq or from the PHP dataset?

Were they predicted de novo (e.g. using NCBI ORFfinder, Prodigal, or another tool)? If so, with which parameters?

In the case study discussion, the authors note that for the BL21⋆(DE3)pLysS host, they used an “UNVERIFIED, partial pfkB gene sequence” from NCBI, and they attribute a misprediction to this low-quality, truncated sequence. This raises two concerns:

It contradicts the earlier impression that complete genomes or curated ORF sets are consistently used to represent hosts.

It is unclear why such a partial, unverified gene sequence was used at all, rather than a complete genome assembly or curated annotation.

Please: clarify, for all hosts, whether full genomes or partial genes were used, and using what pipeline; justify the inclusion of partial, unverified sequences or, preferably, exclude them and treat such issues as dataset limitations rather than simply attributing errors to the model.

The dataset description is quite brief and leaves several important questions open:

Negative pair construction:

How were non-interacting phage–host pairs generated from the PHP resource? Random pairing? Any biological or taxonomic constraints?

Taxonomic distribution:

What is the distribution of host species and phage species/families? For example, how many hosts belong to Enterobacteriaceae or other dominant families?

Train/validation/test splitting:

Are folds split at the pair, phage, or host level? Do the same phage or host genomes appear in both training and test sets? Without careful splitting (e.g. host- or phage-level segregation), there is a risk of information leakage and overestimation of performance.

Whether any precautions were taken to prevent information leakage (e.g. the same genome contributing to both training and test)?

These details are critical for interpreting the reported AUC and for judging generalizability.

It is not fully clear on which dataset(s) and at which taxonomic level these numbers are obtained, and whether all tools are evaluated under the same conditions (same input data, same host taxonomy level, same negative sampling strategy). Is this tool suggested to solve PHI problem on genus/species/strain level? The 1 case study is not enough in the opinion of this reviewer.

Please provide:

a more detailed description of dataset construction, including how many phages and hosts per family, a clear explanation of how negative pairs are defined, and an explicit statement of the splitting strategy and any stratification by taxonomy.

The Data Availability statement and the GitHub repository suggest that code and data are available, but on inspecting the repository one finds only example training samples, not the full datasets used for the analyses.

The M13K07 / E. coli case study is a valuable addition, but several points remain unclear:

Strain selection:

Why were BL21⋆(DE3)pLysS, BL21(DE3), DH5α, Rosetta⋆(DE3)pLysS, and TG1 chosen specifically? Are these strains known or suspected to be non-permissive for M13/M13K07, or is this being tested for the first time? If relevant literature exists, please cite it.

Relationship to the training set:

How strongly are Enterobacteriaceae, and especially E. coli, represented in the training dataset? Is there a risk that the model is biased or overfitted toward certain families or strains?

How other tools used in the benchmark predict the outcome of the experiment?

Stratification between train and case study:

Were closely related E. coli strains (or the same strains) present in the training data? If so, please discuss how this affects the interpretation of the case study as an “independent” validation.

Sequence quality issue (BL21 partial pfkB):

As mentioned above, the use of a partial, unverified gene sequence to represent the host is problematic. At minimum, this should be clearly acknowledged as a limitation of the dataset and not just a post hoc explanation for a misprediction; ideally, low-quality entries should be filtered out and the analysis updated.

Clarifying these aspects would make the case study more convincing and biologically interpretable.

Many of the limitations of the study are implicit (dataset size, quality of annotations, single experimental system, possible taxonomic bias), but there is no dedicated paragraph or section that clearly summarizes them.

I strongly recommend adding a short “Limitations” subsection in the Discussion, explicitly covering:

limited dataset size, potential taxonomic biases and possibility of overfitting to overrepresented families, dependence on high-quality and complete host and phage sequences, the narrow scope of experimental validation (one phage–host system). This will strengthen the balance and transparency of the manuscript.

Figure(s) showing plaque assays are of relatively low resolution in the PDF, and plaques are difficult to see clearly.

Phrases such as “overcomes fundamental limitations in existing single-modality approaches” and very strong “state-of-the-art” claims may be slightly overstated given the dataset size and limited experimental validation. I suggest softening such statements and clearly separating benchmark performance from broader biological generalization.

Reviewer #2: The manuscript presents an interesting approach to motif-based sequence analysis and proposes a novel model. While the topic is relevant and potentially suitable for PLOS ONE, the current version of the manuscript requires substantial revision to meet the journal’s standards of methodological clarity, reproducibility, and rigor.

Major comments:

1. The authors claim that their model outperforms existing approaches; however, this conclusion is not sufficiently supported by the presented results. In Fig. 3, the SVM baseline appears to perform comparably, and potentially more consistently, than the proposed method.

Additionally, the use of a boxplot with only five data points is potentially misleading. A swarmplot or an alternative visualization would provide a clearer representation of the data distribution. Regardless of visualization, the reported differences between the proposed model and SVM do not appear to be statistically significant, which weakens the central claim of superiority.

2. The manuscript does not clearly describe the feature sets used to train the baseline models. This makes it difficult to assess whether the comparison is fair.

Furthermore, the authors state that their model relies less on hand-crafted features. However, this claim is questionable, as the model input consists of preprocessed representations rather than raw DNA or protein sequences. The preprocessing pipeline itself effectively constitutes feature engineering and should be described and discussed accordingly.

3. The preprocessing approach raises several important questions. Specifically, it is unclear why MEME was executed with DNA-specific parameters to identify motifs that appear to be protein-related.

This configuration is typically more appropriate for identifying transcription factor binding motifs. Moreover, the subsequent conversion of identified motifs into RNA and protein sequences is not sufficiently justified and does not appear to add clear biological meaning.

The authors should provide a detailed and biologically grounded rationale for this workflow.

4. There are concerns regarding the evaluation procedure. In Fig. 3, some methods exhibit ROC-AUC values significantly below 0.5. This suggests that the models may be learning an inverse relationship (i.e., systematically predicting the opposite class), which requires explanation.

The manuscript would benefit from:

1)A clearer description of dataset construction

2)An explicit discussion of train/test splits

3) An analysis of sequence similarity between training and test sets

Without this information, it is difficult to rule out potential data leakage or an unfair advantage for models that can memorize training data.

5. The ablation study is not sufficiently informative in its current form. The authors report performance differences between model variants, but do not provide confidence intervals or statistical tests.

Including confidence intervals (or equivalent statistical measures) would clarify whether the observed differences are meaningful.

**********

what does this mean?). If published, this will include your full peer review and any attached files.

If you choose “no”, your identity will remain anonymous but your review may still be made public.

**Do you want your identity to be public for this peer review?**  For information about this choice, including consent withdrawal, please see our Privacy Policy

Reviewer #1: **Yes:** MARCIN K LUBOCKI

Reviewer #2: **Yes:** Dmitry Penzar

**********

[NOTE: If reviewer comments were submitted as an attachment file, they will be attached to this email and accessible via the submission site. Please log into your account, locate the manuscript record, and check for the action link "View Attachments". If this link does not appear, there are no attachment files.]

To ensure your figures meet our technical requirements, please review our figure guidelines: https://journals.plos.org/plosone/s/figures

You may also use PLOS’s free figure tool, NAAS, to help you prepare publication quality figures: https://journals.plos.org/plosone/s/figures#loc-tools-for-figure-preparation.

NAAS will assess whether your figures meet our technical requirements by comparing each figure against our figure specifications.

# Author comment: Author response to Decision Letter 1

**DOI:** 10.1371/journal.pone.0356238.r002

20 May 2026

Response to Comments

We thank the editor and reviewers for their constructive comments. We have carefully followed their suggestions and addressed the raised issues. Detailed responses to the comments are as follows. Major revisions are marked in RED in the revised manuscript.

A. Responses to Reviewer 1:

General Comment: This manuscript presents FusionPHI, a deep learning model for phage–host interaction (PHI) prediction based on multi-modal feature fusion: genomic k-mer frequencies, protein physicochemical properties, and motif-level embeddings derived from ESM-2, integrated via a dual-stage attention-driven feature fusion module. The authors benchmark FusionPHI against several existing PHI tools and include both ablation studies and a small experimental case study with M13K07 and several E. coli strains.

The topic is timely and clearly within the scope of PLOS ONE. The study appears to report original research and the approach is promising. However, there are important issues regarding methodological clarity, data availability, and explanation of the model to a biological audience that, in my view, require substantial revision.

First, the strengths of the manuscript:

Phage–host prediction remains a key bottleneck for interpreting metagenomic data and for phage therapy applications. A model that integrates multiple sequence-derived modalities is highly relevant to the community. The authors provide a good introduction that is consistent with the current state of knowledge, both regarding the challenges of phage therapy and the relevant bioinformatics problems.

The idea of combining: genomic k-mer features, aggregated protein physicochemical descriptors, and motif-level embeddings derived from a large protein language model (ESM-2) is conceptually appealing and could potentially capture complementary aspects of phage–host compatibility. New algorithms addressing the phage–host matching problem are very important.

The dual-stage self-/cross-attention feature fusion module is an interesting design choice for weighting and integrating heterogeneous feature types. The inclusion of ablation experiments to dissect the contribution of different feature sets and attention blocks is a clear strength.

The experimental infection assays with M13K07 and several E. coli strains, although limited in scope, are a welcome attempt to connect computational predictions with real biological behavior.

The authors provide a GitHub repository with the implementation of FusionPHI, which is an important step towards reproducibility.

Response: We thank the reviewer for the careful reading and for the positive overall assessment of our work. We are encouraged that the reviewer finds the topic timely and relevant, and that the design of FusionPHI—particularly the integration of multi-modal sequence-derived features and the attention-based fusion strategy—is considered meaningful. We also appreciate the recognition of our ablation analyses, preliminary experimental validation, and the public release of the code. We will carefully address the points raised in the following revisions.

Comment 1: The manuscript repeatedly states that motif embedding vectors constitute an “innovative feature”:

“In particular, we introduce an innovative feature, that is, the embedding vectors of the gene motif sequence.”

However, the novelty is not clearly defined or justified.

Protein language models (including ESM-2) have already been used to compute embeddings for protein sequences and sometimes for protein fragments/domains.

It is not clear in what precise sense motif-level embeddings are conceptually new compared with: embedding entire ORFs or full protein sequences, or using other fragment-based embeddings.

Please more clearly position FusionPHI relative to other recent multi-modal or attention-based PHI methods: which components are conceptually new (e.g. the dual-stage cross-attention design, use of motif-level ESM-2 embeddings), and which are extensions of earlier work?

Response: Thanks for your insightful comment. We completely agree that the submitted manuscript did not sufficiently articulate the precise novelty of our motif-level embeddings and the clear positioning of FusionPHI relative to existing methods. We have comprehensively addressed these points in the revised manuscript and detailed them below.

Regarding the specific novelty of motif-level embeddings: While ESM-2 has indeed been widely utilized to compute embeddings for full ORFs or entire protein sequences, these global representations often dilute the specific, localized signals that dictate phage-host interactions. On the other hand, embedding arbitrary sequence fragments lacks defined biological relevance. The conceptual novelty of our approach lies in specifically isolating functional motifs—which represent highly conserved, biologically significant interaction units—and encoding them as an independent modality. Unlike full-sequence embeddings that contain noise from non-interacting regions, or fragment embeddings that lack functional context, our motif-level embeddings force the language model to capture the concentrated semantic and structural features of the actual binding interfaces.

Regarding the positioning of FusionPHI (Innovation vs. Extension): To provide a transparent comparison with other recent multi-modal or attention-based PHI methods, we have explicitly delineated our novel contributions from the components that extend prior work:

Extensions of earlier work: The utilization of ESM-2 as a foundational feature extractor, the general concept of integrating multi-modal data for PHI prediction, and the baseline application of attention mechanisms represent extensions of established methodologies in the field.

Conceptual and Methodological Innovations: The primary innovations of FusionPHI are twofold. First is the conceptual introduction of motif-level ESM-2 embeddings as a distinct, specialized modality, which to our knowledge is the first application of its kind in PHI prediction. Second is the methodological design of the dual-stage self-/cross-attention fusion architecture. This specific mechanism is innovatively tailored to effectively align and integrate the highly localized, functionally dense motif signals with broader global sequence features, overcoming the limitations of standard attention modules that struggle to balance local functional units with global contexts.

We have incorporated these detailed clarifications into the Introduction section of the revised manuscript to ensure a transparent positioning of our work. We appreciate your rigorous feedback, which has significantly strengthened the theoretical foundation of our paper.

Comment 2: The text mentions that “27 original features” per sample are expanded to 81-dimensional vectors, but it is not initially clear what these 27 features are. From the Methods, one can infer that they correspond to:

7 physicochemical indices (length, pI, molecular weight, aromaticity, instability index, flexibility, GRAVY), plus

20 amino acid composition frequencies.

I suggest explicitly stating this in the text before introducing the expansion to 81 dimensions, so that the origin of the “27 features” is transparent.

Response: Thanks for pointing out this lack of clarity. In the “Physicochemical properties based proteomic feature encoding” subsection of the revised manuscript, we have explicitly defined the “27 original features” at their first occurrence, clearly stating that they consist of 7 physicochemical indices (sequence length, isoelectric point, molecular weight, aromaticity, instability index, flexibility, and GRAVY) together with 20 amino acid composition frequencies. We have also clarified the subsequent feature transformation process, explaining how these 27 features are expanded into an 81-dimensional representation. These revisions are intended to make the origin and construction of the feature vector fully transparent to the reader.

Comment 3: The model uses k = 4 for genomic k-mer frequencies, but the choice of this value is not justified.

Were other k values (e.g. 3, 5, or combinations) tested?

Is k = 4 chosen based on previous work on the same dataset, or on a systematic performance comparison?

Given that k-mer choice can substantially affect performance and feature dimensionality, please either:

justify k = 4 with references and/or include a brief sensitivity analysis showing that k = 4 is a reasonable choice.

Response: We thank the reviewer for this important comment. In the “K-mer frequency based genomic feature encoding” subsection of the revised manuscript, we have clarified the rationale for selecting k=4 by incorporating relevant references from prior studies, where this choice has been empirically validated as a balanced setting for capturing informative sequence patterns while controlling feature dimensionality in the existing PHI work like PHP and HostPhinder. We have revised the text to explicitly state that our selection is consistent with established practice in related genomic and PHI prediction studies. These additions are intended to provide a clearer justification for the use of k=4 in our model.

Comment 4: The manuscript states:“Subsequently, in order to more accurately capture the protein functional properties of these conserved regions, we performed transcription and translation operations on each motif mk to generate its corresponding RNA sequence rk and protein sequence pk, respectively.”

If motifs are translated without strict alignment to the original ORF frame, ESM-2 may be embedding artificial peptide sequences, which could compromise the biological meaning of the features.

Please clarify the exact procedure: how motifs are mapped to ORFs, how the reading frame is determined, and how non-frame or non-multiples-of-three motifs are handled (filtered, trimmed, or still translated).

Response: Thanks for this important and technically precise question. We agree that the submitted manuscript did not sufficiently describe how motif sequences are handled during the transcription and translation steps.

First, regarding the mapping between motifs and ORFs, we clarify that in our current framework motifs are not explicitly mapped back to annotated ORFs, nor are they constrained to lie within a known coding frame. Instead, motifs identified by the MEME suite are treated as conserved nucleotide patterns at the genome level, independent of gene annotation. Our goal is not to reconstruct native coding sequences, but to extract evolutionarily conserved local signals and project them into amino acid space for downstream representation learning.

Second, concerning the reading frame, we do not attempt to infer or enforce a biologically “correct” reading frame for each motif. Rather, a fixed and consistent translation rule is applied to all motifs. This design reflects the fact that motif boundaries are not guaranteed to align with codon structure, and enforcing a frame could introduce additional uncertainty or bias. We have revised the manuscript to explicitly state that the translation step is used as a feature transformation rather than a reconstruction of true protein sequences.

Third, for motifs whose lengths are not multiples of three, we clarify that no filtering or trimming is applied. Instead, motifs are directly translated under the same rule, allowing partial codons at the boundaries to be handled consistently (i.e., truncated during translation if necessary). This ensures that all identified motifs contribute to the representation, while maintaining a uniform processing pipeline.

We have added a detailed description of these points in the revised “Gene motif based genomic feature encoding” subsection and clarified the wording to avoid any implication of strict ORF-aware translation. We also explicitly state that the resulting amino acid sequences should be interpreted as abstracted representations of conserved sequence patterns, rather than biologically expressed peptides.

Comment 5: The dual-stage attention-driven fusion module (self-attention followed by two cross-attention blocks) is central to FusionPHI. The current description is mathematically correct but quite difficult to follow for readers without a deep learning background.

I recommend adding a brief, intuitive explanation, for example:

what self-attention is expected to capture within each modality (e.g. which k-mers or motif features are most informative), what cross-attention is expected to capture between modalities (e.g. aligning specific motif patterns with particular genomic patterns or protein properties), how the two stages complement each other.

A small schematic or a simple toy example would substantially improve accessibility for biologists and bioinformaticians not specializing in ML.

Response: Thanks for this helpful suggestion. We agree that the original description of the dual-stage attention module was relatively difficult to follow for readers without a deep learning background, despite being mathematically correct.

In the “Dual-stage attention-driven feature fusion” subsection of the revised manuscript, we have added an intuitive explanation of the attention mechanism using a Query–Key–Value analogy (similar to a database retrieval process). This is intended to provide a conceptual understanding of how the model dynamically assigns importance to different features, making the underlying idea more accessible to non-specialists.

We now explicitly clarify the role of self-attention in our framework. Specifically, self-attention operates within each modality to capture intra-modality dependencies, such as identifying which k-mer patterns, physicochemical properties, or motif-derived features are most informative, while down-weighting less relevant or noisy signals.

We further expand the explanation of cross-attention by emphasizing its role in modeling inter-modality relationships. In particular, we describe how the model aligns global genomic and proteomic features with conserved motif patterns, enabling the integration of complementary biological signals across modalities.

Finally, we clarify how the two stages complement each other: self-attention refines modality-specific representations, while cross-attention bridges these representations to form a unified and biologically meaningful feature space. To further improve accessibility, we have incorporated schematic figures and a simplified explanatory example in the revised manuscript, which together aim to make the framework more interpretable for a broader biological and bioinformatics audience.

Comment 6: The manuscript states that ORF sequences or gene sequences are used, but it is not sufficiently clear how these were obtained:

Were ORFs taken from existing annotations in NCBI/RefSeq or from the PHP dataset?

Were they predicted de novo (e.g. using NCBI ORFfinder, Prodigal, or another tool)? If so, with which parameters?

Response: Thanks for your insightful comments regarding data provenance and ORF acquisition, and we agree that the submitted manuscript did not describe this aspect with sufficient clarity.

In our study, we did not directly use the sequence data provided within the original PHP dataset. Instead, we used the taxonomic information and accession IDs from that dataset to retrieve complete and standardized genomic sequences from the NCBI RefSeq database. This ensures consistency and data quality across all samples.

Regarding ORFs, we did not perform de novo prediction using tools such as ORFfinder or Prodigal. Rather, all ORF sequences and their corresponding protein translations were directly obtained from the official annotated records in RefSeq (e.g., associated .faa files). This design choice ensures that the protein sequences used for downstream feature extraction are based on curated and biologically validated annotations, rather than potentially noisy computational predictions.

To address the reviewer’s concern and improve transparency, we have added a detailed description of the data sources and retrieval procedures in the “Datasets” subsection of the revised manuscript, so that readers can clearly understand how genomic and ORF-level data were obtained and processed.

Comme

Attachment Submitted filename: Response to Reviewers.docx

# Peer review: Decision Letter 1

**DOI:** 10.1371/journal.pone.0356238.r003

**Authors:** Ivan Petrushin

30 Jun 2026

Dear Dr. Wei,

Thank you for submitting your revised manuscript to PLOS ONE. As an Editor, I would like to thank the authors for the improving the manuscript, but some concerns remain to be addressed. Therefore, we invite you to submit a revised version of the manuscript that addresses the points raised during the review process. We are sorry for the delay due to waiting for all three reviewers' comments.

Please submit your revised manuscript by Aug 14 2026 11:59PM. If you will need more time than this to complete your revisions, please reply to this message or contact the journal office at plosone@plos.org. When you're ready to submit your revision, log on to https://www.editorialmanager.com/pone/ and select the 'Submissions Needing Revision' folder to locate your manuscript file.

- A letter that responds to each point raised by the academic editor and reviewer(s). You should upload this letter as a separate file labeled 'Response to Reviewers'.
- A marked-up copy of your manuscript that highlights changes made to the original version. You should upload this as a separate file labeled 'Revised Manuscript with Track Changes'.
- An unmarked version of your revised paper without tracked changes. You should upload this as a separate file labeled 'Manuscript'.

If you would like to make changes to your financial disclosure, please include your updated statement in your cover letter. Guidelines for resubmitting your figure files are available below the reviewer comments at the end of this letter.

If applicable, we recommend that you deposit your laboratory protocols in protocols.io to enhance the reproducibility of your results. Protocols.io assigns your protocol its own identifier (DOI) so that it can be cited independently in the future. For instructions see: https://journals.plos.org/plosone/s/submission-guidelines#loc-laboratory-protocols. Additionally, PLOS ONE offers an option for publishing peer-reviewed Lab Protocol articles, which describe protocols hosted on protocols.io. Read more information on sharing protocols at https://plos.org/protocols?utm_medium=editorial-email&utm_source=authorletters&utm_campaign=protocols.

As the corresponding author, your ORCID iD is verified in the submission system and will appear in the published article. PLOS supports the use of ORCID, and we encourage all coauthors to register for an ORCID iD and use it as well. Please encourage your coauthors to verify their ORCID iD within the submission system before final acceptance, as unverified ORCID iDs will not appear in the published article. *Only*  the individual author can complete the verification step; PLOS staff *cannot*  verify ORCID iDs on behalf of authors.

We look forward to receiving your revised manuscript.

Kind regards,

Ivan S Petrushin, Ph.D

Academic Editor

PLOS One

Journal Requirements:

If the reviewer comments include a recommendation to cite specific previously published works, please review and evaluate these publications to determine whether they are relevant and should be cited. There is no requirement to cite these works unless the editor has indicated otherwise.

Please review your reference list to ensure that it is complete and correct. If you have cited papers that have been retracted, please include the rationale for doing so in the manuscript text, or remove these references and replace them with relevant current references. Any changes to the reference list should be mentioned in the rebuttal letter that accompanies your revised manuscript. If you need to cite a retracted article, indicate the article’s retracted status in the References list and also include a citation and full reference for the retraction notice.

[Note: HTML markup is below. Please do not edit.]

Reviewers' comments:

Reviewer's Responses to Questions

**Comments to the Author**

Reviewer #2: All comments have been addressed

Reviewer #3: (No Response)

Reviewer #4: (No Response)

**********

2. Is the manuscript technically sound, and do the data support the conclusions?

Reviewer #2: Yes

Reviewer #3: Partly

Reviewer #4: Partly

**********

3. Has the statistical analysis been performed appropriately and rigorously?

Reviewer #2: Yes

Reviewer #3: Yes

Reviewer #4: No

**********

4. Have the authors made all data underlying the findings in their manuscript fully available?

The PLOS Data policy

Reviewer #2: Yes

Reviewer #3: Yes

Reviewer #4: Yes

**********

5. Is the manuscript presented in an intelligible fashion and written in standard English?

Reviewer #2: Yes

Reviewer #3: Yes

Reviewer #4: Yes

**********

Reviewer #2: The authors have fully addressed all of my comments, and I see no reason to withhold the manuscript from publication in its current form.

Reviewer #3: Use of protein language models ESM2 in understanding the phage cost compatibility is a strength of the study.

1. Lacking the explainable AI part to understand the reason behind prediction which is relevant in biology

2. from the RefSeq .faa files whether all the gene annotations including hypothetical proteins are also used or not. It is not clearly mentioned in the manuscript

3. The dataset provided in the GitHub repository appears to be incomplete. The authors should consider depositing the complete dataset in a repository such as Zenodo. Additionally, one bacterial genome in the training dataset appears to be a partial genome (16S rRNA only). Please clarify whether partial genomes were included in the study and, if so, how they were handled.

4. The response to comment 4 from first reviewer explains the computational procedure but does not address the fundamental concern of the reviewer, whether arbitrarily translated motif sequences retain sufficient biological meaning to justify the use of ESM-2 protein embeddings.

5. The response to comment 8 from the first review only addresses pair level information leakage but does not sufficiently address the broader concerns regarding dataset construction, negative sampling, taxonomic distribution, data splitting strategy, and model generalizability.

6. Italicizing organism name throughout the manuscript

7. Please check the AI content of the manuscript

Reviewer #4: FusionPHI: a phage-host interaction prediction network model based on attention-driven multi-modal feature fusion

This manuscript presents FusionPHI, a multi-modal attention-driven framework for phage–host interaction (PHI) prediction. The proposed method integrates k-mer frequency features, protein physicochemical properties, and conserved motif embeddings via a dual-stage attention mechanism. The topic is timely and relevant, and the overall architecture is technically interesting. However, methodological, presentation, and consistency issues need to be addressed before the manuscript can be considered for publication.

**Major Comments**

1. Dataset inconsistencies and incomplete data usage

After consulting the original PHP study, the test set appears to contain 671 virus-host interaction pairs, not 672 as stated by the authors. This discrepancy should be clarified. More importantly, it is unclear why the authors used only the PHP test set rather than the full merged dataset combining the PHP study and VirHostMatcher interactions. Furthermore, the 335 additional interaction pairs reported by the PHP authors on their GitHub repository do not appear to have been incorporated. The authors should explicitly justify these data choices and clarify the exact composition of their training dataset.

2. Fairness of baseline comparisons (Table 2)

The comparative evaluation presents results for VHM, PHP, PHIAF, vHULK, PB-LKS, and PHPGAT sourced from Nie's review rather than obtained through independent re-evaluation. It is unclear whether all methods were assessed under identical experimental conditions and on the same dataset. If the reported performance values originate from different datasets or evaluation protocols, the observed performance gains may not be attributed to the proposed model alone. The authors must either rerun all baseline methods on the same dataset under the same conditions, or explicitly acknowledge this as a critical limitation of the comparison. Furthermore, Table 2 is missing AUC and MCC values for most baseline methods.

3. Cross-validation construction and internal data leakage

The authors do not describe how the five-fold cross-validation splits were constructed. Specifically, it is not stated whether phage or host identities were held out across folds to prevent within validation leakage.

4. Data leakage argument between PHP and PHIAF datasets is not fully convincing

The authors argue that no pair-level data leakage exists between the PHP training dataset and the PHIAF test dataset, based on the observation that host information is recorded differently across the two datasets, species names in PHP versus genome accession identifiers in PHIAF. However, this argument is not fully convincing, as different representations do not necessarily imply different biological entities. For example, a species name such as Escherichia coli in PHP and a genome accession in PHIAF could refer to the same organism. To properly rule out leakage, the authors should map the PHIAF host accession identifiers to their corresponding species names and explicitly verify that no overlapping phage-host pairs exist at the biological level, not merely at the label level. Additionally, the authors state that no "identical" phage-host pairs exist across datasets, but it is unclear whether "identical" refers strictly to exact label matches or also accounts for genome sequence similarity, which could also introduce leakage.

5. Case study results contradict the paper's main claims

The case study in its current form undermines rather than supports the paper's central contribution. As shown in Table 4, the ablated model Attn-KP, which excludes motif embeddings, correctly predicts all five phage–host interaction pairs, while FusionPHI produces incorrect predictions for DH5α and Rosetta⋆(DE3)plys. The authors attribute FusionPHI's errors to data quality issues arising from manual sequence splicing introducing false motif signals. While this explanation is plausible, it simultaneously highlights a critical fragility of the motif feature, the paper's primary novelty. The authors should provide a more thorough analysis of when motif features contribute positively versus when they introduce noise, and this vulnerability should be explicitly acknowledged in the Limitations section.

6. Positive-to-negative sample ratio

The authors use a 1:1 ratio of positive to negative PHI samples. While this is a common choice for balanced classification, it does not reflect the biological reality, where true phage-host interactions represent a small fraction of all possible pairs. The authors should discuss whether and how this imbalance affects the generalisability of the model to real-world screening scenarios.

**Minor Comments**

Line 16: The phrase "predict whether an interaction exists is likely to occur" is unclear.

Line 17: "in vivo" should be italicised, as is standard for Latin terms in scientific writing. The same applies to all instances of de novo (line 186) and any other Latin terms throughout the manuscript, including those appearing in figures.

Line 32: The inclusion of PhiSpy appears out of place. PhiSpy is designed to identify prophage regions within bacterial genomes, which is a distinct task from PHI prediction. The authors should either clarify how PhiSpy relates to the PHI prediction pipeline or relocate it to a more appropriate context.

Line 38: Reference (16) is cited without author attribution ("research by (16) has demonstrated..."). At minimum, the first author's name should be included, e.g. "Smith et al. (16) demonstrated...".

Line 130: The term "de-emphasis" is unclear.

Lines 134 and 165: Line 134 states the final dataset contains "333 phage genomes", while line 165 states "the PHP dataset contains 336 unique phage genomes". This inconsistency should be resolved and explained.

Lines 156–161: The repeated genus names (e.g. "Mycolicibacterium (Mycobacterium)", "Shigella (Shigella genus)") are redundant.

Line 164: "information leakage" should be replaced with "data leakage".

Line 245: A period seems to be missing.

Line 263: The extended acronym for PHI is reintroduced unnecessarily, having already been defined earlier.

Figure 3 caption: The sentence "High-dimensional embedding representations were extracted." appears to be a redundant leftover fragment.

Figure 5 caption: typo "corss-attention".

Figures 7A–7E: The image quality is insufficient. The authors should provide higher resolution images and consider adding annotations to guide the reader.

Line 401: During the comparison against the PHIAF dataset, it should be clarified whether all models were evaluated on the same test set, with only the classification model varying. This needs to be made explicit to ensure the comparison is interpretable.

Conclusions, line 569: The stated AUC of 91% is inconsistent with Table 3, which reports an AUC of 0.87 ± 0.012 for the full model. Please verify and correct.

**********

what does this mean?). If published, this will include your full peer review and any attached files.

If you choose “no”, your identity will remain anonymous but your review may still be made public.

**Do you want your identity to be public for this peer review?**  For information about this choice, including consent withdrawal, please see our Privacy Policy

Reviewer #2: **Yes:** Dmitry Penzar

Reviewer #3: No

Reviewer #4: No

**********

[NOTE: If reviewer comments were submitted as an attachment file, they will be attached to this email and accessible via the submission site. Please log into your account, locate the manuscript record, and check for the action link "View Attachments". If this link does not appear, there are no attachment files.]

To ensure your figures meet our technical requirements, please review our figure guidelines: https://journals.plos.org/plosone/s/figures

You may also use PLOS’s free figure tool, NAAS, to help you prepare publication quality figures: https://journals.plos.org/plosone/s/figures#loc-tools-for-figure-preparation.

NAAS will assess whether your figures meet our technical requirements by comparing each figure against our figure specifications.

# Author comment: Author response to Decision Letter 2

**DOI:** 10.1371/journal.pone.0356238.r004

21 Jul 2026

Ivan S Petrushin, Ph.D

Academic Editor

PLOS One

20 July, 2026

Re: Manuscript reference No.: PONE-D-25-60434R1

Dear Professor Ivan S Petrushin:

Attached please find our revised manuscript entitled “FusionPHI: a phage-host interaction prediction network model based on attention-driven multi-modal feature fusion”, which we would like to resubmit for publication in PLOS One.

We would like to once again express our sincere gratitude to you and the reviewers for the continued rigorous evaluation of our work. The second-round comments were, like the first, highly insightful and constructive, and addressing them has enabled us to further strengthen the manuscript. In the following pages, we provide detailed point-by-point responses to each of the reviewers’ additional comments. All major revisions in the revised manuscript are marked in RED for ease of identification.

We also wish to confirm that we have maintained full compliance with the journal’s formatting and policy requirements throughout this revision. The manuscript continues to adhere to the PLOS One style templates, and all funding, data availability, and submission system details have been kept consistent with the journal’s standards, as updated in the previous round. No funding-related text appears in the manuscript body. Regarding data availability, in addition to maintaining our GitHub repository for the source code, we have now deposited the complete dataset, including all sequence files, metadata, and processing records, in a permanent Zenodo repository(DOI: 10.5281/21255334), as recommended by the reviewers. The GitHub repository has been updated to direct users to this stable archive, and the Data Availability Statement in the manuscript has been revised accordingly. We have also retained the non-author institutional contact for any further data access inquiries, ensuring full compliance with PLOS One’s Data Policy.

In summary, we believe that the manuscript has been substantially improved through both rounds of review and now exhibits greater methodological rigor, transparency, and clarity of presentation. We sincerely hope that the current version, together with our accompanying responses, will be deemed suitable for publication in PLOS One. Should any further revisions be required, we remain fully committed to addressing them promptly. Thank you once again for your time, expertise, and invaluable guidance.

Yours sincerely,

Qingting Wei

School of Software

Nanchang University

235 East Nanjing Road, Nanchang 330047, Jiangxi Province, China

Email: qtwei@ncu.edu.cn

Dear Editor and Reviewers,

We sincerely thank the editor and reviewers for their continued rigorous and constructive feedback during this second round of review. We have carefully considered all the additional comments and have thoroughly revised the manuscript accordingly. Detailed point-by-point responses are provided below. As before, all major revisions in the revised manuscript are highlighted in RED for ease of identification.

A. Responses to Reviewer #3:

Comment 1: Lacking the explainable AI part to understand the reason behind prediction which is relevant in biology.

Response: We sincerely thank the reviewer for this insightful and constructive comment. We fully agree that, for a biological problem such as phage–host interaction prediction, simply reporting predictive performance is insufficient and understanding the reasoning behind the predictions is essential for both model credibility and biological discovery. We are grateful that the reviewer raised this point, as it prompted us to substantially strengthen the interpretability of our work.

In the revised manuscript, we have added a dedicated section entitled “Attention-based Feature Interaction Analysis” to directly address this concern. In this section, we designed a multi-metric feature importance evaluation framework that combines four complementary measures: Pearson correlation, Random Forest Gini importance, permutation importance, and mutual information. All importance scores were normalized to enable fair comparison, and the results are visualized as a heatmap in Fig 7.

We also conducted a comprehensive analysis on the patterns of feature importance across different evaluation metrics and yielded several insights:

1) CG-enriched k-mer sequence features (e.g., the frequencies of CAGC, TCAG, CAGG, CCAG) show consistently high importance across all four metrics, indicating that they are robust and biologically meaningful signals for prediction.

2) Physicochemical features derived from amino acid compositions (e.g., the AAC frequencies for the amino acids D and G) display only moderate linear correlation with the labels, yet they achieve notably high permutation importance, suggesting that their predictive value is expressed through non-linear relationships that would have been missed by correlation-based selection alone.

3) Motif features (e.g., host_feature_96, host_feature_348, host_feature_264) exhibit balanced and moderate importance across all metrics, implying that they provide complementary information at the functional architecture level rather than relying on any single dominant signal.

Taken together, these findings demonstrate that our model integrates diverse feature modalities in a non-redundant manner, and, more importantly, that its predictions can be traced back to recognizable biological factors such as sequence motifs, physicochemical properties, and functional domains. We hope this addition satisfies the reviewer's request for explainable AI in this biological context.

We appreciate the reviewer's constructive feedback, which has significantly improved the manuscript's interpretability and biological relevance.

Comment 2: from the RefSeq .faa files whether all the gene annotations including hypothetical proteins are also used or not. It is not clearly mentioned in the manuscript

Response: We sincerely thank the reviewer for this careful and constructive comment. We apologize that this point was not stated clearly enough in the original manuscript.

In this study, host protein-coding sequences were obtained from the latest RefSeq assemblies. For each host species, we retrieved the assembly-provided CDS files and translated all coding sequences into amino-acid sequences. No filtering based on functional annotation was applied at either the nucleotide or the protein level. Consequently, the complete set of RefSeq CDS annotations was retained, including hypothetical proteins.

These sequences were then used to compute physicochemical features, after which the multi-ORF features were aggregated into a host-level representation. We revise the manuscript to describe this procedure explicitly in the last paragraph of the “Datasets” subsection so that the use of all annotated gene products, including hypothetical proteins, is unambiguously documented.

Comment 3: The dataset provided in the GitHub repository appears to be incomplete. The authors should consider depositing the complete dataset in a repository such as Zenodo. Additionally, one bacterial genome in the training dataset appears to be a partial genome (16S rRNA only). Please clarify whether partial genomes were included in the study and, if so, how they were handled.

Response: We sincerely thank the reviewer for this careful observation and the constructive suggestion. We apologize for the incomplete dataset initially shared on GitHub and this has now been fully rectified. The complete dataset, including all sequence files, metadata, and processing records, has been deposited in a permanent Zenodo repository (DOI: 10.5281/21255334), and the GitHub repository has been updated to clearly direct users to this stable archive.

Regarding the inclusion of partial genomes, we appreciate the opportunity to clarify. During dataset construction, we prioritized retrieving complete genomes from NCBI. However, for several rare taxa of particular phylogenetic interest, no complete genome was available. To avoid introducing taxonomic gaps, we carefully retained high-quality partial genomes only when a complete alternative could not be found. The 16S rRNA-only sequence noted by the reviewer originated from a targeted locus project and was the sole representative for that species. Prompted by the reviewer’s comment, we carefully re-evaluated the quality and completeness of all partial sequences used in the study. For the 16S rRNA-only sequence, no higher-quality complete genome could be located, and removing it would have introduced a taxonomic gap. Therefore, we retained it but now explicitly acknowledge in the third paragraph of the “Limitations” subsection of the revised manuscript that partial genomes were included when complete genomes were unavailable, which may introduce potential biases.

We are deeply grateful to the reviewer for pointing out this issue, as addressing it has made our data selection criteria more transparent and rigorous.

Comment 4: The response to comment 4 from first reviewer explains the computational procedure but does not address the fundamental concern of the reviewer, whether arbitrarily translated motif sequences retain sufficient biological meaning to justify the use of ESM-2 protein embeddings.

Response: We sincerely thank you for this follow‑up comment, which urges us to address the deeper concern rather than merely the procedural details. We fully understand the worry that arbitrarily translated motif sequences, produced without respecting the native reading frame, may carry little biological meaning and thus call into question the use of ESM‑2 protein embeddings. We appreciate the opportunity to clarify our perspective on this point.

We completely agree that the resulting amino acid sequences are not genuine proteins, and we would never claim that they represent biologically expressed peptides. However, our aim has never been to model protein function or structure. Instead, we use the translation step as a consistent, information‑preserving transformation that maps conserved nucleotide patterns into an amino acid alphabet, solely for the purpose of generating dense feature representations via a pretrained model. ESM‑2 embeddings are known to capture general physicochemical properties (such as hydrophobicity, charge, and size) as well as contextual dependencies of amino acids, even for sequences that do not correspond to natural proteins. Because the motifs identified by MEME are themselves evolutionarily conserved nucleotide segments, forcing them through a fixed translation frame still retains the local sequence composition and order in a different symbolic space, which ESM‑2 can then encode into a rich feature vector. We do not treat these embeddings as functional annotations. Actually they serve as abstract representations of conserved genomic signals, much like k‑mer or one‑hot encodings, but with the advantage of leveraging the pretrained language model’s ability to capture higher‑order dependencies.

We have taken the reviewer’s fundamental concern very seriously. In the fourth paragraph of the “Limitations” subsection of the revised manuscript, we have substantially expanded the discussion to explicitly acknowledge the limitations: we now clearly state that the translated sequences are not biologically valid proteins, that the enforced reading frame may introduce artificial boundaries, and that the embeddings should be interpreted only as transformed features of conserved nucleotide motifs. We also note that, in the absence of direct experimental validation of whether these embeddings retain meaningful biological signal for the downstream task, this approach is best viewed as a pragmatic representation‑learning strategy that showed empirical benefit in our predictive task. We fully agree that further studies, possibly involving systematic ablation or comparison with random‑frame controls, would be valuable to rigorously quantify the biological relevance, and we have included this as a future direction.

We are deeply grateful to the reviewer for insisting on this fundamental issue, as it has led us to greatly improve the clarity and honesty of our presentation, and we hope our revised framing adequately addresses the concern.

Comment 5: The response to comment 8 from the first review only addresses pair level information leakage but does not sufficiently address the broader concerns regarding dataset construction, negative sampling, taxonomic distribution, data splitting strategy, and model generalizability.

Response: We sincerely thank the reviewer for this comment and for requiring a more comprehensive clarification of these interrelated aspects of our study. We fully acknowledge that our earlier response focused too narrowly on the information leakage issue, and we apologize for not addressing the other concerns with sufficient breadth and detail. We are grateful for the opportunity to provide a unified account here, which we have also reflected in the revised manuscript.

Dataset construction. The training dataset is built upon the publicly available PHP dataset, which provides 335 experimentally verified phage–host interaction pairs. We acknowledge that a processing oversight led to one positive pair (phage NC_042345 and host Xylella fastidiosa) being inadvertently duplicated, making the positive set 336 in the original manuscript. This error has been corrected, and the dataset now correctly comprises 335 positive pairs and 335 negative pairs (1:1 ratio). We have thoroughly re‑verified all sequence files, accession numbers, and pair counts, and updated the manuscript accordingly to ensure full consistency. More Detailed information can be seen in the Datasets subsection of the revised manuscript.

Negative sampling. In our study, negative pairs were generated by randomly pairing phages and hosts that are not known to interact, maintaining a 1:1 ratio with the positive pairs. We fully recognize that this balanced sampling strategy, while commonly adopted to ensure stable model training and unbiased evaluation, does not reflect the biological reality where true phage–host interactions represent only a very small fraction of all possible virus–host pairs. This discrepancy may lead the model to produce overconfident positive predictions when applied to severely imbalanced real-world screening scenarios. We have explicitly discussed this limitation in the revised “Limitations” subsection, where we state: “we adopted a balanced 1:1 ratio of positive to negative pairs during training. While this is a common practice for stable model optimization, it does not reflect the biological reality... the model may tend to produce overconfident positive predictions when deployed in real-world screening scenarios with severe class imbalance, and its practical utility in such settings remains to be systematically evaluated.” We believe this transparent acknowledgment appropriately conveys the current scope of our model.

Taxonomic distribution. We recognize that the dataset exhibits a long‑tailed taxonomic distribution: certain host genera (e.g., Escherichia, Pseudomonas, Staphylococcus) are heavily overrepresented, while many genera are represented by only one or a few instances. This imbalance may introduce taxonomic bias and lead to overestimation of performance on dominant taxa. We have addressed this concern in the revised manuscript by explicitly reporting the taxonomic composition of both the PHP and PHIAF datasets (summarized in Fig 1 and Fig 2), discussing the potential impact on model generalization, and identifying the improvement of taxonomic balance as a key objective for future data expansion.

Data splitting strategy. For internal evaluation, we employed five‑fold cross‑validation using scikit‑learn’s KFold with shuffle=True and a fixed random seed (random_state=42). The splits were performed at the sample (pair) level, without enforcing grouping by phage or host identity. We fully acknowledge that this design may allow instances sharing the same phage or host to appear across training and validation folds, introducing a potential risk of within‑validation data leakage. However, we wish to emphasize that, in our study, five‑fold cross‑validation was used exclusively for hyperparameter

Attachment Submitted filename: Response_to_Reviewers_auresp_2.docx

# Editor report: Decision Letter 2

**DOI:** 10.1371/journal.pone.0356238.r005

**Authors:** Ivan Petrushin

3 Aug 2026

FusionPHI: a phage-host interaction prediction network model based on attention-driven multi-modal feature fusion

PONE-D-25-60434R2

Dear Dr. Wei,

I'm sorry for the delay due to the vacation season. We’re pleased to inform you that your manuscript has been judged scientifically suitable for publication and will be formally accepted for publication once it meets all outstanding technical requirements. I would thank the authors for the efforts improving the manuscript, but please consider my extra comments at the end of the message.

Within one week, you’ll receive an e-mail detailing the required amendments. When these have been addressed, you’ll receive a formal acceptance letter and your manuscript will be scheduled for publication.

An invoice will be generated when your article is formally accepted. Please note, if your institution has a publishing partnership with PLOS and your article meets the relevant criteria, all or part of your publication costs will be covered. Please make sure your user information is up-to-date by logging into Editorial Manager at Editorial Manager® and clicking the ‘Update My Information' link at the top of the page. For questions related to billing, please contact billing support.

If your institution or institutions have a press office, please notify them about your upcoming paper to help maximize its impact. If they’ll be preparing press materials, please inform our press team as soon as possible -- no later than 48 hours after receiving the formal acceptance. Your manuscript will remain under strict press embargo until 2 pm Eastern Time on the date of publication. For more information, please contact onepress@plos.org.

Kind regards,

Ivan S Petrushin, Ph.D

Academic Editor

PLOS One

Some minor issues has to be resolved along  final version to be proceeded:

- usage example (with test dataset) in the FusionPHI repository might be helpful for the readers

- please specify, are the software package requirements strict or could be changed to more recent version (e.g. MEME Suite has ver. 5.5.9 now instead of 5.5.5 used in the FusionPHI repository)

- check the spelling, "moitf sequence" in README

- see L56 and Table 2: species name has to be italic

- more recent reference for ESM-2 [ref. 28] is https://doi.org/10.1126/science.ade2574 with https://github.com/facebookresearch/ESM (if appropriate)

- subheading at L222 tells about "Physicochemical properties ...", but the amino acid composition (which is different protein feature) is also covered in this subsection. Please consider the revising the heading or subsection structure.

- Zenodo repository (DOI: 10.5281/21255334) is unavailable, please check the DOI or provide a correct direct link to Zenodo record (https://doi.org/10.5281/zenodo.21255334).

# Editor report: Acceptance letter

**DOI:** 10.1371/journal.pone.0356238.r006

**Authors:** Ivan Petrushin

PONE-D-25-60434R2

PLOS One

Dear Dr. Wei,

I'm pleased to inform you that your manuscript has been deemed suitable for publication in PLOS One. Congratulations! Your manuscript is now being handed over to our production team.

At this stage, our production department will prepare your paper for publication. This includes ensuring the following:

* All references, tables, and figures are properly cited

* All relevant supporting information is included in the manuscript submission,

* There are no issues that prevent the paper from being properly typeset

You will receive further instructions from the production team, including instructions on how to review your proof when it is ready. Please keep in mind that we are working through a large volume of accepted articles, so please give us a few days to review your paper and let you know the next and final steps.

Lastly, if your institution or institutions have a press office, please let them know about your upcoming paper now to help maximize its impact. If they'll be preparing press materials, please inform our press team within the next 48 hours. Your manuscript will remain under strict press embargo until 2 pm Eastern Time on the date of publication. For more information, please contact onepress@plos.org.

You will receive an invoice from PLOS for your publication fee after your manuscript has reached the completed accept phase. If you receive an email requesting payment before acceptance or for any other service, this may be a phishing scheme. Learn how to identify phishing emails and protect your accounts at https://explore.plos.org/phishing.

If we can help with anything else, please email us at customercare@plos.org.

Thank you for submitting your work to PLOS ONE and supporting open access.

Kind regards,

PLOS ONE Editorial Office Staff

on behalf of

Dr. Ivan S Petrushin

Academic Editor

PLOS One
